# New Triazolyl N^N Bidentate Rh(III), Ir(III), Ru(II) and Os(II) Complexes: Synthesis and Characterization, Probing Possible Relations between Cytotoxicity with Transfer Hydrogenation Efficacy and Interaction with Model Biomolecules

**DOI:** 10.3390/molecules27072058

**Published:** 2022-03-23

**Authors:** William K. Chu, Charles K. Rono, Banothile C. E. Makhubela

**Affiliations:** Research Centre for Synthesis and Catalysis, Department of Chemical Sciences, Auckland Park Campus, University of Johannesburg, Johannesburg 2006, South Africa; onekind21@gmail.com (W.K.C.); ronock13@gmail.com (C.K.R.)

**Keywords:** transfer hydrogenation, cancer, click chemistry, catalysis, chemotherapy, triazole

## Abstract

Cisplatin and other metallodrugs have realised great success in clinical chemotherapeutic applications as anticancer drugs. However, severe toxicity to healthy cells and non-selectivity to cancer cells remains a challenge, warranting the further search for alternative agents. Herein, we report the anticancer potential of a series of complexes of the general formula [MCl(*p-cym*)(*k*^2^-N^N-L)]^+^ X^−^ and [MCl(Cp*)(*k*^2^-N^N-L)]^+^ X^−^, where M is the metal centre (Ru(II), Os(II), Rh(III) or Ir(III)), L = 1-benzyl-4-pyridinyl-1-*H*-1,2,3-triazole for **L1** and 1-picolyl-4-pyridinyl-1-*H*-1,2,3-triazole for **L2** and X^−^ = Cl^−^, BF_4_^−^, BPh_4_^−^. When evaluated for activity against some cancerous and non-cancerous cell lines (namely, HeLa, HEK293, A549 and MT4 cancer cells and the normal healthy kidney cells (BHK21)), most of the compounds displayed poor cytotoxicities against cancer cells except for complexes **C2** ([RuCl(*p-cym*)(*k*^2^-N^N-**L1**)]^+^ BPh_4_^−^, EC_50_ = 9–16 µM and SI = 14), **C7** ([RuCl(*p-cym*)(*k*^2^-N^N-**L2**)]^+^ BPh_4_^−^, EC_50_ = 17–53 µM and SI = 4) and **C11** ([IrCl(Cp*)(*k*^2^-N^N-**L2**)]^+^ BF_4_^−^, EC_50_ < 5 µM and SI > 10). Selected complexes **C1** ([RuCl(*p-cym*)(*k*^2^-N^N-**L1**)]^+^ BF_4_^−^), **C5** ([IrCl(Cp*)(*k*^2^-N^N-**L1**)]^+^ BF_4_^−^) and **C11** showed significant interactions with model biomolecules such as guanosine-5′-monophosphate (5′-GMP), bovine serum albumin (BSA) and amino acids under physiological conditions, possibly through carbenylation and *N*-coordination with 5′-GMP, *N*-coordination with L-Histidine and L-proline. While the compounds showed good activities in reducing pyruvate to lactate, there was no direct correlation between catalytic transfer hydrogenation of pyruvate and the observed cytotoxic activities. As observed in this work, the marked influence of single atom replacement in ligand may provide a pivotal approach to improving the cytotoxicity and fine-tuning the selectivity to cancer cells.

## 1. Introduction

Cancer is a collection of diseases arising from the sequential mutation in the functioning of essential genes responsible for coordinating cell proliferation and apoptosis [1,2]. These diseases all share the characteristics of being able to become autonomous and invasive. The International Agency for Research on Cancer (IARC) estimates that by 2040, there will be 27.5 million new cancer cases yearly, with an associated 16.3 million deaths [1,2,3].

Among the several therapeutic options that exist, chemotherapy has been widely used as a treatment option [1,2,4]. However, several challenges are associated with this approach. Among them are poor selectivity towards normal cells, poor specificity against a given cancer cell line, induced drug resistance towards cells, and difficulties in assessing the mode of action of the chemotherapeutic agent, which includes cisplatin [5,6,7,8].

The use of metal complexes over organic molecules in cancer chemotherapy is of increasing interest due to their ability to adopt different conformations with diverse electronic properties and generate molecules with a unique mode of action [9]. Such a reactivity profile allows interaction with biomolecules, and can be explored in the in vitro catalytic transformation of biologically important molecules as a potential anticancer mechanism [7,9,10].

Transfer hydrogenation using sodium formate and/or NADH has been studied as a hydride source in a modelled aqueous system [7,10,11] to reveal the mode of action of metallodrug candidates. If the mode of action is known, catalytic amounts can be administered to sufficiently lower toxicity while improving specificity and minimizing drug resistance. In agreement with this, a direct correlation was achieved between cytotoxicity, selectivity towards normal cells, and catalytic activity in which pyruvate was hydrogenated using NADH or sodium formate as a hydrogen source [7,10]. A possible hypothesis here is that increased lactate accumulation in cancer cells would lead to acidosis and apoptosis under acute conditions. In contrast, it will be stored as glycogen in normal cells, since normal cells are adapted to maintaining physiological balance [12]. A second reason is that altering the cell’s redox status will give normal cells a physiological advantage over cancerous cells [12]. Haghdoost et al. [13] nevertheless found no such correlation between reduction and cytotoxicity or specificity. Rather, reduction was correlated with increased levels of reactive oxygen species (ROS), which might have been the cause of cancer cell death. It suffices to say that reduction is one of the tools for investigating the possible mode of action of catalytic metallodrug coupled with its anticancer activity, and might not address the toxicity and specificity to cancer cells on its own. This is no surprise, as the biological system is heterogeneous, involving several reductive processes, most of which are under strict enzymatic control.

Half-Sandwich complexes such as [(η^6^-arene)M(L)Cl]^+^ have demonstrated good anticancer activity through multimodal binding with DNA [14,15]. Interestingly, one such complex, RAPTA-C, is an antimetastatic, antitumoral and antiangiogenic drug candidate with the potential of reaching clinical evaluation [16]. The properties and anticancer activities of these complexes depend on the ligands coordinated to the metal centre, the fashion they coordinate (monodentate or bidentate), and the structure of the arene [14,17]. This makes half-sandwich complexes potential candidates in anticancer research.

In our previous study [18], we reported a series of N^C Ru(II), Os(II), Rh(III) and Ir(III) half-sandwich derivatives of 1,4-disubstituted 1,2,3-triazole as potential candidates for chemotherapeutic applications. Unfortunately, further studies of these neutral complexes revealed poor selectivity and specificity towards cancer cells. However, the use of cationic complexes could provide selectivity with biomolecules through better solubility, ion exchange or electrostatic interactions. The presence of atoms such as nitrogen may also improve interaction with molecular target through protonation or hydrogen bonding. In this study, efforts were made to achieve selectivity using N^N analogues and further correlate our findings with the ability of the complexes to interact with various biomolecules. The reducing ability of the complexes in a modelled aqueous system was also evaluated. The marked influence of single atom replacement in the ligand, as observed in this work, may provide a key approach to improving the cytotoxicity and controlling the selectivity of this class of compounds to cancer cells.

## 2. Results and Discussion

### 2.1. Synthesis and Characterization of the Ligands and Their Respective Complexes

**Scheme 1:**
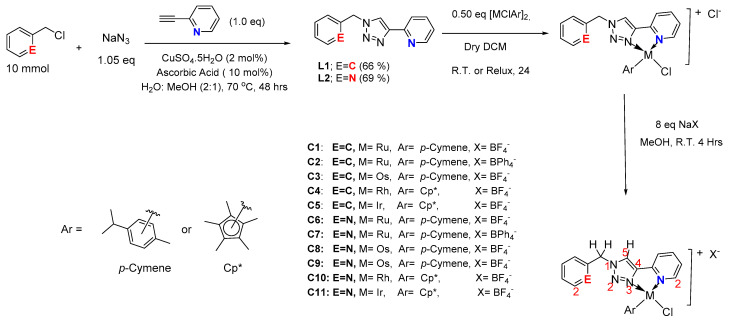
Synthesis of 1,4-disubstituted pyridinyltriazole ligands **L1** and **L2**, and *k*^2^-N^N Ru(II), Os(II), Rh(III) and Ir(III) half-sandwich complexes **C1**–**C11**.

Ligands **L1** and **L2** were prepared via the copper-catalyzed azide-alkyne cycloaddition (CuAAC) reaction between the respective organohalide with 2-ethynyl pyridine and sodium azide in a water–methanol (2:1) mixture under reflux at 70 °C (Scheme 1). After 48 h, the desired products **L1** and **L2** were isolated in good yields of 66% and 69%, respectively, as described in the experimental section.

Complexes **C1**–**C11** were synthesized from ligands **L1** and **L2**, and the metal precursors as summarized in Scheme 1 (Figure S77). These reactions were conducted in air at room temperature for 24 h in the presence of dichloromethane as the solvent. Because of the hygroscopic nature of the formed chloride-containing complexes, chloride counter ion exchange with tetrafluoroborate (BF_4_^−^) and tetraphenylborate (BPh_4_^−^) was considered to give stable and non-hygroscopic complexes. This air stability was achieved by adding stirring excess of the respective sodium salts for 4 h at room temperature, with the stable complexes obtained in good yield. ^1^H, ^1^H−^1^H COSY, ^13^C{^1^H}, ^1^H−^13^C HSQC NMR spectroscopy, HR-MS (ESI) and elemental analysis were used to characterize ligands **L1** and **L2** (Figures S1–S10) and complexes **C1**–**C11** (Figures S11–S65).

All the protons of **L1** shifted downfield upon coordination to Ru(II), Os(II), Rh(III) or Ir(III) metal centres to form complexes **C1**–**C5**. More significant shifts were observed for the triazolyl *H5* and the proton (*H2*) *ortho* to the nitrogen atom on the pyridinyl moiety. There was a marked downfield shift of the proton (*H3*) *meta* to the nitrogen atom of the pyridinyl moiety, which initially overlapped with the ligand’s phenyl protons. The Cp* and the *p*-cymene aromatic protons displayed a downfield shift, with the *p*-cymene aromatic protons in four noticeably different environments due to asymmetry at the metal centre, causing the isopropyl fragment of the *p*-cymene to be diastereotopic. These observations confirmed coordination of the ligand to the metal centres in an N^N bidentate manner involving the gamma–nitrogen (N-3) of the triazole moiety and the pyridinyl nitrogen to afford 5-membered ring half-sandwich complexes **C1**–**C5**.

Although **L2** could coordinate to the metal centre through the pyridinyl nitrogen or the picolyl nitrogen to afford either 5-membered or 6-membered ring complexes, only the 5-membered ring was formed, even under various complexation conditions. There was no trace of the bimetallic complex that could arise from the two possible *N*-coordination sites of **L2**. The second *N*-coordination is limited by increased polarization between N1 and N3 upon coordination of **L2** to the metal centre, through N3 and the pyridine nitrogen, to form the 5-membered ring [19]. This polarization results in partial bond length equalization between the three nitrogen atoms of triazole due to increased back-donation into the triazole π-antibonding (π*)-orbitals [19]. This leads to considerable ring or steric strain in the coordination complex, limiting the formation of a second (6-membered) ring.

Complexes **C8** and **C9** (3:1) are chiral-at-osmium centres, and are therefore isomers with very similar ^1^H NMR and ^13^C{^1^H} NMR chemical shifts, but which differ in colour and solubility. Complex **C9** has a yellow-green colour and is not soluble in DCM, while **C8** is dull green and is soluble in DCM. The isomeric mixture arose in an initial effort to reflux the reaction mixture in DCM and shorten the reaction time. Both **C8** and **C9** show a similar HR-MS (ESI^+^) fragmentation pattern around *m*/*z* = 598.14 for [C_23_H_25_ClN_5_Os]^+^ fragment ion, but **C9** shows an additional fragmentation pattern around *m*/*z* = 417.17 that was not observed with **C8** (Figures S50 and S55 for **C8** and **C9**, respectively). Although all reported complexes studied herein commonly form isomeric mixtures, no further attempt was made to isolate or separate them, as this was outside the scope of this study.

**Figure 1:**
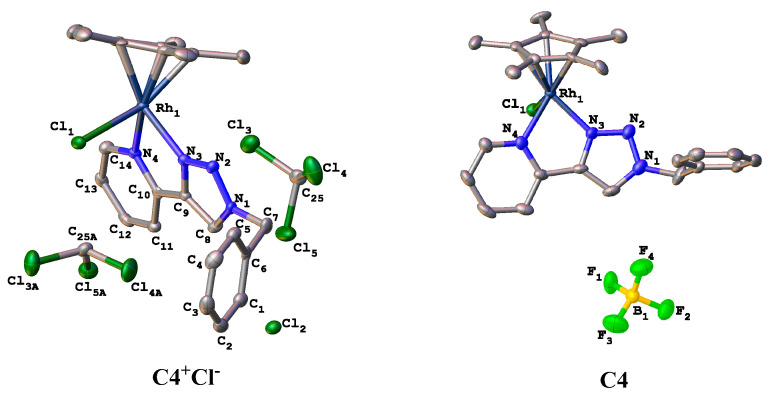
Molecular structures for complex **C4** and its chloride analogue (**C4**^+^Cl^−^).

*Crystal structures of the complexes***:** Single-crystal X-ray crystallography (SCXRD) for complexes **C4** and its chloride analogue confirmed a 5-membered ring half-sandwich complex (Figure 1). Generally, their crystals were red in appearance, adopted a monoclinic crystal system, and assumed a piano-stool or pseudo-octahedral geometry around the Rh metal centre. Complex **C4**, however, crystallized in space group *P*_21/n_, while its Cl^−^ counter ion analogue crystallized in space group C2/c. The different space group adopted by the Cl^−^ counter ion analogue may be due to the incorporation of two chloroform molecules within the crystal lattice that helped stabilize the molecule through hydrogen bonding. In complex **C4**, the phenyl ring is bent towards the triazolyl *N2,* while for its chloride analogue, the phenyl ring is bent towards the triazolyl ring.

The N3-Rh1-N4 bond angles are constrained to 76.19 (**C4^+^Cl**^−^) and 76.9 (**C4**). It appears that the complex with Cl^−^ counterion (**C4^+^Cl**^−^**)** has greater ring strain than that with BF_4_^−^ counterion (**C4**). The bulky counterion provides comparatively more stability to the metal centre by relieving the ring strain. The M-N3 or M-Cl bond length is within range with other Rh(III) triazolyl complexes reported in the literature to have demonstrated good catalytic conversion [20] and cytotoxic activity [18]. Another observation is that the M-N3 bonds are slightly shorter than the M-N4 bonds, evidencing increased backdonation into the triazolyl ring. In general, the complexes are not symmetrical around the metal centres, and therefore, they are chiral at the metal centre.

High Z values are observed for all complexes suggesting a high degree of packing in the solid state. Selected bond lengths and bond angles for complex **C4** and its chloride analogue (**C4^+^Cl**^−^) are reported in Table 1 below, while other crystallographic parameters and data are supplied in Table S1.

### 2.2. NMR Studies on the Stability and Behaviour of the Complexes in an Aqueous Model System

The poor stability of most organometallic compounds after aquation under physiological conditions can hinder their application in catalysis and as anticancer agents [21]. To evaluate this stability and any possibility of interaction with the solubilizing agent DMSO, the stability of complexes **C1**, **C5** and **C11** were probed by ^1^H NMR spectroscopy in a mixture of 10% DMSO-*d*_6_ in phosphate-buffered D_2_O (pH 7.4) at 37 °C. Overall, the best performing complexes when evaluated against cancer cell lines were **C2**, **C7** and **C11**, and were therefore considered for further stability studies. Complexes **C2** and **C7** with BPh_4_^−^ as counterions were poorly soluble in the aqueous model system. Hence, **C1** was selected because of its similarity to the metal centres (Ru). Additionally, complexes **C1**, **C5** and **C11** are representative of the complexes (**C1**, **C3**–**C5**) employed during catalytic evaluation in terms of their structures and metal centres. These NMR studies showed no detectable changes in the ^1^H NMR spectra for over 72 h, suggesting that these complexes are stable under the employed aqueous conditions, implying that DMSO solvent used during anticancer evaluation did not interfere with the treatment or the activities of the complexes.

### 2.3. Catalytic Evaluation

Pyruvate is an essential biomolecule in ATP synthesis in cells. The representative catalytic metallodrugs **C1**, **C3**, **C4** and **C5**, were evaluated for their ability to reduce the pro-chiral biomolecule pyruvate to lactate using sodium formate as the hydride source under a modelled aqueous system of phosphate-buffered saline (PBS), pH 7.4, and temperature of 37 °C. This information was rationalised to establish the mode of action of our metallodrugs and possibly determine the catalytic amount or dose of the metallodrug essential for killing cancer cells before any harm is incurred by normal cells. The catalytic mode of action may potentially minimize toxicity to normal cells and drug resistance.

Table 2 shows the results obtained from the catalytic evaluation of the complexes in the presence of sodium bicarbonate (NaHCO_3_). NaHCO_3_ is the main base in the extracellular environment that helps regulate pH, and has also been used in the treatment of acidosis [22,23]. In all cases in the evaluation, triethylamine (Et_3_N) was employed only for the most active catalyst for comparing the effect of base strength. The **L1** series of complexes with BF_4_^−^ as a counterion (**C1**, **C3**, **C4** and **C5**) were selected as representative complexes.

The **L2** series of complexes (**C6**, **C8**–**C11**) were not used, because they had similar metal centres, with the only differences being a nitrogen atom six bonds away from the metal centres. The nitrogen atom on complexes **C6**, **C8**–**C11** is open to other interactions within the biological system, such protonation, hydrogen bonding, etc and could influence correlation between cytotoxicity and catalytic activity of the complexes. Complexes with BPh4^−^ counterion (**C2** and **C7**) were not considered because of their poor solubility on the employed aqueous solution. Based on the aforementioned points, only the complexes **C1**, **C3**–**C5** were therefore used for the correlation of cytotoxicity results.

**Scheme 2:**
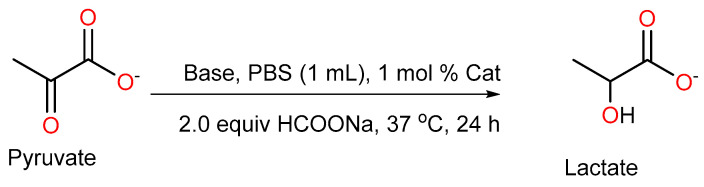
Pseudo-physiological conditions to reduce pyruvate to lactate using phosphate-buffered saline (PBS).

Scheme 2 shows the reaction conditions employed for the transfer hydrogenation of pyruvate to lactate except otherwise stated.

Pyruvate was very soluble in PBS and gave appreciably good conversions for some complexes (Table 2 and Figure S66).

For example, complexes **C1**, **C3** and **C5** gave low conversion (<14%) compared to complex **C4** with excellent conversion (>60%). Additionally, catalytic conversion improved in the absence of a base. The base probably interacts with the cationic complexes, limiting their availability for catalytic reduction.

From Table 2, *p*-cymene derivatives (**C1** and **C3**) were the worst-performing complexes, with conversions of ≤2%. Although **C4** and **C5** are both Cp* derivatives, the partial solubility of **C5** was responsible for the low observed activity in converting pyruvate to lactate. This was confirmed by the remarkable conversions (70 ± 5% versus 14 ± 2% for **C5** and 86 ± 5% for **C4**) of its highly soluble analogue complex **C11,** which varies with only a single atom (nitrogen) away from the metal centre. The addition of a halide abstractor (AgBF_4_) led to exclusive formation (>99%) of the desired lactate product, but with a slight improvement in conversion of the substrate for all the complexes (**C1, C3**–**C5)**. This minor change in conversion suggests that hydrolysis of M-Cl bond is not necessary (i.e., is not the rate-determining step) in catalytic conversion. In addition, other factors such as the inherent ability of the complex to form hydride species might be at play.

It is worth noting that pyruvate dimerizes to give an aldol adduct (Figure S67) in the presence of a base, even without the catalyst. Surprisingly, the addition of a catalyst and base at the onset was observed to suppress this dimerization. Work is underway in our laboratory to determine the mechanism of this suppression.

### 2.4. Cytotoxicity Evaluation

In vitro cytotoxic activities of complexes **C1**–**C11** were also evaluated against Hela (cervical cancer cells), HEK293 (kidney cancer cells), BHK21 (normal kidney cells), MT4 (lymphoma cells) and A549 (lung cancer cells). Auranofin was selected as a positive control since it has been reported to be at least four times more potent than cisplatin and is also powerful against cisplatin-resistant cancer cell lines [24,25]. Cell viability was obtained using the MTS colorimetric assay. The selectivity index (SI) was included to evaluate the effectiveness of synthesized compounds for possible further in vivo anticancer treatment and was limited only to HEK293 and BHK21. The results of these in vitro experiments are summarized in Table 3.

From the results (Table 3), all ligands were inactive (EC_50_ > 200 µM) against all the tested cell lines except in the case of Hela cells, where **L1** showed slight activity (EC_50_ = 168 ± 31 µM). In addition, the metal derivatives were generally not active against A549 and HEK293 except for the **C2**, **C7**, which had BPh_4_^−^ as a counterion. Such improved activity may be due to the increased lipophilicity induced by the BPh_4_^−^ counter ion, which enabled efficient cell membrane crossing by the metallodrug, as previously reported [26,27,28,29]. These two complexes displayed good (EC_50_ < 10 µM) to moderate (EC_50_ < 50 µM) cytotoxic activities against cancer cell lines, but low (EC_50_ between 60 µM and 200 µM) activity against normal cells (BHK21). Complexes **C2** and **C7** showed EC_50_ values of ≤16 µM and ≤53 µM, respectively, against all the selected cancer cells, with SI of 14 and 4, respectively. SI < 2 indicates that a compound is a general toxin and it is usually not considered for follow up as a potential drug candidate [30]. The positive control used (auranofin) was more potent and toxic against all cell lines with a less than one selectivity index. Complex **C2**, with an EC_50_ as low as 9 ± 1 µM and an SI of 14, is a potential candidate for drug evaluation, considering that it is less toxic than auranofin, a drug already in clinical use. These results showed that it is possible to alter a complex’s activity and selectivity to otherwise attack cancer cells that are less susceptible to drugs (A549 and HEK293) by simply changing its counterion. This is further supported by the observed activities between complexes **C1** and **C2** or **C6** and **C7**. For example, complex **C6**, which is a tetrafluoroborate analogue of complex **C7** (with BPh_4_^−^ counter ion), was completely inactive against most cancer cells, whereas **C7** showed good to moderate toxicities against all the cancer cells (EC_50_ < 53 µM and SI of 4).

All complexes obtained from **L2** were found to be non-toxic to the normal kidney cells (BHK21), except for the Ru(II) analogue (**C7**) with BPh_4_^−^ as counterion, which showed low activity with an EC_50_ value of 67 ± 7.3 µM. Similarly, complexes **C1**, **C2,** and **C5** derived from **L1** showed low cytotoxicities against BHK21. Please note that **L1** and **L2** or their respective complexes vary only by a single atom, viz., N or C (Scheme 1). This variation is six bonds away from the metal centre, resulting in altered activity and selectivity. Such a sequential or stepwise ligand variation could provide a useful avenue for combating drug resistance and for addressing their selectivity and toxicity issues.

From the tested cell lines, Hela and MT4 appeared more susceptible to the tested compounds. In comparison, the metal complexes derived from **L1** (**C1**–**C5**) were generally more active than the derivatives of **L2** complexes (**C6**–**C11**) when evaluated against MT4 and Hela. It is worth noting that complexes **C8** and **C9** are isomers, but are not active against any of the tested cell lines. The more significant activity of **L1** (clogP = 2.221) and its derivatives (**C1**–**C5**), when compared to **L2** derivatives, can be attributed to their increased lipophilicities conferred by the benzyl moiety compared to the picolyl group in **L2** (clogP = 0.724), arising from the replacement of N in **L2** by –CH group in **L1**.

Complexes **C5** and **C11** are analogues with variation in a single atom six bonds away from the metal centre: the –CH group for **C5** is replaced with a nitrogen atom in **C11**. However, complex **C5** is toxic to normal cells (55 ± 1 µM), while complex **C11** is completely inactive against normal cells at all concentrations tested. Complex **C11** is the most active of all the complexes reported with an EC_50_ of 2.5 ± 1 µM against Hela, whereas complex **C5** is inactive against Hela (EC_50_ > 500 µM). Additionally, complexes **C5** and **C11** showed varying activity towards MT4. A similar observation was also made for the Os(II) complexes **C3** and **C8**. This again confirms that a single atom variation can also play a role in controlling the activity and selectivity of drugs. These effects of single atom replacement are also noticeable when these results are compared to the reported anticancer activity of analogous N^C derivatives of complexes **C1**–**C5** by Rono et al. [18]. Therefore, varying a single atom adjacent to the metal centre or away from the metal centre controls these triazole-based organometallic compounds’ activities and selectivities. From these results, it is notable that ruthenium(II)-based complexes have superior activity against all the tested cancer cells, followed by the iridium(III) complex.

**Figure 2:**
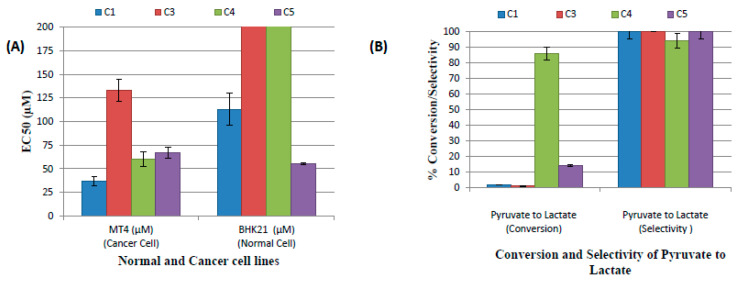
(**A**) Effective concentration (EC_50_) of compounds resulting in 50% cell viability decrease determined using the colorimetric MTS assay for complexes **C1**, **C3**–**C5** (**A**) as evaluated against normal kidney cells (BHK21) and Leukemia cells (MT4), and (**B**) comparative graphs showing the catalytic conversion and selectivity for the transformation of pyruvate to lactate by complexes **C1**, **C3**–**C5**. Error bars represent standard deviation from the mean for n = 6 in the case of cytotoxic evaluation and mean ± SD for n = 3 catalytic runs.

To determine whether the observed activities against cancer could be dependent on their catalytic efficacies in reducing pyruvate to lactate, a comparison of their toxicities against MT4 cells and the results from the catalysis of pyruvate conversion to lactate are provided in Figure 2**.**

From these results, ruthenium(II) complex of ligand **L1** (**C1**), which appeared to be the most active complex, provided inferior conversion of pyruvate to lactate, even in the presence of AgBF_4_. Complex **C4** was highly catalytically active (>80% conversion versus about 2% for **C1**) and resulted in a nearly two-fold decrease in cytotoxic activity compared to **C1**. This implies that the cytotoxicities of these complexes are not dependent on their abilities to reduce pyruvate to lactate.

### 2.5. Interactions of the Complexes with Guanosine-5′-monophosphate (5′-GMP) (DNA Nucleoside)

DNA is one of the primary targets for most, metallodrugs, and their interactions with DNA ahve been linked to their cytotoxicities [31]. To evaluate any possible interaction of the synthesized complexes with DNA, 0.01 mmol of the representative complexes **C1**, **C5** and **C11** was incubated with 2.0 equivalents of 5′-GMP at 37 °C and the respective ^1^H and ^31^P{^1^H} NMR spectra recorded over time. The choice for **C1**, **C5**, and **C11** are representative of **C1**, **C3**–**C5** explored during catalytic evaluation with similar structures and metal centres.

Even though **C2** and **C7** were among the active complexes against cancer cells, their poor solubility in aqueous solution led to the use of complex **C1** (in addition to complexes **C5** and **C11**, as representative complexes) for model biological studies. **C5** and **C11** were included to evaluate the influence of nitrogen atom on the biological activity of the complexes. **C6** was not active, and wasn’t considered for biomolecule studies alongside **C1**.

**Figure 3:**
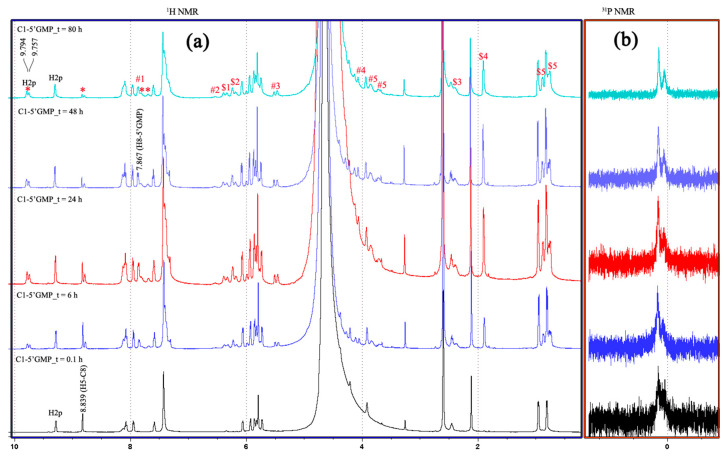
^1^H NMR spectra (column **a**) and ^31^P{1H} NMR spectra (column **b**) for the time-dependent interaction of **C1** and 5′-GMP in 10% DMSO-d_6_-phosphate buffered D_2_O (pH 7.4) over 80 h. Chemical shifts (δ) are reported in parts per million (ppm).

All the representative complexes displayed a time-dependent disappearance of the triazolyl proton *H5* and coordination to 5′-GMP (^1^H NMR of 5′-GMP, Figure S83) through its imidazole N7 nitrogen. The ^31^P NMR shown in all cases helped to probe buffering capacity of the PBS used. While this was stable in most cases, in some, slight loss in buffering capacity could be observed with time as new species formed. The interaction between complex **C1** and 5′-GMP is shown in Figure 3.

From these time-dependent experiments, two significant changes can be observed from the ^1^H NMR spectra. Firstly, there is a noticeable decrease in the intensity of the triazolyl *H5* at 8.83 ppm, perhaps due to deprotonation, but which failed to disappear even after 80 h, probably because of equilibrium between the product species and the substrate in the reaction mixture. Secondly, there is a decrease in the intensity of the benzyl methylene proton of **C1,** suggesting proximity with the triazolyl *H5* proton through space. These observations are in agreement with the carbenylation mechanism proposed by Rono et al. [18] for triazolyl complexes. Additionally, coordination through the imidazole N7 of 5′-GMP can be observed through the appearance of new peaks from both complex **C1** and 5′-GMP (peaks with (*, $ and #) in Figure 3). For the 5′-GMP, the imidazole H-8 proton (proton “1”, Figure S83) shifted upfield from around 8.10 ppm to 7.87 ppm, attributable to the π-π stacking interactions between the nucleobase and the aromatic ligand [32,33,34]. For complex **C1**, there are significant downfield shifts in its peaks. The pyridinyl (H2p) proton close to the metal centre appear at 9.78 ppm, from 9.31 ppm of the free complex **C1**. In addition, the H2p proton and the *p*-cymene CH_3_ peaks appear as two distinct peaks in each case having approximately equal integral values, suggesting two different *N*-coordinated diastereomeric species in the mixture (Figure 3).

**Figure 4:**
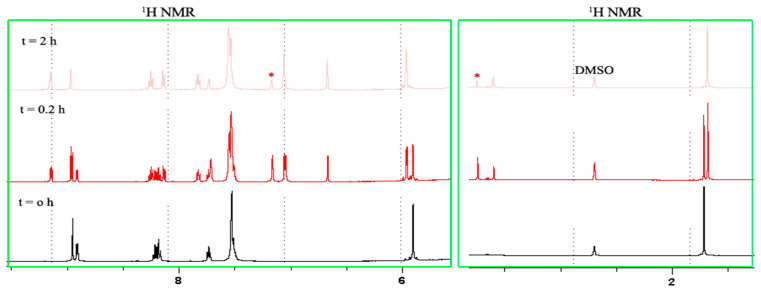
^1^H NMR spectra showing complete coordination of 1-methylimidazole to the chloride analogue of **C5** within 2 h. The peaks with an asterisk (*) are from residual1-methylimidazole.

Similar observations were made for the interaction of complexes **C5** and **C11** with 5′-GMP (Figures S69 and S70, respectively). Additionally, it is notable that for **C5** and **C11,** three different species evident from three peaks for the Cp* moiety are present possibly in equilibrium, namely: starting complex, *k*_1_-N7 coordination species and a carbenylation species, albeit they could not be assigned because of overlap. A repeat of the experiment with other bases such as triethylamine and 1-methylimidazole resulted in deprotonation of the triazolyl H-5 (within 24 h) and *N*-coordination (in 2 h, Figure 4), respectively.

With excess Et_3_N, no *N*-coordination was observed with complexes **C1**, **C5** or **C11**, but complex **C1** appeared to degrade with time, with a decrease in the intensity of its benzyl methylene proton; complex **C5** was very stable even after 21 days, while complex **C11** was stable but its benzyl methylene proton seems almost completely abstracted (Figure S68). These observations seem to be in agreement with catalytic results. Notably, the use of triethylamine caused a significant decrease in conversion and selectivity (Table 2). In addition, the formation of the carbenylation species probably stabilizes the initial complex and hence disfavouring reduction through the metal centre.

**Figure 5:**
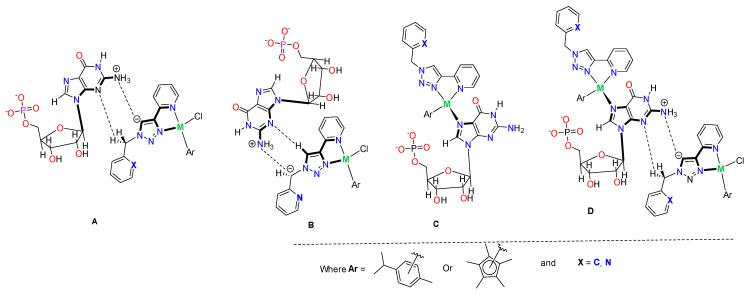
Proposed species (**A**–**D**) formed from the interaction between **C1**, **C5** and **C11**.

Figure 4 reveals that no abstraction of the triazolyl *H5* proton takes place but that *N*-coordination through the metal centre is preferred. These results together with observations made with Et_3_N imply that the imidazole moiety in 5′-GMP will directly coordinate to the metal centre preferentially over base abstraction, while the amino groupwill favor deprotonation of the triazolyl *H5*, as illustrated in Figure 5.

### 2.6. Interaction of the Complexes with Protein and Amino Acids

Bovine serum albumin (BSA) was employed as a suitable model protein because of its homologous nature to human serum album (HSA) [35]. HAS is an abundant protein in blood involved in the transportation of hormones, fatty acids and drugs, among other functions [36]. Interaction of BSA with complexes **C1**, **C5** and **C11** was evaluated by incubating the respective complex with BSA under the pseudo-physiological conditions of a 10% DMSO-*d*_6_ in phosphate-buffered D_2_O (pH 7.4) solution at 37 °C. As shown in Figure S78, there was a time-dependent deprotonation of the triazolyl *H5* proton for complex **C1** and slow coordination for **C5** (red circle for new peaks). All the selected amino acids showed significant interactions and/or coordination with both **C1** and **C5**. L-Histidine (L-His) led to multiple species in solution with *N*-coordination through the imidazole unit (downfield shift of the H-2 of the imidazole to around 7.89ppm) dominating the product mixture (Figure S80). For **C5**_L–His interaction, deprotonation of the triazolyl *H5* occurred first within 1 h of incubation, followed by L–Hist coordination to form at least three new distinct species as the only species after 72 h.

In general, *N*-coordinated L-His diastereomeric adducts and carbenylated species constitute the species observed in the mixture bound to the complexes through the imidazole moiety of L-histidine. Additionally, L-proline (L-pro) formed new species (C1_L_Pro complex) with the complex **C1** (Figure S81), marked with an asterisk (*). Similarly, complex **C5** also interacts with L-Pro much more slowly, even over 96 h (Figure S71).

Similar observations were made on the basis of the interaction of complex **C1** with L-cysteine (L-Cys). The observed species was an L-Cys-**C1** complex, and the cysteine was possibly oxidized to cystine, which precipitated out in the mixture.

For **C5**_L-Cys, (Figure S76), the triazole proton H-5 was intact throughout the experiment. However, two new peaks (#1 and #2, Figure S76) emerged in the aromatic region, and a white precipitate (attributed to cystine) was also observed during the reaction.

## 3. Experimental Section

### 3.1. Materials and Instruments

All reagents used, including sodium azide, 2-(chloromethyl)pyridine hydrochloride, benzyl chloride, 1,2,3,4,5-pentamethylcyclopentadiene, α-phellandrene, ruthenium trichloride hydrate, osmium trichloride, hydrate, rhodium trichloride hydrate, and iridium trichloride hydrate were purchased from Sigma Aldrich Chemical Co. Inc. (St. Louis, MO, USA). NMR solvents were purchased from Sigma Aldrich Chemical Co. Inc., while all other analytical-grade solvents were obtained from Rochelle Chemicals (Johannesburg, South Africa). Dry MeOH was obtained by distillation followed by storage over molecular sieves (3Å, 8–12 mesh), while dry DCM was dispensed from a PureSolv solvent purification system.

Synthesis of all ligands and most complexes was performed in air, and, where needed, air- and moisture-sensitive procedures were carried out using either standard Schlenk line techniques under an argon atmosphere or nitrogen-filled MBraun glovebox. Thin-layer chromatography (TLC) was performed using aluminium TLC plates pre-coated with silica gel 60 F_254_. NMR spectra were recorded using Bruker Avance III, UltrashieledTM 400 or Bruker Avance III HD, AscendTM 500 FT NMR spectrometer. All chemical shifts (δ) were reported in parts per million (ppm) relative to tetramethylsilane (δ = 0.00) as an internal standard. Coupling constants (J values) are given in Hertz. Proton and carbon assignments were confirmed with ^1^H−^1^H COSY, ^1^H−^13^C HSQC and/or ^1^H−^15^N HMBC NMR spectroscopy. Multiplicity is indicated as follows: d = doublet, t = triplet, q = quartet and m = multiplet. The spectra were generated in solutions of CDCl_3_-*d* (δ_H_ 7.24 (s) and δ_C_ 77.0); DMSO-*d*_6_ (δ_H_ 2.49 (s) and δ_C_ 39.5); D_2_O (δ_H_ 4.65(s)) and Acetone-*d*_6_ (δ_H_ 2.85 (d), 2.1 (s). FTIR spectra were recorded on a Perkin Elmer FTIR Spectrometer BX-ATR. Elemental analysis was performed using a Thermo Scientific FLASH 2000 CHNS-O analyzer. Mass spectra were obtained using an HR-MS (ESI) Water Synapt G2 spectrometer. UV-Vis spectra were recorded in DMSO-dH_2_O (1:9) using a Shimadzu UV-1800 spectrophotometer. Single crystals XRD data were obtained using a “Bruker APEX-II CCD” diffractometer.

Any additional methods used, instruments, numerical data and figures are described in the Supplementary Materials.

*Safety precaution*: Although there were no concerns regarding the safety of using organic azides in these experiments, since one-pot synthesis was employed in the syntheses of the ligands, organic azides are known to be explosive. When performing azide experiments, proper personal protective equipment was worn, such as goggles, a face shield, gloves, and a lab coat.

### 3.2. General Procedure for the Ligand Synthesis and Isolation

Synthesis of **L1** and **L2** was straight forward as illustrated in Scheme 1. The reactions were monitored with TLC (5% MeOH, 35% EtOAc, 60% Hexane) for 48 h. To isolate and purify either **L1** or **L2**, the crude mixture was concentrated in vacuo to remove methanol. The resulting product mixture cooled to 4 °C, followed by the addition of 10 mL of a 25% NH_4_OH solution. The desired product was then extracted with DCM and then precipitated in an ice-cold dH_2_O-MeOH mixture.

#### 3.2.1. 2-(1-Benzyl-1H-1,2,3-triazol-4-yl)pyridine (L1)

Light brown white solid; 66% Yield; ^1^H NMR (500 MHz, DMSO-*d*_6_): δ 8.67 (s, “H_e_”, 1H), 8.57 (s, “H_a_”, 1H), 8.02 (s, “H_d_”, 1H), 7.87 (t, ^3^*J*_HH_ = 7.3 Hz, “H_c_”, 1H), 7.33–7.37 (m, “H_h, i, b, g_”, 6H), 5.67 (s, “H_f_”, 2H); ^13^C{^1^H} NMR (125 MHz, DMSO-*d*_6_): δ149.8 (pyridyl tertiary carbon, e), 149.5 (C_a_), 147.4 (benzyl tertiary carbon, C_i_), 137.1 (triazolyl tertiary carbon, C_f_), 135.9 (C_c_), 128.7 (C_k_), 128.1 (C_l_), 127.9 (C_j_), 123.3 (C_g_), 122.9 (C_b_), 119.4 (C_d_), 53.0 (C_h_); λ_max_ = 256.0, 288.5 nm; HR-MS (ESI+): Calculated (*m*/*z*) = 237.1096 [M+H]^+^, Found (*m*/*z*) = 237.1142 [M+H]^+^; Elemental Analysis: Calculated (%): C = 71.17, H = 5.12, N = 23.71. Found (%): C = 71.45, H = 4.89, N = 23.61 (Figures S1–S5).

#### 3.2.2. 2-((4-(Pyridin-2-yl)-1H-1,2,3-triazol-1-yl)methyl)pyridine (L2)

White solid; 69% yield; ^1^H NMR (500 MHz, DMSO-*d*_6_): δ 8.67 (s, “H_f_”, 1H), 8.58 (s, “H_j_”, 1H), 8.53 (s, “H_a_”, 1H), 8.04 (d, ^3^*J*_HH_ = 7.7 Hz, “H_g_”, 1H), 7.88 (t, ^3^*J*_HH_ = 7.5 Hz, “H_h_”, 1H), 7.81 (t, ^3^*J*_HH_ = 7.5 Hz, “H_c_”, 1H), 7.33 (s, “_Hb, d, i_”, 3H), 5.80 (s, “H_e_”, 2H); ^13^C{^1^H} NMR (125 MHz, DMSO-*d*_6_): δ 154.8 (picolyl tertiary carbon, C_e_), 149.9 (pyridyl tertiary carbon, C_j_), 149.5 (C_a_), 149.4 (C_n_), 147.3 (triazolyl tertiary carbon, C_i_), 137.3 (C_c_), 137.1 (C_l_), 124.1 (C_g_), 123.2 (C_m_), 122.9 (C_d_), 122.1(C_b_), 119.4 (C_k_), 54.5 (C_f_); λ_max_ (nm) = 262.0, 277.0; HR-MS (ESI+): Calculated (*m*/*z*) = 238.1048 [M+H]^+^, Found (*m*/*z*) = 238.1094 [M+H]^+^; Elemental Analysis: Calculated (%): C = 65.81, H = 4.67, N = 29.52. Found (%): C = 65.47, H = 4.33, N = 29.18 (Figures S6–S10).

### 3.3. General Procedure for the Synthesis of Complexes C1-C11

The respective ligand (1.0 equiv) and metal dimer (0.5 equiv) were stirred in 10 mL DCM at room temperature and monitored by TLC (5% MeOH:35% EtOAc: 60% Hexane) for 24 h. After 24 h, the respective reaction mixtures were concentrated in vacuo, followed by the addition of MeOH and 8.0 equiv. of NaBF_4_ and then stirred for 4 h. After 4 h, the product mixture was again concentrated in vacuo and then re-dissolved in DCM. The resulting salt that precipitated out was filtered off. The product mixture was again concentrated in vacuo and dried under vacuum overnight. This procedure afforded complexes **C1, C3**–**C5, C6**, **C8**, **C10** and **C11**. For **C2** and **C7**, 8.0 equiv. of NaBPh_4_ was used instead of NaBF_4,_ and the complexes were purified by filtering the resulting precipitate, washing it three times with MeOH and then dried overnight. Upon attempting to reflux **L2** with [OsCl_2_(η^6^-*p*-cym)]_2_ in DCM, a yellowish-green precipitate was formed, together with a dark greenish solution. The precipitate was then filtered, washed with more DCM and counterion exchanged by stirring in MeOH and 8.0 equiv. of NaBF_4_ for 4 h. This afforded **C9**, which was then isolated by concentrating the mixture in vacuo, washing the solid with deionized water to remove excess salt and drying under vacuum overnight. **C8** was obtained from the filtrate as previously described. 

#### 3.3.1. [Chlorido(2-(1-benzyl-(3-κN)-1,2,3-triazol-4-yl)-(2′-κN)-pyridine)(η^6^-p-cymene)ruthenium(II)] tetrafluoroborate (C1)

Orange red solid; 93% yield; ^1^H NMR (500 MHz, DMSO-*d*_6_): δ 9.42 (d, ^3^*J*_HH_ = 4.9 Hz, “H_a_”, 1H), 9.20 (s, “H_e_”, 1H), 8.18–8.20 (m, “H_c_ and H_d_”, 2H), 7.66 (s, “H_b_”, 1H), 7.44 (s, “H_g–i_”, 5H), 6.14 (d, ^3^*J*_HH_ = 5.8 Hz, “H_m_”, 1H), 6.06 (d, ^3^*J*_HH_ = 5.5 Hz, “H_l_”, 1H), 5.98 (d, ^3^*J*_HH_ = 5.5 Hz, “H_l_”, 1H), 5.91 (s, “H_f_”, 2H), 5.85 (d, ^3^*J*_HH_ = 5.8 Hz, “H_m_”, 1H), 2.55–2.58 (m, “H_k_”, 1H), 2.12 (s, “H_n_”, 3H), 1.01 (d, ^3^*J*_HH_ = 6.7 Hz, “H_j_”, 3H), 0.88 (d, ^3^*J*_HH_ = 6.7 Hz, “H_j_”, 3H); ^13^C{^1^H} NMR (125 MHz, DMSO-*d*_6_): δ 155.6 (C_a_), 147.8 (*pyridyl* tertiary carbon, C_e_), 146.2 (benzyl tertiary carbon, C_i_), 140.2 (C_c_), 134.3 (triazolyl tertiary carbon, C_f_), 129.1 (C_j_), 129.0 (C_k_), 128.4 (C_l_), 126.1 (C_b_), 125.6 (C_g_), 122.4 (C_d_), 103.9 (*p*-cymene-CCH(CH_3_)_2_, C_o_), 102.4 (*p*-cymene-CCH_3_, C_r_), 85.7 (*p*-cymene-CHCCH_3_, C_q_), 84.5 (*p*-cymene-(CH_3_)_2_CHCCH, C_p_) 83.6 (*p*-cymene-(CH_3_)_2_CHCCH, C_p_) 83.0 (*p*-cymene-CHCCH_3_, C_q_), 55.3 (C_h_), 30.4 (*p*-cymene-CH(CH_3_)_2_, C_n_), 22.0 *p*-cymene-(CH_3_)_2_, C_m_), 21.1 *p*-cymene-(CH_3_)_2_, C_m_), 18.1 (*p*-cymene-CH_3_, C_s_); λ_max_ = 403.0 nm; HR-MS (ESI^+^): Calculated (*m*/*z*) = 507.0889 [Ru(*p*-cymene)Cl(**L2**)]^+^, Found (*m*/*z*) = 507.0889 [Ru(*p*-cymene)Cl(**L2**)]^+^ (100%); Elemental Analysis: Calculated (%): C = 48.54, H = 4.41, N = 9.44. Found (%): C = 48.58, H = 4.30, N = 9.31 (Figures S11–S15).

#### 3.3.2. [Chlorido(2-(1-benzyl-(3-κN)-1,2,3-triazol-4-yl)-(2′-κN)-pyridine)(η^6^-p-cymene)ruthenium(II)] tetraphenylborate (C2)

Yellow solid; 97% yield; ^1^H NMR (500 MHz, DMSO-*d*_6_): δ 9.43 (d, ^3^*J*_HH_ = 5.2 Hz, “H_a_”, 1H), 9.18 (s, “H_e_”, 1H), 8.16 (d, “H_c_ and H_d_”, 2H), 7.64 (br, d, “H_b_”, 1H), 7.44 (s, “H_g–i_”, 5H), 7.19 (s, “H_o_”, 8H), 6.92 (t, ^3^*J*_HH_ = 7.1 Hz, “H_p_”, 8H), 6.79 (t, ^3^*J*_HH_ = 6.8Hz, “H_q_”, 4H), 6.13 (d, ^3^*J*_HH_ = 5.9 Hz, “H_m_”, 1H), 6.05 (d, ^3^*J*_HH_ = 5.7 Hz, “H_l_”, 1H), 5.96 (d, ^3^*J*_HH_ = 5.7 Hz, “H_l_”, 1H), 5.90 (s, “H_f_”, 2H), 5.84 (d, ^3^*J*_HH_ = 5.7 Hz, “H_m_”, 1H), 2.56–2.58 (m, “H_k_”, 1H), 2.11 (s, “H_n_”, 3H), 1.018 (d, ^3^*J*_HH_ = 6.7 Hz, “H_j_”, 3H), 0.89 (d, ^3^*J*_HH_ = 6.7 Hz, “H_j_”, 3H); ^13^C{^1^H} NMR (125MHz, DMSO-*d*_6_): δ 162.8–163.5 (BPh_4_ tertiary carbon, C_t_), 155.5 (C_a_), 147.7 (*pyridyl* tertiary carbon, C_e_), 146.3 (*benzyl* tertiary carbon, C_i_), 140.1 (C_c_), 135.4 (C_u_), 134.2 (triazolyl tertiary carbon, C_f_), 129.0 (C_j_), 128.9 (C_k_), 128.3 (C_l_), 125.9 (C_b_), 125.5 (C_g_), 125.2–125.3 (C_v_), 122.3 (C_d_), 121.2–121.7 (C_w_), 103.8 (*p*-cymene-CCH(CH_3_)_2_, C_o_), 102.2 (*p*-cymene-CCH_3_, C_r_), 85.6 (*p*-cymene-CHCCH_3_, C_q_), 84.4 (*p*-cymene-(CH_3_)_2_CHCCH, C_p_), 83.5 (*p*-cymene-(CH_3_)_2_CHCCH, C_p_), 82.9 (*p*-cymene-CHCCH_3_, C_q_), 55.2 (C_h_), 30.3 (*p*-cymene-CH(CH_3_)_2_, C_n_), 21.9 (*p*-cymene-(CH_3_)_2_, C_m_), 21.0 (*p*-cymene-(CH_3_)_2_, C_m_), 18.0 (*p*-cymene-CH_3_, C_s_); λ_max_ (nm) = 291.5, 247.5; HR-MS (ESI^+^): Calculated (*m*/*z*) = 507.0889 [Ru(*p*-cymene)Cl(**L2**)]^+^, Found (*m*/*z*) = 507.0888 [Ru(*p*-cymene)Cl(**L2**)]^+^ (100%); HR-MS (ESI^−^): Calculated (*m*/*z*) = 319.1664 [BPh_4_^−^], Found (*m*/*z*) = 319.1677 [BPh_4_]^−^ (100%); Elemental Analysis: Calculated (%): C = 69.78, H = 5.61, N = 6.78. Found (%): C = 69.61, H = 5.48, N =6.67 (Figures S16–S20).

#### 3.3.3. [Chlorido(2-(1-benzyl-(3-κN)-1,2,3-triazol-4-yl)-(2′-κN)-pyridine)(η^6^-p-cymene)osmium(II)] tetrafluoroborate (C3)

Yellow green solid; 83% yield; ^1^H NMR (500 MHz, DMSO-*d*_6_): δ 9.40 (d, ^3^*J*_HH_ = 5.6 Hz, “H_a_”, 1H), 9.27 (s, “H_e_”, 1H), 8.31 (br, t, “H_d_”, 1H), 8.22 (t, ^3^*J*_HH_ = 7.7 Hz, “H_c_”, 1H), 7.62 (t, ^3^*J*_HH_ = 6.4 Hz, “H_b_”, 1H), 7.43–7.44 (m, “H_g–i_”, 5H), 6.37 (d, ^3^*J*_HH_ = 5.5 Hz, “H_m_”, 1H), 6.30 (d, ^3^*J*_HH_ = 5.5 Hz, “H_l_”,1H), 6.19 (d, ^3^*J*_HH_ = 5.5 Hz, “H_l_”, 1H), 6.04 (d, ^3^*J*_HH_ = 5.5 Hz, “H_m_”, 1H), 5.94 (s, “H_f_”, 2H), 2.42–2.46 (m, “H_k_”, 1H), 2.18 (s, “H_n_”, 3H), 0.97 (d, ^3^*J*_HH_ = 6.6 Hz, “H_j_”, 3H), 0.84 (d, ^3^*J*_HH_ = 6.9 Hz, “H_j_”, 3H); ^13^C{^1^H} NMR (125 MHz, DMSO-*d*_6_): δ 155.7 (C_a_), 148.4 (*pyridyl* tertiary carbon, C_e_), 147.4 (*benzyl* tertiary carbon, C_i_), 140.4 (C_c_), 134.2 (triazolyl tertiary carbon, C_f_), 129.0 (C_j_), 128.9 (C_k_), 128.3 (C_l_), 126.7 (C_b_), 126.0 (C_g_), 122.2 (*pyridyl*-CH_d_), 94.7 (*p*-cymene-CCH(CH_3_)_2_, C_o_), 94.3 (*p*-cymene-CCH_3_, C_r_), 77.5 (*p*-cymene-CHCCH_3,_ C_q_), 76.2 (*p*-cymene-(CH_3_)_2_CHCCH, C_p_), 74.5 (*p*-cymene-(CH_3_)_2_CHCCH, C_p_), 73.2 (*p*-cymene-CHCCH_3_, C_q_), 55.2 (C_h_), 30.4 (*p*-cymene-CH(CH_3_)_2_, C_n_), 22.1 (*p*-cymene-(CH_3_)_2_, C_m_), 21.3 (*p*-cymene-(CH_3_)_2_, C_m_), 17.9 (*p*-cymene-CH_3_, C_s_); λ_max_ = 357.0 nm; HR-MS (ESI^+^): Calculated (*m*/*z*) = 597.1461 [Os(*p*-cymene)Cl(**L2**+H)]^+^, Found (*m*/*z*) = 597.1458 [Os(*p*-cymene)Cl(**L2**+H)]^+^ (100%); Elemental Analysis: Calculated (%): C = 42.21, H = 3.84, N = 8.20. Found (%): C = 42.14, H = 3.81, N = 7.98 (Figures S21–S25).

#### 3.3.4. [Chlorido(2-(1-benzyl-(3-κN)-1,2,3-triazol-4-yl)-(2′-κN)-pyridine)(η^6^-pentamethylcypentadiene)rhodium(III)] tetrafluoroborate (C4)

Red solid; 97% yield; ^1^H NMR (500 MHz, DMSO-*d*_6_): δ 9.22 (s, “H_e_”, 1H), 8.87 (s, “H_a_”, 1H), 8.20 (s, “H_c_ and H_d_” 2H), 7.72 (s, “H_b_”, 1H), 7.44 (s, “H_g–i_”, 5H), 5.91 (s, “H_f_”, 2H), 1.67 (s, “H_j_”, 15H); ^13^C{^1^H} NMR (125 MHz, DMSO-*d*_6_): δ 152.12 (C_a_), 147.1 (pyridyl tertiary carbon, C_e_), 146.1 (benzyl tertiary carbon, C_i_), 140.4 (C_c_), 134.3 (triazolyl tertiary carbon, C_f_), 129.1 (C_j_), 129.0 (C_k_), 128.5 (C_l_), 126.9 (C_b_), 125.6 (C_g_), 122.3 (C_d_), 96.8 (Cp* tertiary carbon, C_n_), 55.3 (C_h_), 8.6 (Cp*-(CH_3_)_5_, C_m_); λ_max_ = 369.5 nm; HR-MS (ESI^+^): Calculated (*m*/*z*) = 237.1096 [**L2**+H]^+^, 509.0979 [RhCp*Cl(**L2**)]^+^, 474.1291 [RhCp*(**L2**)]^+^, Found (*m*/*z*) = 237.0647 [**L2**+H]^+^ (100%), 509.0983 [RhCp*Cl(**L2**)]^+^, 474.1248 [RhCp*(**L2**)]^+^; Elemental Analysis: Calculated (%): C = 48.31, H = 4.56, N = 9.39. Found (%): C = 48.23, H = 4.58, N = 9.21 (Figures S26–S30).

#### 3.3.5. [Chlorido(2-(1-benzyl-(3-κN)-1,2,3-triazol-4-yl)-(2′-κN)-pyridine)(η^6^-pentamethylcypentadiene)iridium(III)] tetrafluoroborate (C5)

Dull yellow solid; 93% yield; ^1^H NMR (500 MHz, DMSO-*d*_6_): δ 9.31 (s, “H_e_”, 1H), 8.91 (d, ^3^*J*_HH_ = 5.4 Hz, “H_a_”, 1H), 8.36 (d, ^3^*J*_HH_ = 7.7 Hz, “H_d_”, 1H), 8.25 (t, ^3^*J*_HH_ = 7.4 Hz, “H_c_”, 1H), 7.70 (t, ^3^*J*_HH_ = 6.2 Hz, “H_b_”, 1H), 7.41–7.46 (m, “H_g–i_”, 5H), 5.95 (s, “H_f_”, 2H), 1.68 (s, “H_j_”, 15H); ^13^C{^1^H} NMR (125 MHz, DMSO-*d*_6_): δ 152.1 (C_a_), 147.8 (pyridyl tertiary carbon, C_e_), 147.5 (benzyl tertiary carbon, C_i_), 140.5 (C_c_), 134.1 (triazolyl tertiary carbon, C_f_), 129.0 (C_j_), 128.8 (C_k_), 128.4 (C_k_), 127.2 (C_b_), 126.1 (C_g_), 122.2 (C_d_), 88.7 (Cp* tertiary carbons, C_n_), 55.2 (C_h_), 8.2 (Cp*-(CH_3_)_5_, C_m_); λ_max_ = 327.5 nm; HR-MS (ESI^+^): Calculated (*m*/*z*) = 599.1553 [IrCp*Cl(**L2**)]^+^, Found (*m*/*z*) = 599.1549 [IrCp*Cl(**L2**)]^+^ (100%); Elemental Analysis: Calculated (%): C = 42.02, H = 3.97, N = 8.17. Found (%): C = 41.93, H = 3.82, N = 8.05 (Figures S31–S35).

#### 3.3.6. [Chlorido(2-((4-(pyridine-2-yl)-(3-κN)-1,2,3-trizol-1-yl)methyl)-(2′-κN)-pyridine)(η^6^-p-cymene)ruthenium(II)] tetrafluoroborate (C6)

Brown red solid; 85% yield; ^1^H NMR (500 MHz, DMSO-*d*_6_): δ 9.43 (d, ^3^*J*_HH_ = 5.2 Hz, “H_j_”, 1H), 9.25 (s, “H_f_”, 1H), 8.53 (br, d, “H_a_”, 1H), 8.20–8.22 (m, “H_g_ and H_h_”, 2H), 7.91 (t, ^3^*J*_HH_ = 7.7 Hz, “H_c_”, 1H), 7.66 (br, t, “H_i_”, 1H), 7.55 (d, ^3^*J*_HH_ = 7.7 Hz, “H_d_”, 1H), 7.41 (t, ^3^*J*_HH_ = 5.9 Hz, “H_b_”, 1H), 6.11 (d, ^3^*J*_HH_ = 6.0 Hz, “H_l_”, 1H), 6.04 (s, “H_m_ and H_e_”, 3H), 5.97 (d, ^3^*J*_HH_ = 6.0 Hz, “H_m_”, 1H), 5.83 (d, ^3^*J*_HH_ = 6.0 Hz, “H_l_”, 1H), 2.53–2.57 (m, “H_n_”, 1H), 2.12(s, “H_k_”, 3H), 1.00 (d, ^3^*J*_HH_ = 6.89 Hz, “H_o_”, 3H), 0.89 (d, ^3^*J*_HH_ = 6.89 Hz, “H_o_”, 3H); ^13^C{^1^H} NMR (125 MHz, DMSO-*d*_6_): δ 155.6 (C_m_), 153.2 (picolyl tertiary carbon, C_e_), 149.7 (C_a_), 147.9 (pyridyl tertiary carbon, C_i_), 146.1 (triazolyl tertiary carbon, C_h_), 140.2 (C_k_), 137.7 (C_c_), 126.5 (C_l_), 126.1 (C_g_), 123.9 (C_b_), 122.8 (C_d_), 122.3 (C_j_), 103.9 (*p*-cymene-CCH(CH_3_)_2_, C_o_), 102.5 (*p*-cymene-CCH_3_, C_r_), 85.6 (*p*-cymene-CHCCH_3_, C_p_), 84.6 (*p*-cymene-(CH_3_)_2_CHCCH, C_q_), 83.6 (*p*-cymene-(CH_3_)_2_CHCCH, C_q_), 82.8 (*p*-cymene-CHCCH_3_, C_p_), 56.4 (C_f_), 30.4 (*p*-cymene-CH(CH_3_)_2_, Cs), 21.9 (*p*-cymene-(CH_3_)_2_, C_t_), 21.1 (*p*-cymene-(CH_3_)_2_, C_t_), 18.1 (*p*-cymene-CH_3_, C_n_); λ_max_ (nm) = 259.50, 237.50, 224, 396; HR-MS (ESI^+^): Calculated (*m*/*z*) = 508.0842 [Ru(*p*-cymene)Cl(**L4**)]^+^, Found (*m*/*z*) = 508.0836 [Ru(*p*-cymene)Cl(**L4**)]^+^ (100%); Elemental Analysis: Calculated (%): C = 46.44, H = 4.24, N = 11.77. Found (%): C = 46.23, H = 4.07, N =11.64 (Figures S36–S40).

#### 3.3.7. [Chlorido(2-((4-(pyridine-2-yl)-(3-κN)-1,2,3-trizol-1-yl)methyl)-(2′-κN)-pyridine)(η^6^-p-cymene)ruthenium(II)] tetraphenylborate (C7)

Dull yellow solid; 93% yield; ^1^H NMR (500 MHz, DMSO-*d*_6_): δ 9.43, (s, “H_j_”, 1H), 9.26 (s, “H_f_”, 1H), 8.54 (s, “H_a_”, 1H), 8.19–8.22 (m, “H_g_ and H_h_”, 2H), 7.90 (s, “H_c_”, 1H), 7.66(s, “H_i_”, 1H), 7.55 (br, d, “H_d_”, 1H), 7.18 (s, “H_p_”, 8H), 6.91 (s, “H_q_”, 8H), 6.78 (s, “H_r_”, 4H), 6.103 (s, “H_m_”, 1H), 6.042 (s, “H_e_ and H_m_”, 3H); 5.96 (s, “H_l_”, 1H), 5.83 (s, “H_l_”, 1H), 2.56 (s, “H_n_”, 1H), 2.11 (s, “H_k_”, 3H), 1.01 (br, d, “H_o_”, 3H), 0.90 (br, d, “H_o_”, 3H); ^13^C{^1^H} NMR (125 MHz, DMSO-*d*_6_): δ 163.3–164.1 (BPh_4_ tertiary carbon, C_u_), 156.1 (C_m_), 153.7 (picolyl tertiary carbon, C_e_), 150.14 (C_a_), 148.3 (pyridyl tertiary carbon, C_i_), 146.5 (trizolyl tertiary carbon, C_h_), 140.6 (C_k_), 138.1 (C_c_), 136.0 (C_v_), 127.1 (C_l_), 126.4 (C_g_), 125.7-125.8 (C_w_), 124.3 (C_b_), 123.2 (C_d_), 122.8 (C_j_), 122.1 (C_x_), 104.3 (*p*-cymene-CCH(CH_3_)_2_, C_r_), 102.9 (*p*-cymene-CCH_3_, C_o_), 86.1 (*p*-cymene-CHCCH_3_, C_p_), 85.0 (*p*-cymene-(CH_3_)_2_CHCCH, C_q_), 84.0 (*p*-cymene-(CH_3_)_2_CHCCH, C_q_), 83.2 (*p*-cymene-CHCCH_3_, C_p_), 56.8 (C_f_), 30.8 (*p*-cymene-CH(CH_3_)_2_, C_s_), 22.4 (*p*-cymene-(CH_3_)_2_, C_t_), 21.6 (*p*-cymene-(CH_3_)_2_, C_t_), 18.6 (*p*-cymene-CH_3_, C_n_); λ_max_ (nm) = 239, 290.5, 373.5, 399.5; HR-MS (ESI^+^): Calculated (*m*/*z*) = 508.0842 [M]^+^, Found (*m*/*z*) = 508.0836 [M]^+^ (100%); HR-MS (ESI^−^): Calculated (*m*/*z*) = 319.1664 [BPh_4_]^−^, Found (*m*/*z*) = 319.1672 [BPh_4_]^−^ (100%); Elemental Analysis: Calculated (%): C = 68.24, H = 5.48, N = 8.47. Found (%): C = 67.99, H = 5.51, N = 8.28 (Figures S41–S45).

#### 3.3.8. [Chlorido(2-((4-(pyridine-2-yl)-(3-κN)-1,2,3-trizol-1-yl)methyl)-(2′-κN)-pyridine)(η^6^-p-cymene)osmium(II)] tetrafluoroborate (C8)

Dull green solid; 69% yield; ^1^H NMR (500 MHz, DMSO-*d*_6_): δ 9.40 (d, ^3^*J*_HH_ = 4.9 Hz, “H_j_”, 1H), 9.34 (s, “H_f_”, 1H), 8.54 (br, d, “H_a_”, 1H), 8.38 (d, ^3^*J*_HH_ = 7.7 Hz, “H_g_”, 1H), 8.23 (t, ^3^*J*_HH_ = 7.4 Hz, “H_h_”, 1H), 7.92 (t, ^3^*J*_HH_ = 7.2 Hz, “H_c_”, 1H), 7.63 (t, ^3^*J*_HH_ = 6.1 Hz, “H_i_”, 1H), 7.56 (d, ^3^*J*_HH_ = 7.5 Hz, “H_d_”, 1H), 7.42 (t, ^3^*J*_HH_ = 5.7 ± 0.2 Hz, “H_b_”, 1H), 6.34 (d, ^3^*J*_HH_ = 5.5 Hz, “H_l_”, 1H), 6.29 (d, ^3^*J*_HH_ = 5.2 Hz, “H_m_”, 1H), 6.18 (d, ^3^*J*_HH_ = 5.2 Hz, “H_m_”, 1H), 6.07 (s, “H_e_”, 2H), 6.01 (d, ^3^*J*_HH_ = 5.5 Hz, “H_l_”, 1H), 2.45 (br, s, “H_n_”, 1H), 2.18 (s, “H_k_”, 3H), 0.96 (d, ^3^*J*_HH_ = 6.7 Hz, “H_o_”, 3H), 0.85 (d, ^3^*J*_HH_ = 6.5 Hz, “H_o_”, 3H); ^13^C{^1^H} NMR (125 MHz, DMSO-*d*_6_): δ 155.7 (C_m_), 153.1 (*picolyl* tertiary carbon, C_e_), 149.6 (C_a_), 148.5 (*pyridyl* tertiary carbon, C_i_), 147.2 (triazolyl tertiary carbon, C_h_), 140.4 (C_k_), 137.6 (C_c_), 126.9 (C_g_), 126.6 (C_l_), 123.8 (C_b_), 122.7 (C_d_), 122.1 (C_j_), 94.9 (*p*-cymene-CCH(CH_3_)_2_, C_o_), 94.3 (*p*-cymene-CCH_3_, C_r_), 77.4 (*p*-cymene-CHCCH_3_, C_p_), 76.2 (*p*-cymene-(CH_3_)_2_CHCCH, C_q_), 74.5 (*p*-cymene-(CH_3_)_2_CHCCH, C_q_), 73.0 (*p*-cymene-CHCCH_3_, C_p_), 56.2 (C_f_), 30.4 (*p*-cymene-CH(CH_3_)_2_, C_s_), 22.0 (*p*-cymene-(CH_3_)_2_, C_t_), 21.4 (*p*-cymene-(CH_3_)_2,_ C_t_), 17.9 (*p*-cymene-CH_3_, C_n_); λ_max_ (nm) = 221, 249.5, 264, 352.5; HR-MS (ESI^+^): Calculated (*m*/*z*) = 598.1413 [Os(p-cymene)Cl(**L4**+H)]^+^, Found (*m*/*z*) = 598.1397 [Os(*p*-cymene)Cl(**L4**+H)]^+^ (100%); HR-MS (ESI^−^): Calculated (*m*/*z*) = 87.0035 [BF_4_^−^], Found (*m*/*z*) = 87.0006 [BF_4_]^−^ (100%); Elemental Analysis: Calculated (%): C = 40.39, H = 3.68, N = 10.24. Found (%): C = 40.22, H = 3.52, N = 10.11 (Figures S46–S50).

#### 3.3.9. [Chlorido(2-((4-(pyridine-2-yl)-(3-κN)-1,2,3-trizol-1-yl)methyl)-(2′-κN)-pyridine)(η^6^-p-cymene)osmium(II)] tetrafluoroborate (C9)

Yellow green; 24% yield; ^1^H NMR (500 MHz, DMSO-*d*_6_): δ 9.40 (d, ^3^*J*_HH_ = 5.6 Hz, “H_j_”, 1H), 9.34 (s, “H_f_”, 1H), 8.53 (d, ^3^*J*_HH_ = 4.5 Hz, “H_a_”, 1H), 8.36 (d, ^3^*J*_HH_ = 7.8 Hz, “H_g_”, 1H), 8.23 (t, ^3^*J*_HH_ = 7.7 Hz, “H_h_”, 1H), 7.92 (td, ^3^*J*_HH_ = 7.7 Hz, ^4^*J*_HH_ = 1.6 Hz, “H_c_”, 1H), 7.63 (t, ^3^*J*_HH_ = 6.2 Hz, “H_i_”, 1H), 7.56 (d, ^3^*J*_HH_ = 7.7 Hz, “H_d_”, 1H), 7.41–7.43 (m, “H_b_”, 1H), 6.33 (d, ^3^*J*_HH_ = 5.9 Hz, “H_l_”, 1H), 6.29 (d, ^3^*J*_HH_ = 5.7 Hz, “H_m_”, 1H), 6.18 (d, ^3^*J*_HH_ = 5.7 Hz, “H_m_”, 1H), 6.06 (s, “H_e_”, 2H), 6.01 (d, ^3^*J*_HH_ = 5.9 Hz, “H_l_”, 1H), 2.41–2.45 (m, “H_n_”, 1H), 2.18 (s, “H_k_”, 3H), 0.96 (d, ^3^*J*_HH_ = 6.9 Hz, “H_o_”, 3H), 0.85 (d, ^3^*J*_HH_ = 6.8 Hz, “H_o_”, 3H); ^13^C{^1^H} NMR (125 MHz, DMSO-*d*_6_): δ 156.0 (C_m_), 153.1 (*picolyl* tertiary carbon, C_e_), 149.6 (C_a_), 148.5 (*pyridyl* tertiary carbon, C_i_), 147.2 (triazolyl tertiary carbon, C_h_), 140.6 (C_k_), 137.6 (C_c_), 127.7 (C_g_), 126.9 (C_l_), 123.8 (C_b_), 122.8 (C_d_), 122.4 (C_j_), 94.9 (*p*-cymene-CCH(CH_3_)_2_, C_r_), 94.3 (*p*-cymene-CCH_3_, C_o_), 77.5 (*p*-cymene-CHCCH_3_, C_p_), 76.4 (*p*-cymene-(CH_3_)_2_CHCCH, C_q_), 74.7 (*p*-cymene-(CH_3_)_2_CHCCH, C_q_), 73.1 (*p*-cymene-CHCCH_3_, C_p_), 56.7 (C_f_), 30.4 (*p*-cymene-CH(CH_3_)_2_, C_s_), 22.0 (*p*-cymene-(CH_3_)_2_, C_t_), 21.4 (*p*-cymene-(CH_3_)_2_, C_t_), 18.0 (*p*-cymene-CH_3_, C_n_); λ_max_ (nm) = 221, 249.5, 264, 352.5; HR-MS (ESI^+^): Calculated (*m*/*z*) = 598.1413 [Os(*p*-cymene)Cl(**L4**+H)]^+^, Found (*m*/*z*) = 598.1409 [Os(*p*-cymene)Cl(**L4**+H)]^+^ (100%); HR-MS (ESI^−^): Calculated (*m*/*z*) = 87.0035 [BF_4_^−^], Found (*m*/*z*) = 87.0005 [BF_4_]^−^ (100%); Elemental Analysis: Calculated (%): C = 40.39, H = 3.68, N = 10.24. Found (%): C = 40.45, H = 3.59, N = 9.95 (See Figures S51–S55 for more details).

#### 3.3.10. [Chlorido(2-((4-(pyridine-2-yl)-(3-κN)-1,2,3-trizol-1-yl)methyl)-(2′-κN)-pyridine)(η^6^-pentamethylcypentadiene)rhodium(III)] tetrafluoroborate (C10)

Red solid; 96% yield; ^1^H NMR (500 MHz, DMSO-*d*_6_): δ 9.30 (s, “H_f_”, 1H), 8.90 (d, ^3^*J*_HH_ = 5.5 Hz, “H_j_”, 1H), 8.55 (d, ^3^*J*_HH_ = 4.1 Hz, “H_a_”, 1H), 8.22–8.27 (m, “H_g_ and H_h_”, 2H), 7.92 (td, ^3^*J*_HH_ = 7.7 Hz, ^4^*J*_HH_ = 1.7 Hz, “H_c_”, 1H), 7.72–7.75 (m, “H_i_”, 1H), 7.56 (d, ^3^*J*_HH_ = 7.9 Hz, “H_d_”, 1H), 7.41–7.43 (m, “H_b_”, 1H), 6.06 (s, “H_e_”, 2H), 1.67 (s, “H_k_”, 15H); ^13^C{^1^H} NMR (125 MHz, DMSO-*d*_6_): δ 153.3 (*picolyl* tertiary carbon, C_e_), 152.2 (C_m_), 149.6 (C_a_), 147.1 (*pyridyl* tertiary carbon, C_i_), 145.8 (triazolyl tertiary carbon, C_h_), 140.3 (C_k_), 137.6 (C_c_), 126.8 (C_l_), 126.4 (C_g_), 123.8 (C_b_), 122.8 (C_d_), 122.1 (C_j_), 96.7 (Cp* tertiary carbon, C_o_), 56.2 (C_f_), 8.5 (Cp*-(CH_3_)_5_, C_n_); λ_max_ (nm) = 354, 290,233, 255; HR-MS (ESI^+^): Calculated (*m*/*z*) = 510.0932 [M]^+^, 237.1014 [**L3**]^+^, Found (*m*/*z*) = 510.0922 [M]^+^, 237.5623 [**L3**]^+^; HR-MS (ESI^−^): Calculated (*m*/*z*) = 87.0035 [BF_4_^−^], Found (*m*/*z*) = 87.0008 [BF_4_]^−^ (100%); Elemental Analysis: Calculated (%): C = 46.22, H = 4.39, N = 11.72. Found (%): C = 45.97, H = 4.22, N = 11.81 (Figures S56–S60).

#### 3.3.11. [Chlorido(2-((4-(pyridine-2-yl)-(3-κN)-1,2,3-trizol-1-yl)methyl)-(2′-κN)-pyridine)(η^6^-pentamethylcypentadiene)iridium(III)] tetrafluoroborate (C11)

Shiny yellow solid; 99% yield; ^1^H NMR (500 MHz, DMSO-*d*_6_): δ 9.36 (s, “H_f_”, 1H), 8.92 (d, ^3^*J*_HH_ = 5.5 Hz, “H_j_”, 1H), 8.55 (br, d, “H_a_”, 1H), 8.40 (d, ^3^*J*_HH_ = 7.7 Hz, “H_g_”, 1H), 8.27 (t, ^3^*J*_HH_ = 7.6 Hz, “H_h_”, 1H), 7.92 (t, ^3^*J*_HH_ = 7.7 Hz, “H_c_”, 1H), 7.71 (t, ^3^*J*_HH_ = 6.5 Hz, “H_i_”, 1H), 7.57 (d, ^3^*J*_HH_ = 7.7 Hz, “H_d_”, 1H), 7.42 (br, t, “H_b_”, 1H), 6.08 (s, “H_e_”, 2H), 1.67 (s, “H_k_”, 15H); ^13^C{^1^H} NMR (125 MHz, DMSO-*d_6_*): δ 153.1 (*picolyl* tertiary carbon, C_e_), 152.2 (C_m_), 149.6 (C_a_), 147.8 (*pyridyl* tertiary carbon, C_i_), 147.3 (triazolyl tertiary carbon, C_h_), 140.6 (C_k_), 137.5 (C_c_), 127.3 (C_l_), 127.0 (C_g_), 123.8 (C_b_), 122.8 (C_d_), 122.2 (C_j_), 88.7 (Cp* tertiary carbon, C_o_), 56.3 (C_f_), 8.7 (Cp*-(CH_3_)_5_, C_n_); λ_max_ = 329 nm; HR-MS (ESI^+^): Calculated (*m*/*z*) = 600.1506 [M]^+^, Found (*m*/*z*) = 600.1500 [M]^+^ (100%); Elemental Analysis: Calculated (%): C = 40.21, H = 3.81, N = 10.19. Found (%): C = 40.35, H = 3.94, N = 9.93 (Figures S61–S65).

### 3.4. NMR Studies on the Stability and Behaviour of the Complexes in an Aqueous Model System

A 0.01 mmol of each complex was dissolved in 0.4 mL of a mixture of 10% DMSO-*d*_6_ in phosphate-buffered D_2_O (pH 7.4). Each NMR sample was then incubated at 37 °C, and their respective ^1^H and ^31^P{^1^H} NMR spectra were recorded at 0 h, 24 h, 48 h and 72 h.

### 3.5. Catalytic Evaluation

All transfer hydrogenation reactions were carried out using *Taiatsu Techno* portable steel autoclave reactors PPV-CTR01-CE (Maximum pressure = 10 MPa, Volume = 50 mL) (PPV-CTR01-CE) and fitted into autoclave reactor wells with an inbuilt stirring, heating, and cooling system. In a typical experiment, the substrate (pyruvate), base, and catalyst were added into an autoclave reactor containing a stirring bar. This was followed by adding 1.0 mL PBS (pH 7.4) and lastly by adding sodium formate. In the case where Et_3_N was used, it was added after PBS, followed by sodium formate. The autoclave reactor was then sealed, placed into a reactor well set at 310 K and incubated for 24 h. After 24 h, the autoclave reactor was cooled to 273 K to quench the reaction. An NMR sample, composed of the reaction mixture and DMSO-*d_6_* (1:1.67) and 0.06485 mmol dimethylformamide (DMF) as internal standard, was analyzed, and percentage conversion was obtained. Experiments were done in triplicate and reported as mean ± standard deviation.

### 3.6. Cytotoxicity Evaluation

All ligands (**L1** and **L2**) and synthesized triazolyl conjugated complexes (**C1**–**C11**) were submitted to ADC Mintek, Johannesburg, South Africa, for cell culture and cytotoxicity assays. An MTS (3-(4,5-dimethylthiazol-2-yl)-5-(3-carboxymethoxy-phenyl)-2-(4-sulfophenyl)-2*H*-tetrazolium) in vitro cytotoxicity assay was conducted to determine change in cell viability using a colour change. The MTS compound (yellow) is metabolised by viable cells to form a dark purple coloured compound, visible through UV-Vis spectroscopy at 490 nm. The absorbance is directly proportional to cell viability. Samples were tested across three plates in duplicate (n = 6), and the average value was reported.

HeLa (cervical cancer cells), Hek293 (kidney cancer cells), BHK21 (normal kidney cells), MT4 (lymphoma cells) and A549 (lung cancer cells) were grown using standard tissue culture techniques. The cells (1 × 10^5^ cells/mL) were incubated in 96-well plates at 37 °C overnight, with the subsequent addition of the supplied compounds, at concentrations (100 µg/mL, 50.0 µg/mL, 25.0 µg/mL, 12.5 µg/mL, 6.25 µg/mL, 3.125 µg/mL and 0 µg/mL). The cells were left to incubate for four days, after which MTS (5 µL) was added to the cells. The absorbance values were measured at 490 nm after 1 h, 2 h and 4 h incubation periods, averaged, and the viability curves were drawn up. From the data, the effective concentration (EC_50_) of the compounds resulting in a 50% decrease in cell viability was then determined using a normalized Growth/Sigmoidal dose-response model with OriginPro 8.5 software.

### 3.7. DNA Model Interaction with Guanosine-5′-monophosphate (5′-GMP)

The procedure typically involved the addition of 0.05 mL DMSO-*d*_6_ solution of the complex to 0.45 mL phosphate-buffered D_2_O (pH 7.4) solution of the DNA salt 5′-GMP followed by incubation at 37 °C. The respective ^1^H and ^31^P NMR spectra were then recorded over time. The stoichiometry of the reaction involved 0.01 mmol of the complex with 2.0 equivalents of 5′-GMP.

### 3.8. Protein Interaction

Interaction of bovine serum albumin (BSA) with each complex was evaluated by incubating the respective complex (0.01 mmol) with BSA (30 mg) under the pseudo-physiological conditions of a 10% DMSO-*d*_6_ in phosphate-buffered D_2_O (pH 7.4) solution at 37 °C. The respective ^1^H NMR spectra were then recorded over time.

### 3.9. Amino Acids Interaction with Complexes C1 and C5

Interactions of complexes **C1** and **C5** with the amino acids L-Histidine (L-His), L-proline (L-Pro) and L-cystein (L-Cys) were evaluated by incubating 0.01 mmol of the respective complex in 0.5 mL of 10% DMSO-*d*_6_ in phosphate-buffered D_2_O (pH 7.4) solution at 37 °C with 2.0 equivalents of the respective amino acid. The ^1^H NMR spectra were then recorded over time.

### 3.10. Crystal Structure Determination for C4 and Its Cl^−^ Counterion Analogue

Suitable crystals for complex **C4** were obtained through slow evaporation of its dichloromethane-diethyl ether solution, whereas a chloroform-diethyl ether solvent mixture was used for its chloride analogue.

### 3.11. Determining Calculated Partition Coefficient (clogP)

The logP value of a compound is the logarithmic value for its partion between 2 solvents notably octanol and water (log(c_octanol_/c_water_)). The calculated logP (clogP) values were estimated using ChemDraw 2019.

## 4. Conclusions

In conclusion, eleven *k*^2^-N^N complexes (**C1**–**C11**) of Ru(II), Os(II), Rh(III) and Ir(III) based on 1-benzyl-4-pyridinyl-1*H*-1,2,3-triazole (**L1**) and 1-picolyl-4-pyridinyl-1*H*-1,2,3-triazole (**L2**) were prepared and characterized successfully. Their cytotoxic activities were evaluated against Hela (cervical cancer cells), HEK293 (kidney cancer cells), MT4 (lymphoma cells), A549 (lung cancer cells) and BHK21 (normal kidney cells). Although many of these complexes were not active, complexes **C2**, **C7** and **C11** showed moderate to good toxicities against all the tested cancer cells and excellent selectivity to cancer cells with SI > 10 for **C2**. At the same time, **C11** is non-toxic to normal cells and to the tested cancer cells, except Hela cells. Some of the factors influencing the observed bioactivities include the counterion used, the lipophilicities, the nature of the ligand, and the metal used.

Whereas the complexes showed activity in catalytic transfer reduction of pyruvate under physiological conditions, a comparison between catalytic conversions and EC_50_ values of the complexes did not show any direct correlation. Therefore, these results imply that the complexes’ catalytic reductive abilities may influence the inherent activity of the complex, but not their primary mode of exerting their cytotoxic activities. Clearly, the strong interactions of the complexes with biomolecules through coordination to the metal centre might provide these complexes’ critical modes of action, as evidenced by time series NMR spectroscopy results of the interactions between select complexes and model biomolecules such as BSA, 5′-GMP and amino acids.

## Figures and Tables

**Table 1:** Selected bond lengths and bond angles for complex **C4** and related chloride analogue (**C4**^+^**Cl**^−^).

Complex	Atoms	Bond Distance (Å)	Atoms Involved	Bond Angle (°)
**C4^+^Cl^−^**	Rh1-N3	2.115 (3)	N2-N3-Rh1	133.6 (3)
	Rh1-N4	2.135 (5)	C9-N3-Rh1	115.4 (3)
	Rh1-Cl	2.3871 (11)	C10-N4-Rh1	117.1 (3)
	N3-**C9**	1.364 (5)	N3-N2-N1	105.1 (3)
	N4-**C10**	1.358 (5)	N4-C10-C9	113.7 (3)
	N1-**C8**	1.338 (5)	C10-C9-N3	117.7 (3)
	N1-**C7**	1.474 (5)	N3-Rh1-N4	76.19 (13)
**C4**	Rh1-N3	2.085 (5)	N2-N3-Rh1	133.9 (4)
	Rh1-N4	2.129 (5)	C9-N3-Rh1	115.3 (4)
	Rh1-Cl	2.384 (2)	C10-N4-Rh1	115.6 (4)
	N3-**C9**	1.363 (8)	N3-N2-N1	104.9 (5)
	N4-**C10**	1.354 (8)	N4-C10-C9	113.9 (6)
	N1-**C8**	1.344 (9)	C10-C9-N3	117.2 (6)
	N1-**C7**	1.448 (9)	N3-Rh1-N4	76.9 (2)

**Table 2:** Results for the catalytic reduction of pyruvate to lactate under pseudo-physiological conditions *^a^*.

Catalyst	Base	Conversion (%)	TON	Selectivity (%)	Structures of the Complexes
**No catalyst**	None	6 ± 1	6	0	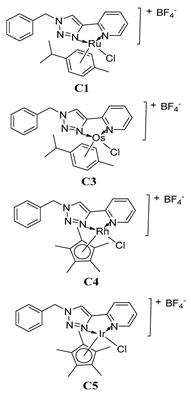
**No catalyst**	NaHCO_3_	52 ± 2	52	0	
**C1**	None	2 ± 0.2	2	100	
**C1**	NaHCO_3_	0.1 ± 0.02	≤0.1	100	
C1 *	None	5 ± 1	5	>99	
**C3**	None	1 ± 0.3	1	100	
**C3**	NaHCO_3_	0.1 ± 0.03	≤0.1	100	
C3 *	None	3 ± 1	3	>99	
**C4**	None	86 ± 5	86	94	
**C4**	NaHCO_3_	70 ± 2	70	80	
**C4**	Et_3_N	60 ± 4	60	44	
C4 *	None	90 ± 5	90	>99	
**C5**	None	14 ± 2	14	100	
**C5**	NaHCO_3_	0.1 ± 0.02	≤0.1	100	
C5 *	None	20 ± 3	20	>99	

*^a^* Catalytic reduction of sodium pyruvate (0.445 mmol) over 24 h at 310 K using 1.0 equiv. base, 2.0 equiv. sodium formate, 1 mol% catalyst with PBS (pH 7.4, 1mL) as solvent. Turn over number (TON) and percentage selectivity were determined using ^1^H NMR spectroscopy with dimethylformamide (DMF) as an internal standard (Figure S66). TON represents the total number of substrate molecules that are converted into lactate and percentage selectivity represents the percentage of the lactate formed. * 2.0 equiv. of the halide abstractor AgBF_4_ was added as a reagent.

**Table 3:** Effective concentration (EC_50_) of compounds against normal kidney cells (BHK21), lung cancer cells (A549), kidney cancer cells (HEK293), cervical cancer cells (Hela), and Lymphoma cells (MT4).

Compounds	EC_50_ (µM)								
	A549	SI	HEK293	SI	Hela	SI	MT4	SI	BHK21
**L1**	>1300	n/d	>1300	≤1	168 ± 31	n/d	>1300	n/d	>1300
**C1**	>500	n/d	>500	<1	>500	n/d	37 ± 5	n/d	113 ± 17
**C2**	14 ± 4.0	n/d	11 ± 1.5	14	15 ± 1.7	n/d	9 ± 1	n/d	151 ± 0.6
**C3**	>500	n/d	>500	≤1	>500	n/d	133 ± 12	n/d	>500
**C4**	>500	n/d	>500	≤1	≥300	n/d	60 ± 8	n/d	>500
**C5**	>500	n/d	>500	<1	>500	n/d	67 ± 6	n/d	55 ± 1
**L2**	>1300	n/d	>1300	≤1	1070 ± 134	n/d	N.R.	n/d	>1300
**C6**	N.R.	n/d	>500	-	388 ± 70.9	n/d	158 ± 44.2	n/d	N.R.
**C7**	53 ± 6.0	n/d	17 ± 4.8	4	26 ± 0.8	n/d	20 ± 4.8	n/d	67 ± 7.3
**C8**	>500	n/d	>500	≤1	N.R.	n/d	N.R.	n/d	>500
**C9**	>500	n/d	>400	-	N.R.	n/d	N.R.	n/d	N.R.
**C10**	N.R.	n/d	>500	-	N.R.	n/d	>500	n/d	N.R.
**C11**	>500	n/d	>500	-	2.5 ± 1	n/d	111 ± 7	n/d	N.R.
Auranofin	12 ± 2	n/d	<0.1	≤1	<2	n/d	<2	n/d	<0.1

EC_50_ is the effective concentration resulting in 50% decrease in cell viability determined from the calorimetric MTS assay and calculated using a normalized sigmoidal dose response curve as evaluated. Exposure time of the cells to the treatment compounds was 96 h; thereafter, average absorbance values were obtained by MTS assay. Each value represents mean ± SD across 3 plates, in duplicates (n = 6) measured at 490 nm over 1 h, 2 h and 4 h intervals. Compounds for which no SI is provided are compounds that are completely inactive against both normal cells (BHK21) and cancer cells (A549, HEK293, Hela, MT4). N.R. denotes no response, implying complete inactivity at tested concentrations. n/d denotes “not determined”. SI equals effective concentration (EC_50_) against normal Kidney cells (BHK21) divided by EC_50_ for the compound against kidney cancer cells (HEK293).

## Data Availability

The data used in this study are incorporated into the article and are freely available online as Support Information.

## Supplementary Materials

The following supporting information can be downloaded at: https://www.mdpi.com/article/10.3390/molecules27072058/s1, Figures S1–S83; Table S1: Crystallographic parameters and data for complexes **C4** and its chloride counter ion analogue (**C4^+^Cl^−^**).

## Sample Availability

Samples of all the compounds are available from the authors.
