# Supplementary material for: New Triazolyl N^N Bidentate Rh(III), Ir(III), Ru(II) and Os(II) Complexes: Synthesis and Characterization, Probing Possible Relations between Cytotoxicity with Transfer Hydrogenation Efficacy and Interaction with Model Biomolecules
Source: Molecules. 2022 Mar 23;27(7):2058. doi: 10.3390/molecules27072058 (PMC9000499; doi:10.3390/molecules27072058)
Supplement: Supplementary file 1 [file molecules-27-02058-s001.zip › molecules-1597175-supplementary.pdf]

New Triazolyl N^N bidentate Rh(III), Ir(III), Ru(II) and Os(II) complexes: synthesis, characterization, probing possible relations between cytotoxicity with transfer hydrogenation efficacy and interaction with model biomolecules

William K. Chu,[a] Charles K. Rono[a] and Banothile C.E. Makhubela[a*]

[a] *Research Centre for Synthesis and Catalysis, Department of Chemical Sciences, University of Johannesburg, Auckland Park Campus,2006, Johannesburg, South Africa,*

**Corresponding author E-mail:* bmakhubela@uj.ac.za

Keywords: Transfer hydrogenation, Cancer, Click Chemistry, Catalysis, Chemotherapy, Triazole.

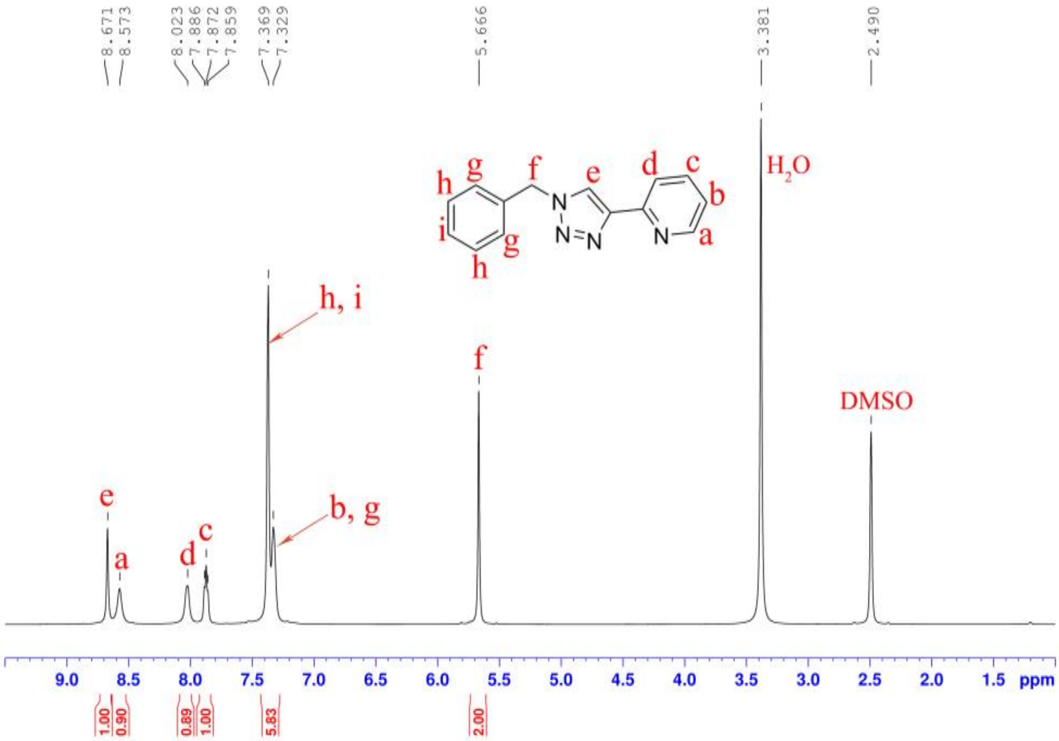

Figure S1: ^{1}H NMR spectrum of **L1** obtained using DMSO-d_6.

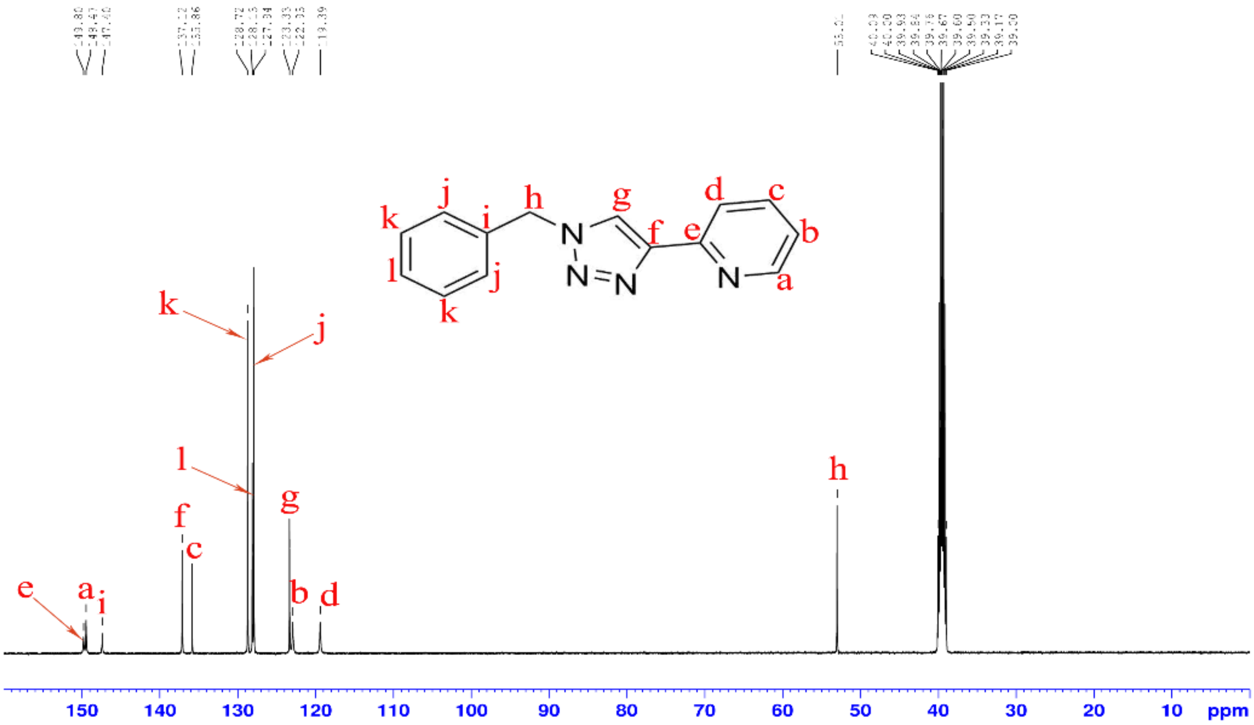

Figure S2: $^{13}C\{^1H\}$ NMR spectrum of **L1** in DMSO-d_6.

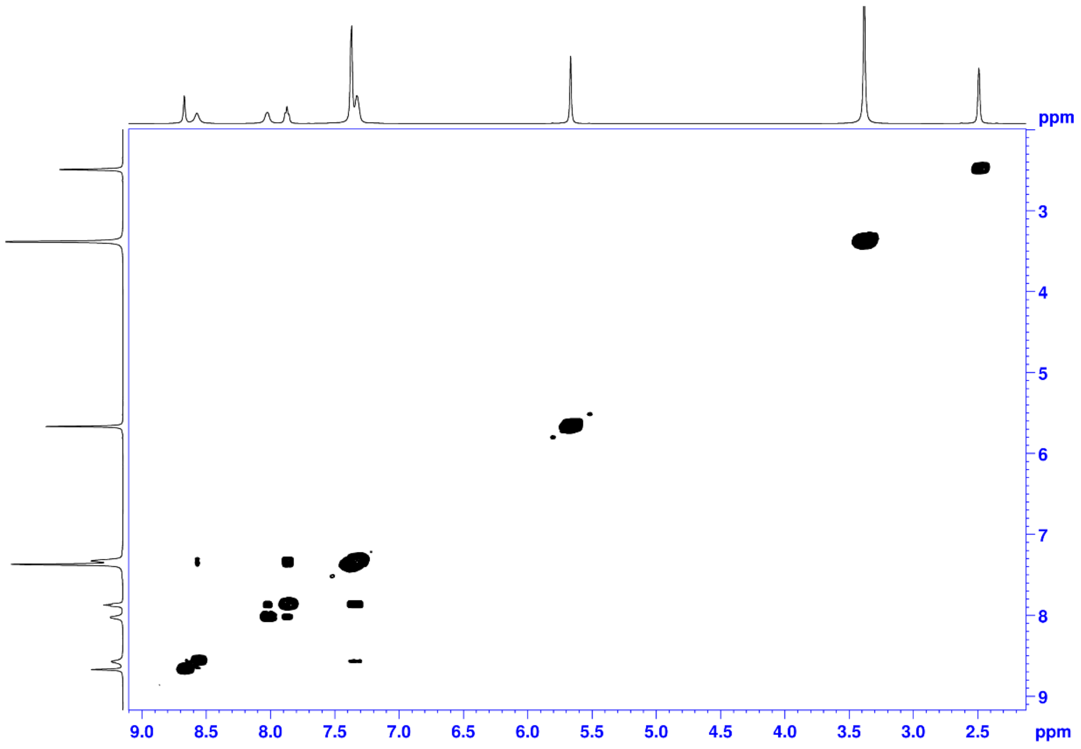

Figure S3: ^{1}H–^{1}H COSY NMR spectrum of **L1** in DMSO-d_6.

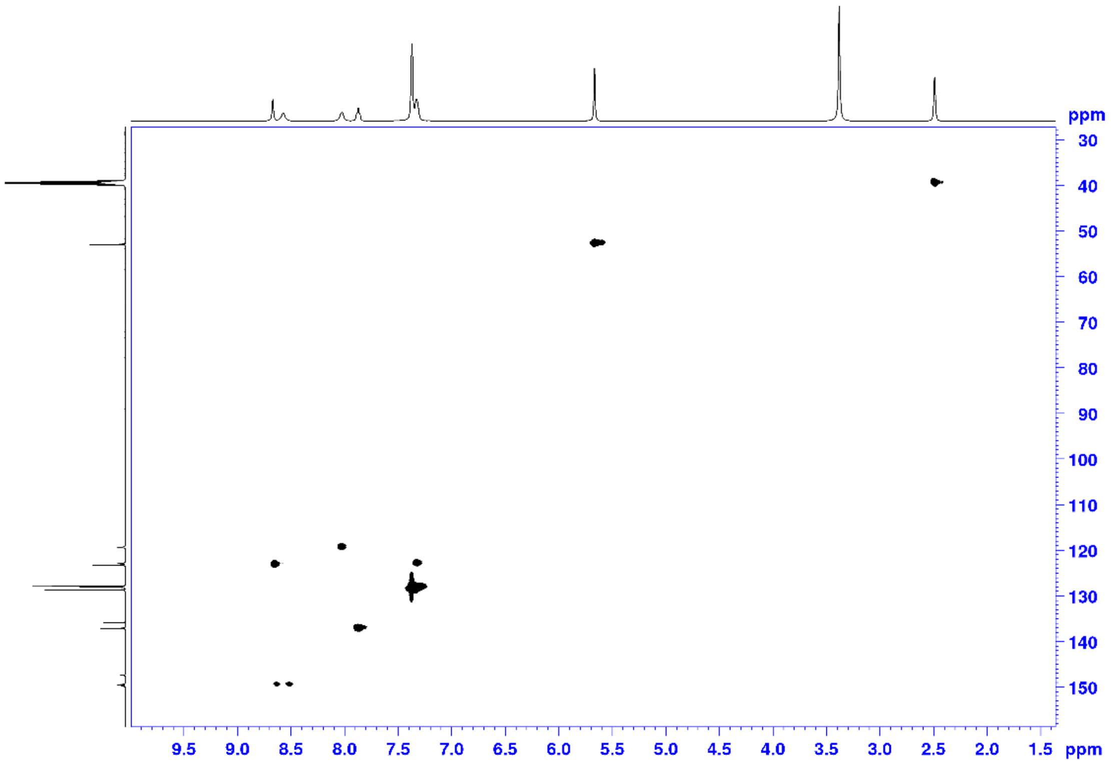

Figure S4: ^{1}H–^{13}C HSQC NMR spectrum of **L1** in DMSO-d_6.

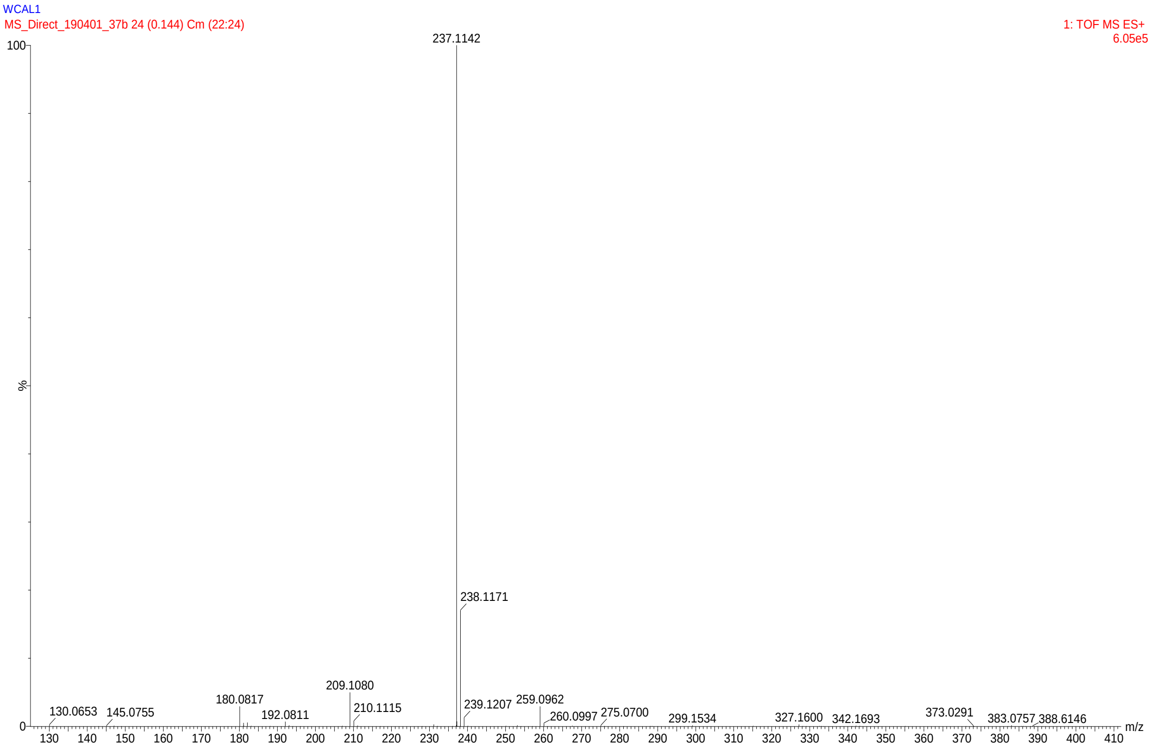

Figure S5: HRMS (ESI^+) of triazole **L1.**

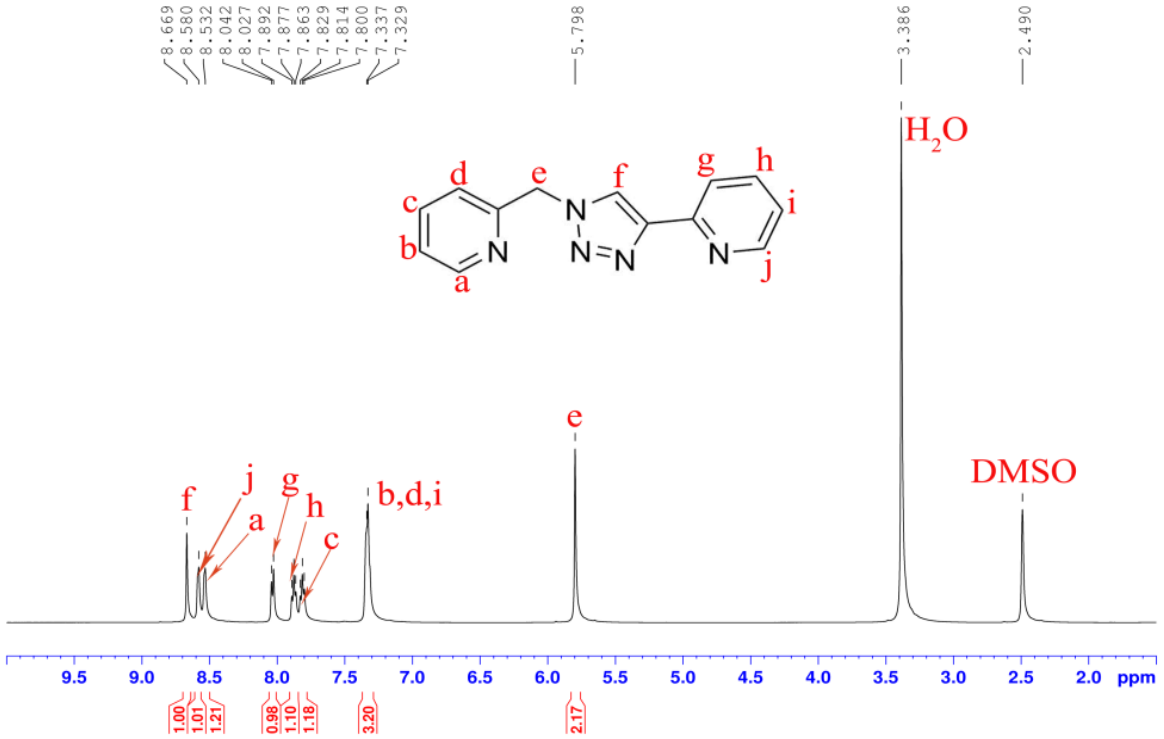

Figure S6: ^{1}H NMR spectrum of triazole **L2** in obtained in DMSO-d_6.

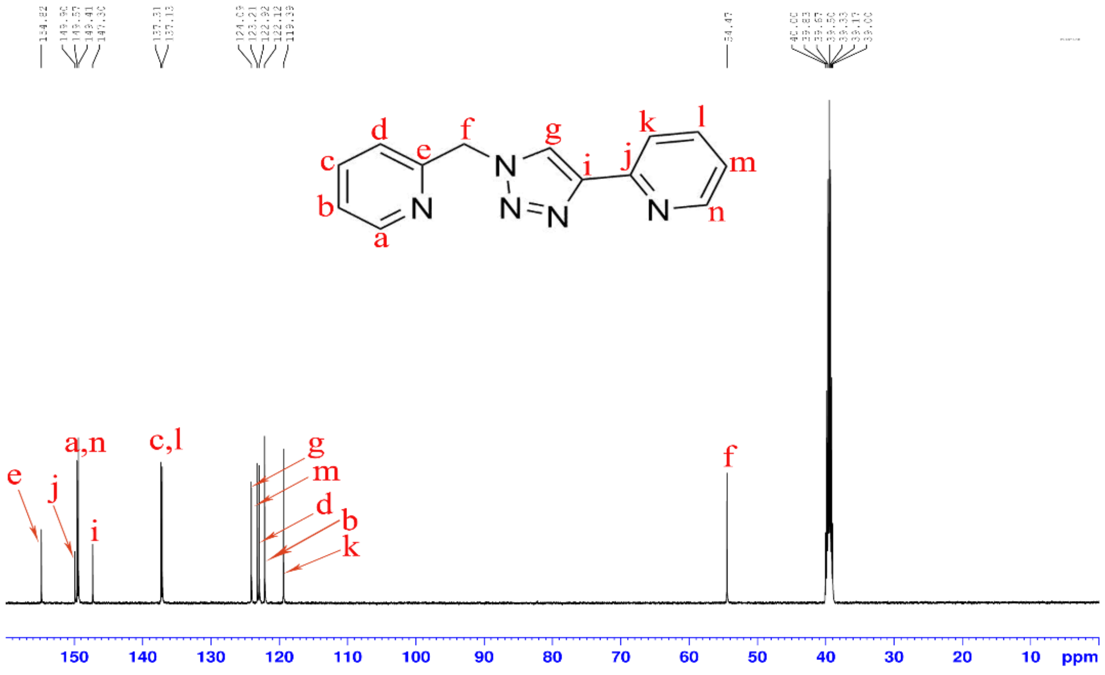

Figure S7: $^{13}C\{^{1}H\}$ NMR spectrum of triazole **L2** in DMSO-d_6.

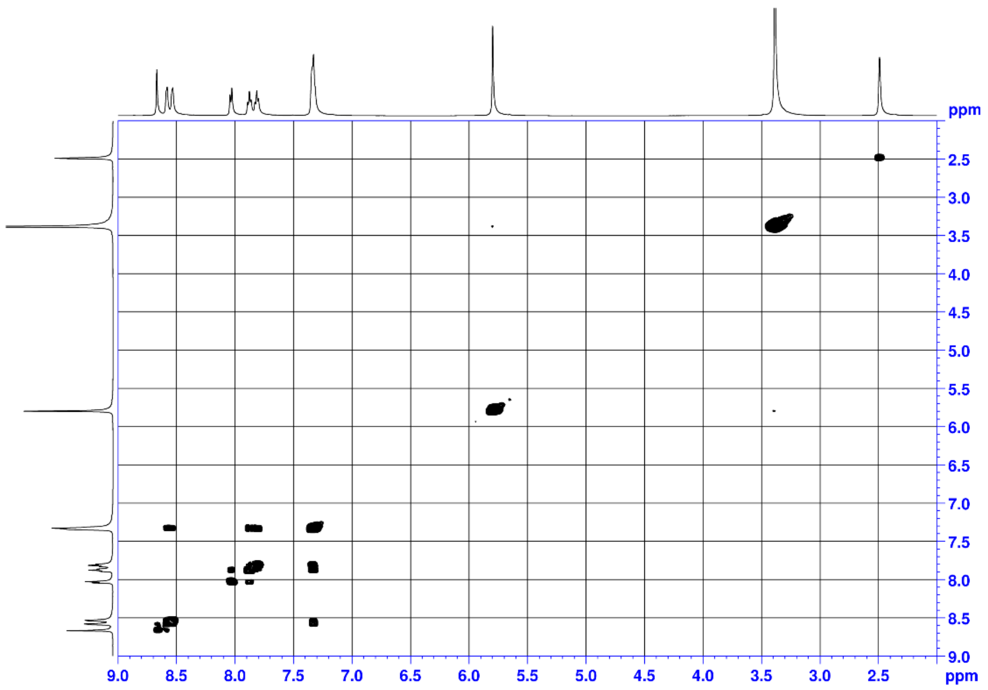

Figure S8: ^{1}H–^{1}H COSY NMR spectrum of triazole **L2** in DMSO-d_6.

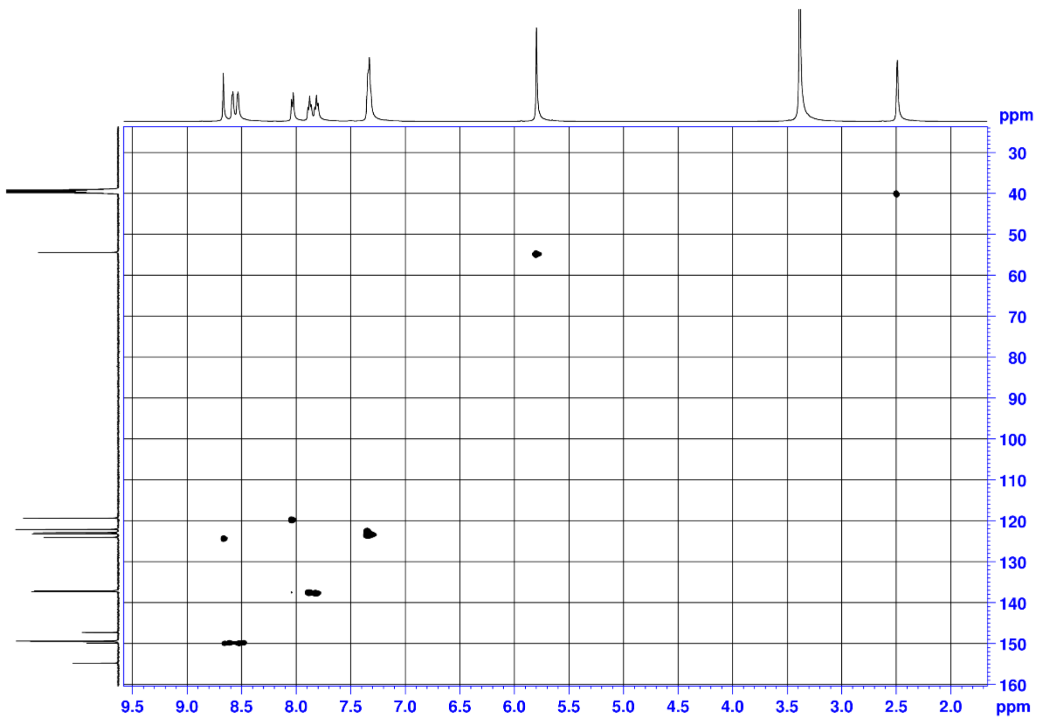

Figure S9: ^{1}H–^{13}C HSQC NMR spectrum of triazole **L2** in DMSO-d_6.

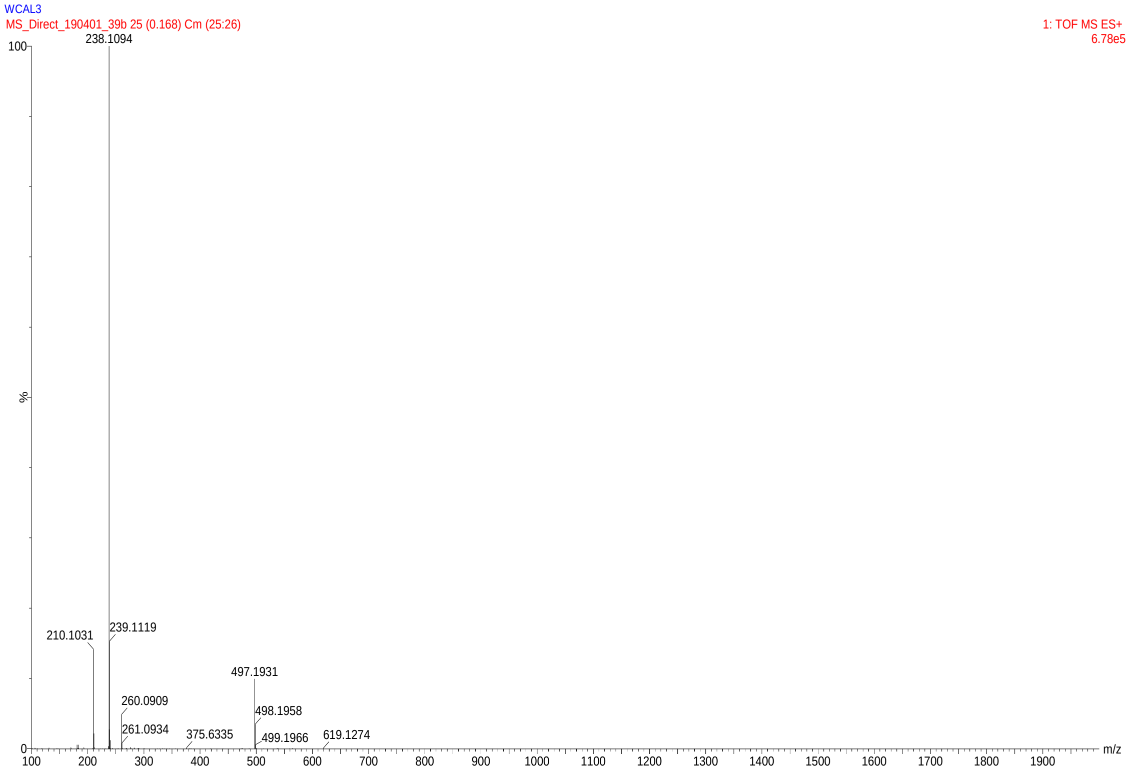

Figure S10: HRMS (ESI^{+}) of triazole **L2.**

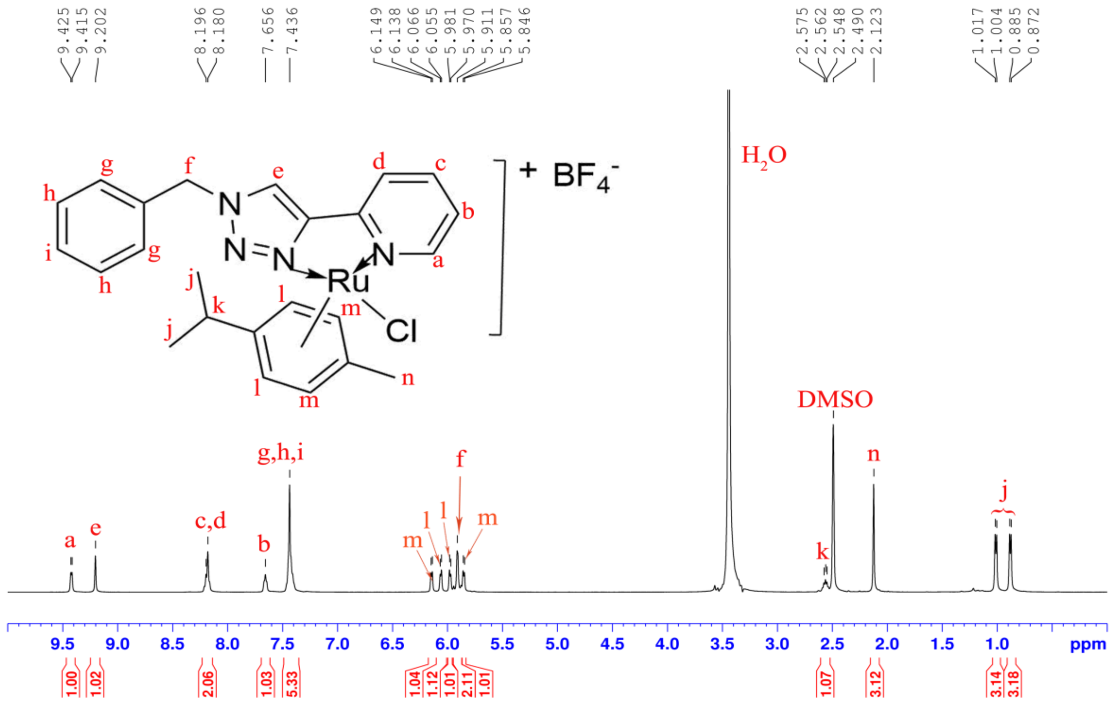

Figure S11: ^{1}H NMR spectrum of complex **C1** in DMSO-d_6.

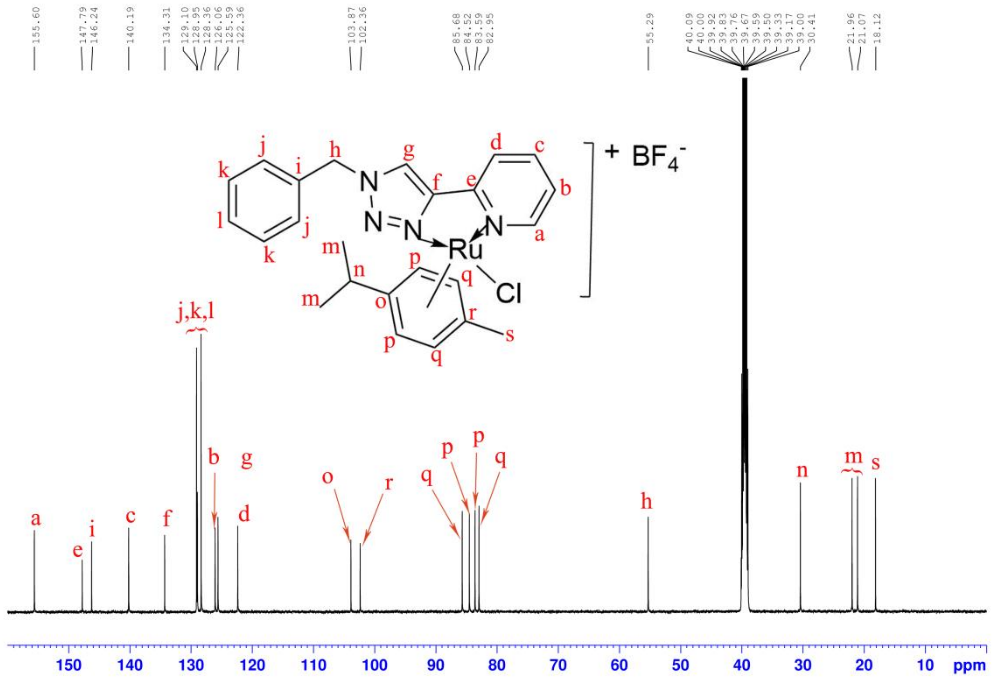

Figure S12: ^{13}C{^{1}H} NMR spectrum of complex **C1** in DMSO-d_6.

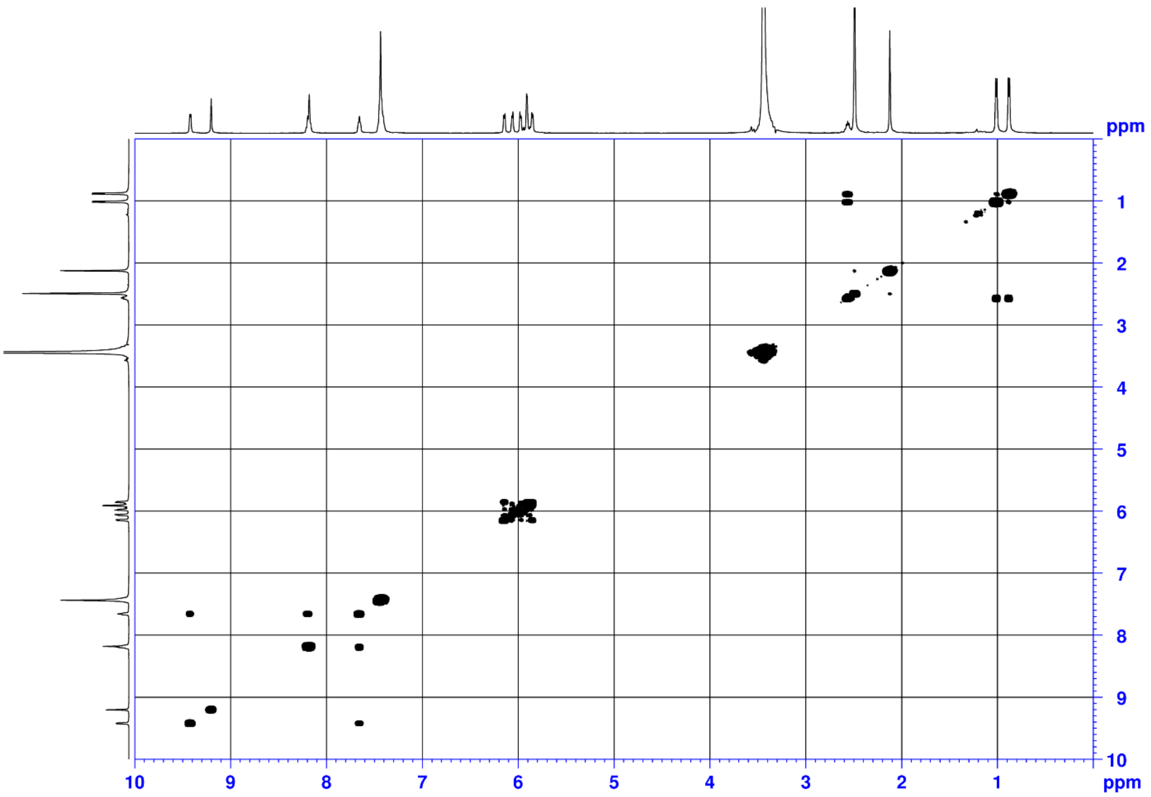

Figure S13: ^{1}H–^{1}H COSY NMR spectrum of complex **C1** in DMSO-d_6.

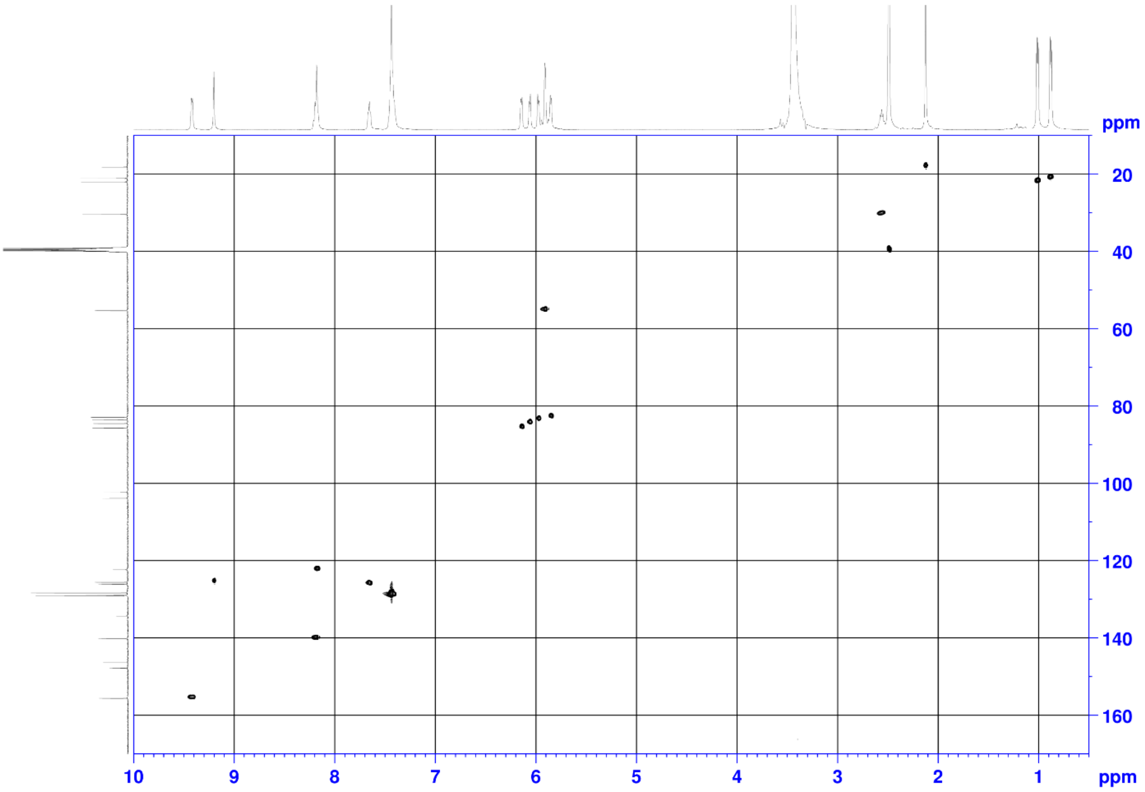

Figure S14: ^{1}H–^{13}C HSQC NMR spectrum of complex **C1** in DMSO-d_6.

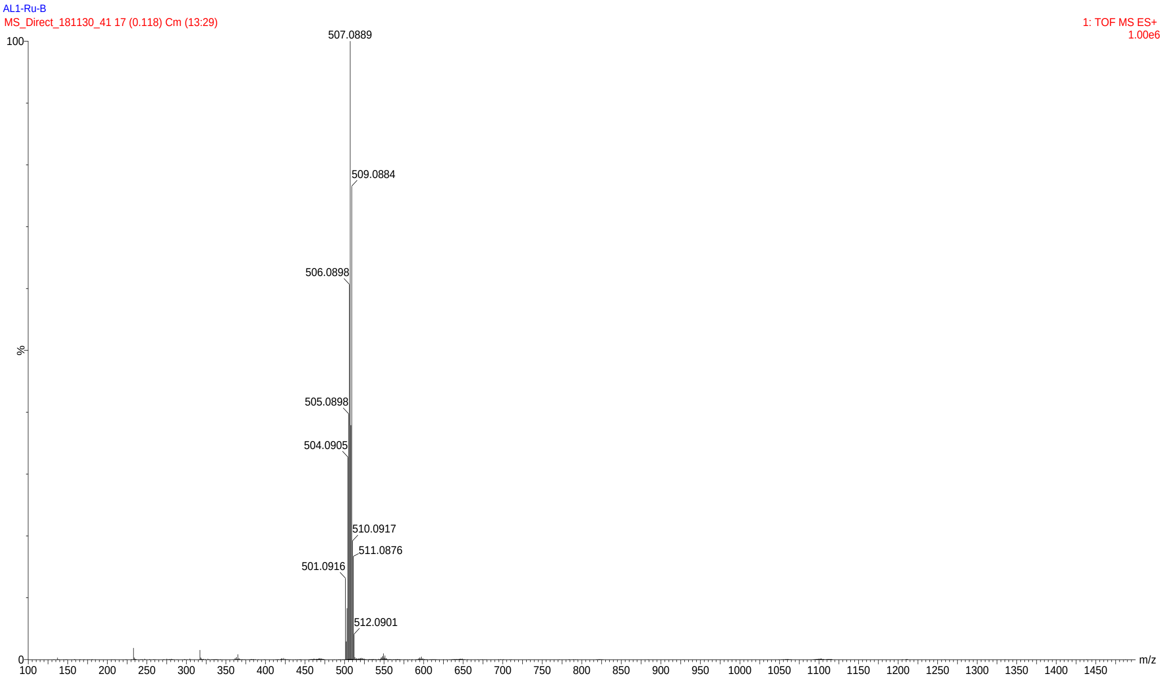

Figure S15: HRMS (ESI$^+$) of complex **C1.**

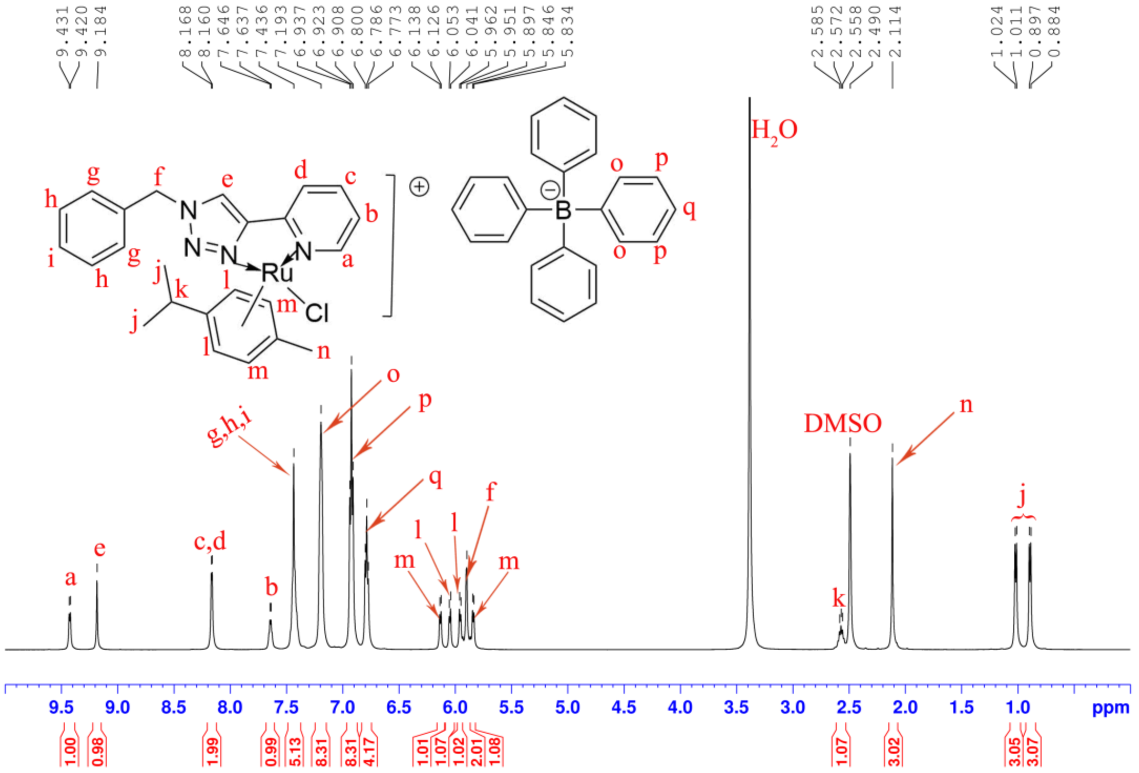

Figure S16: ^{1}H NMR spectrum of complex **C2** in DMSO-d_6.

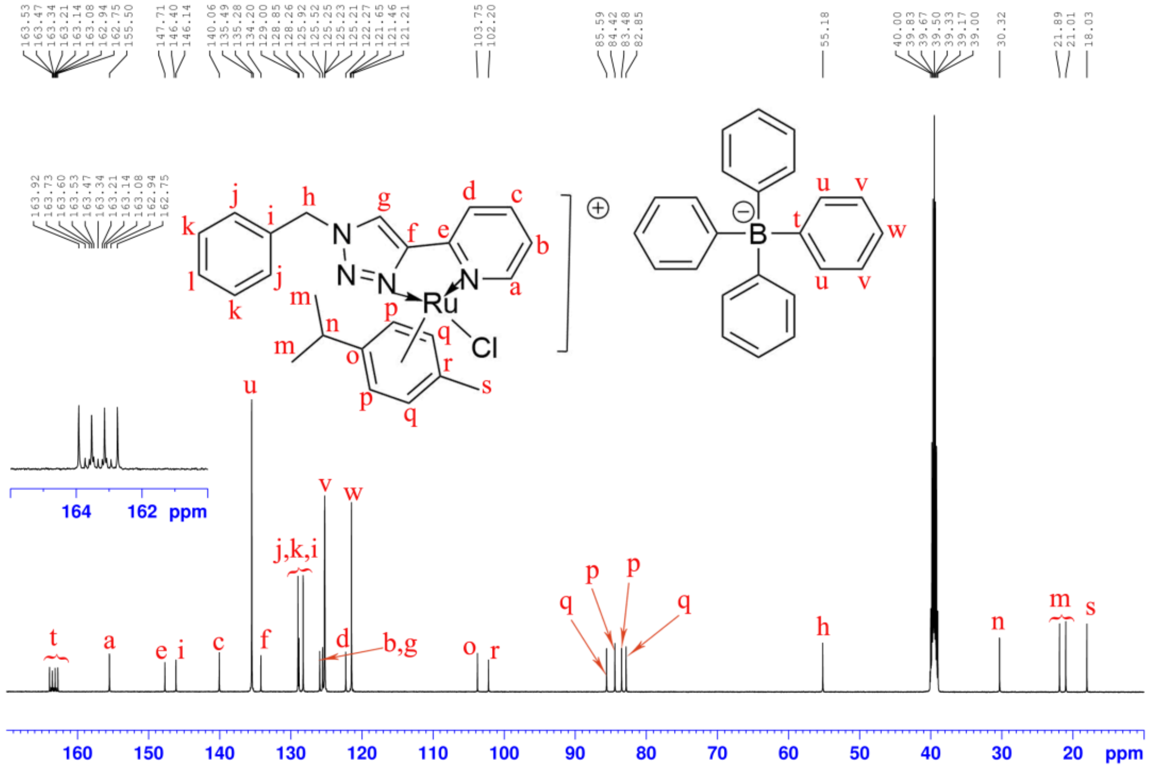

Figure S17: $^{13}C\{^{1}H\}$ NMR spectrum of complex **C2** in DMSO-d_6.

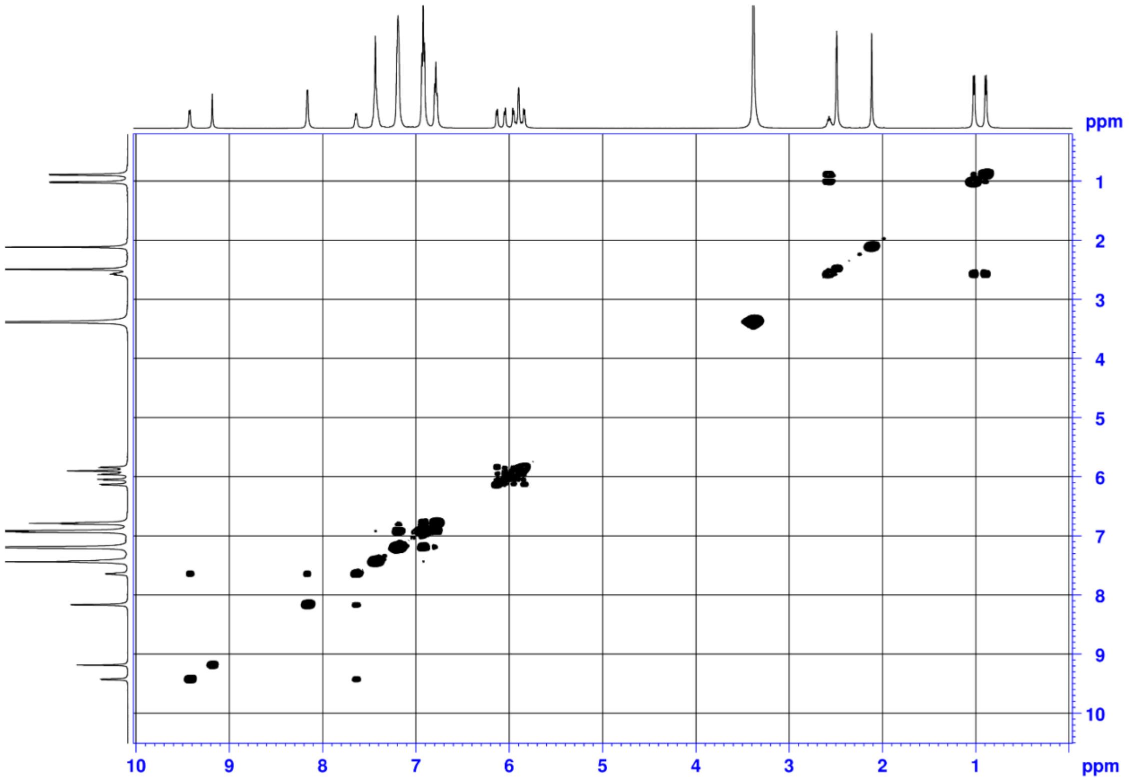

Figure S18: ^{1}H–^{1}H COSY NMR spectrum of complex **C2** in DMSO-d_6.

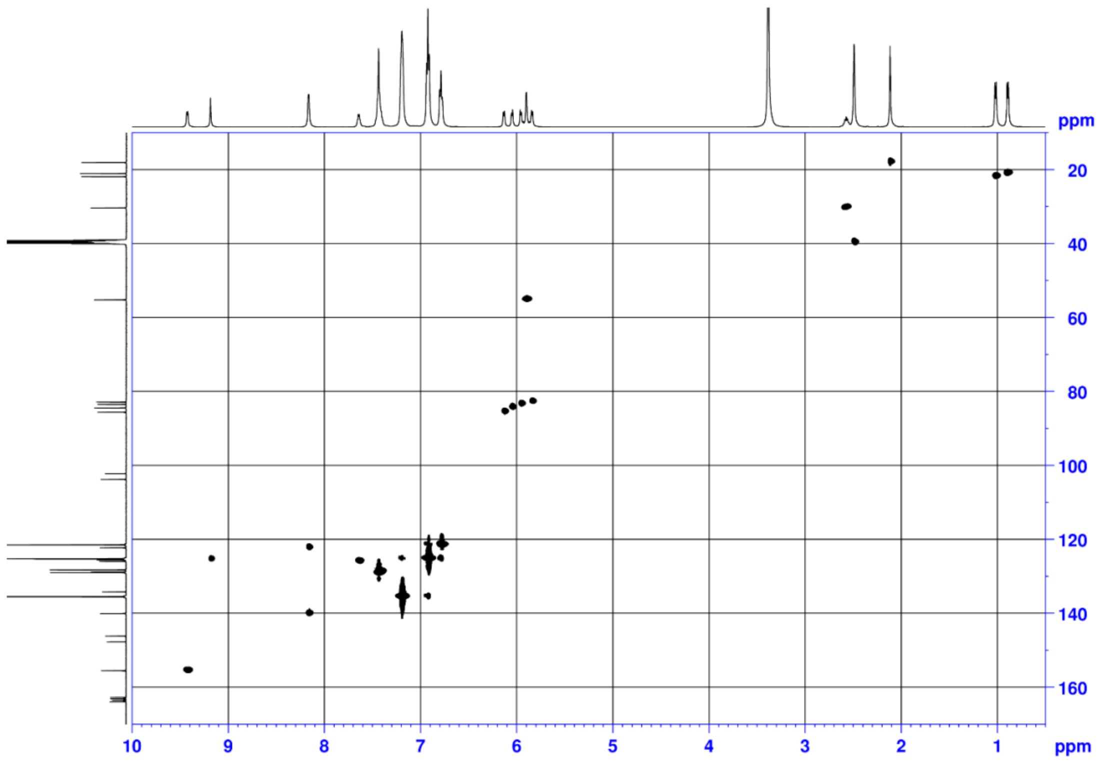

Figure S19: ^{1}H–^{13}C HSQC NMR spectrum of complex **C2** in DMSO-d_6.

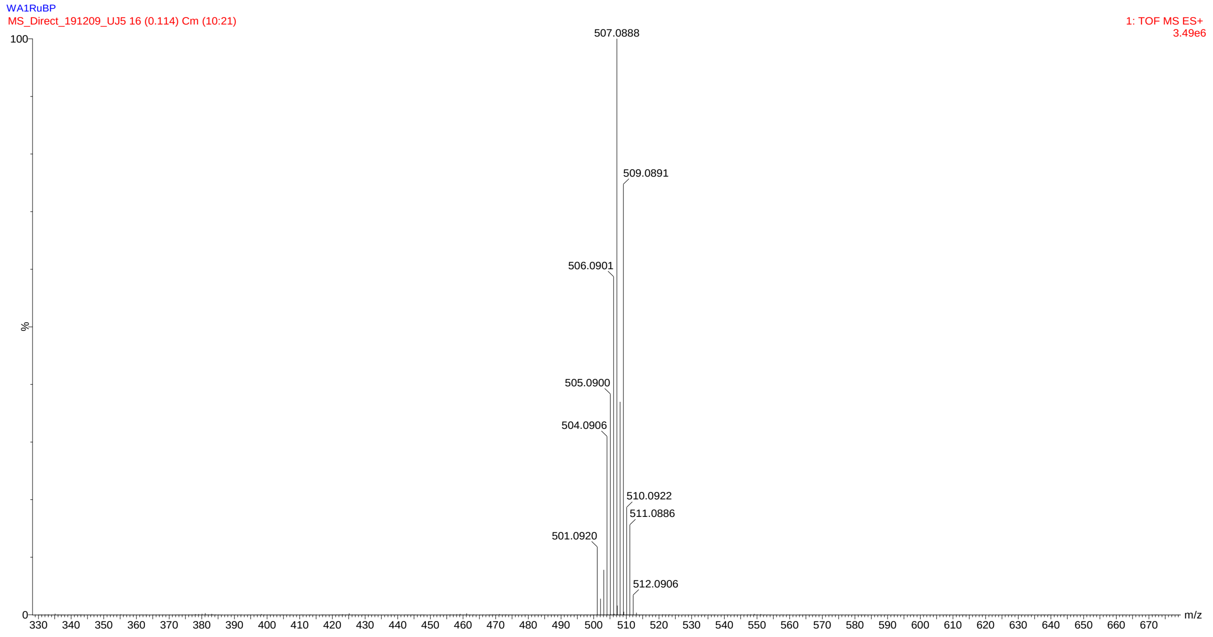

Figure S20: HRMS (ESI^{+}) of complex **C2.**

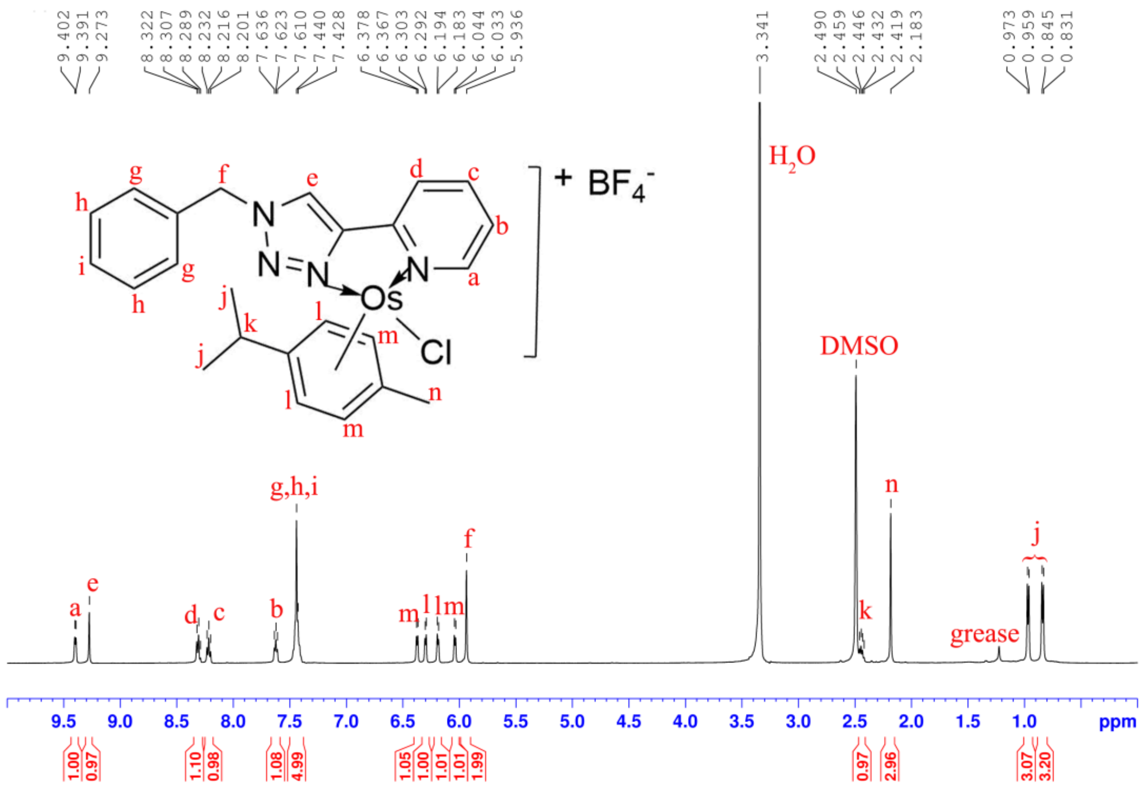

Figure S21: ^{1}H NMR spectrum of complex **C3** in DMSO-d_6.

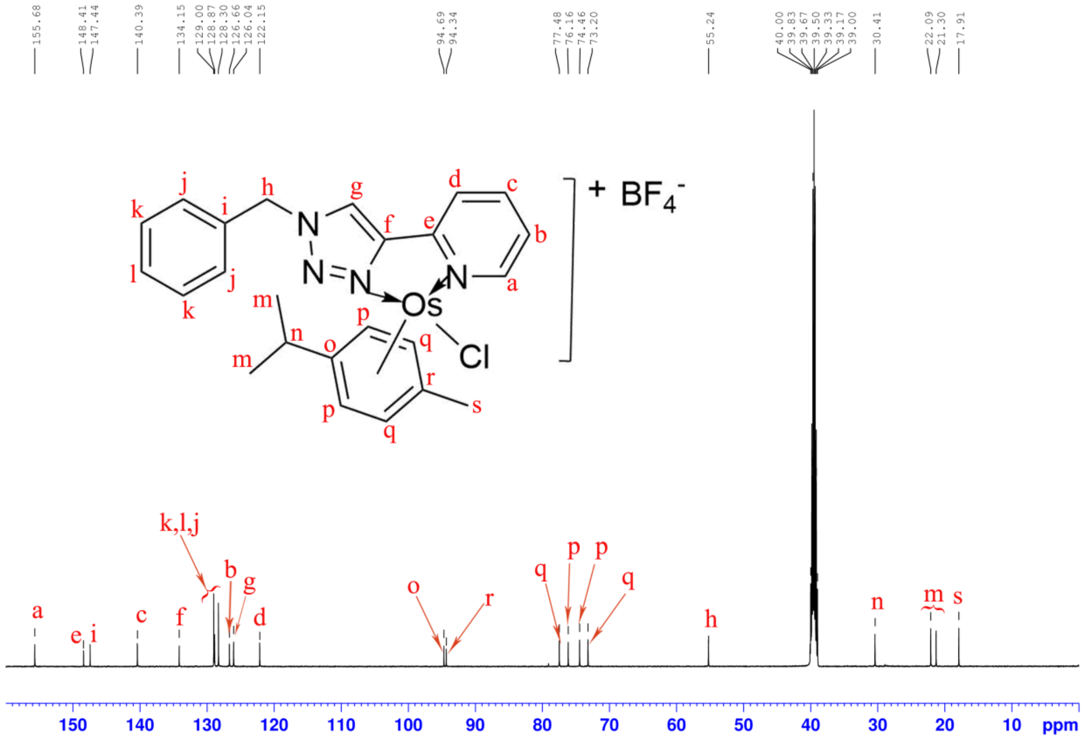

Figure S22: ^{13}C{^{1}H} NMR spectrum of complex **C3** in DMSO-d_6.

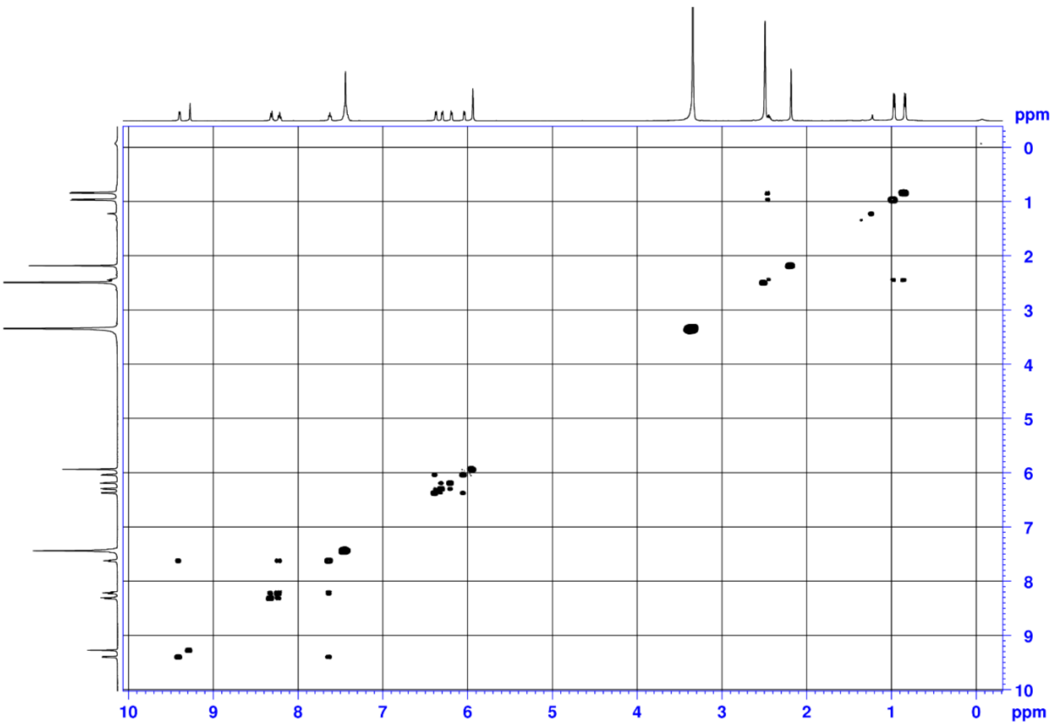

Figure S23: ^{1}H–^{1}H COSY NMR spectrum of complex **C3** in DMSO-d_6.

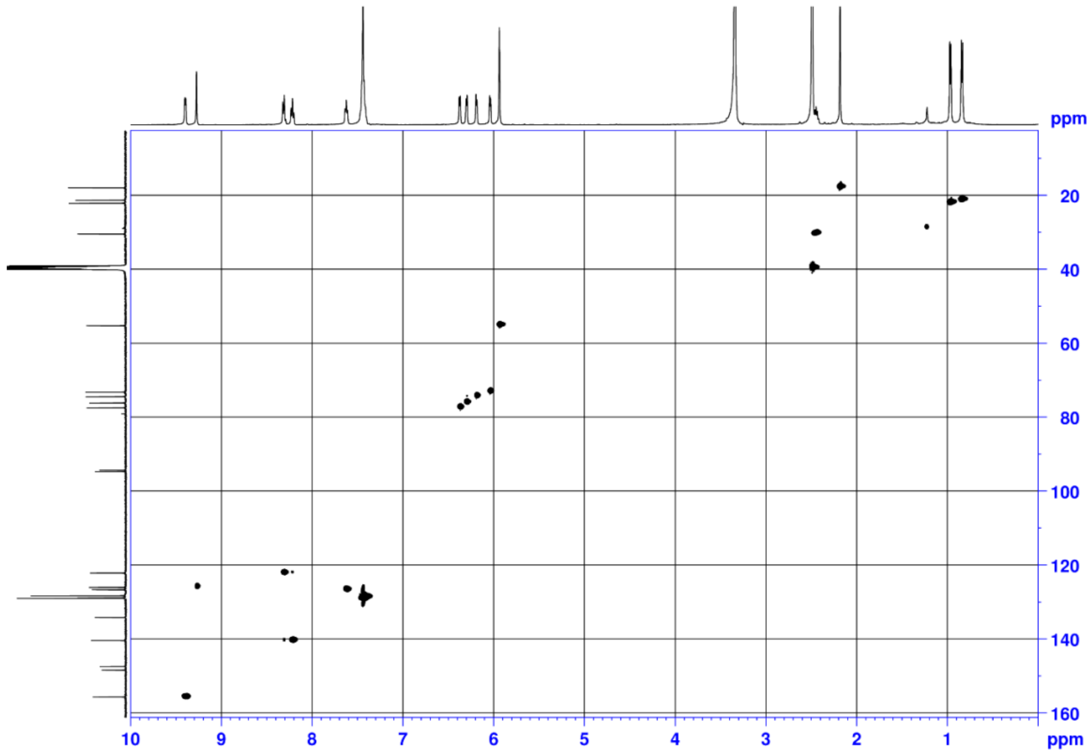

Figure S24: ^{1}H–^{13}C HSQC NMR spectrum of complex **C3** in DMSO-d_6.

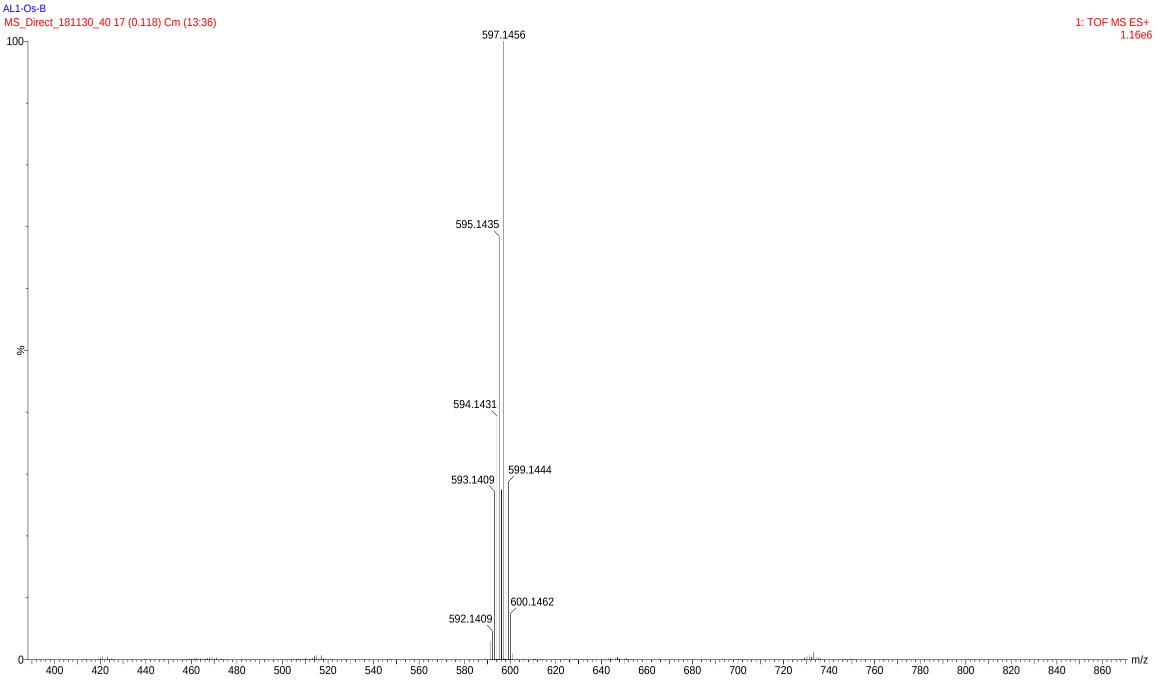

Figure S25: HRMS (ESI^+) of complex **C3.**

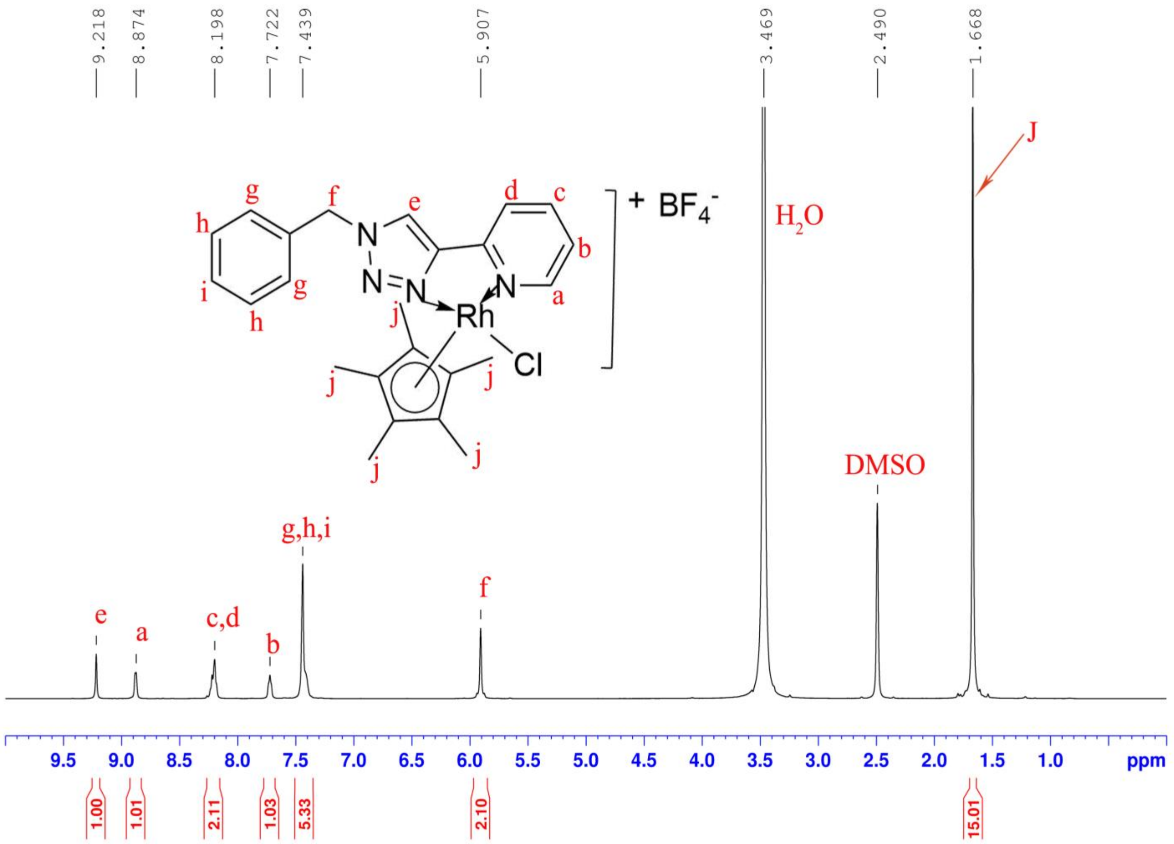

Figure S26: ^1H NMR spectrum of complex **C4** in DMSO-d_6.

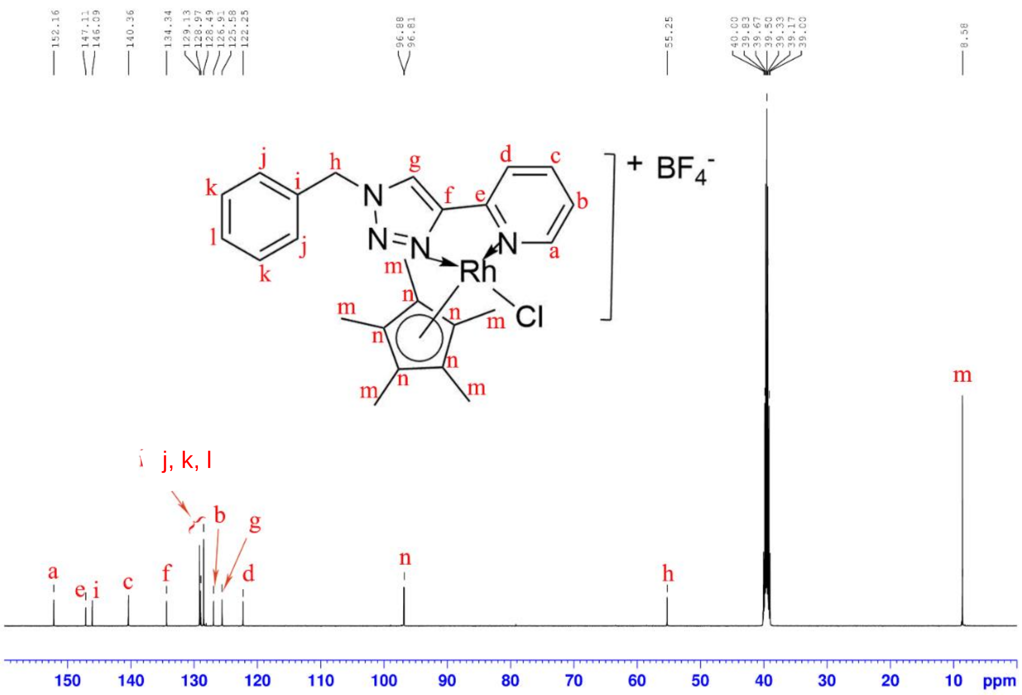

Figure S27: $^{13}C\{^{1}H\}$ NMR spectrum of complex **C4** in DMSO-d_6.

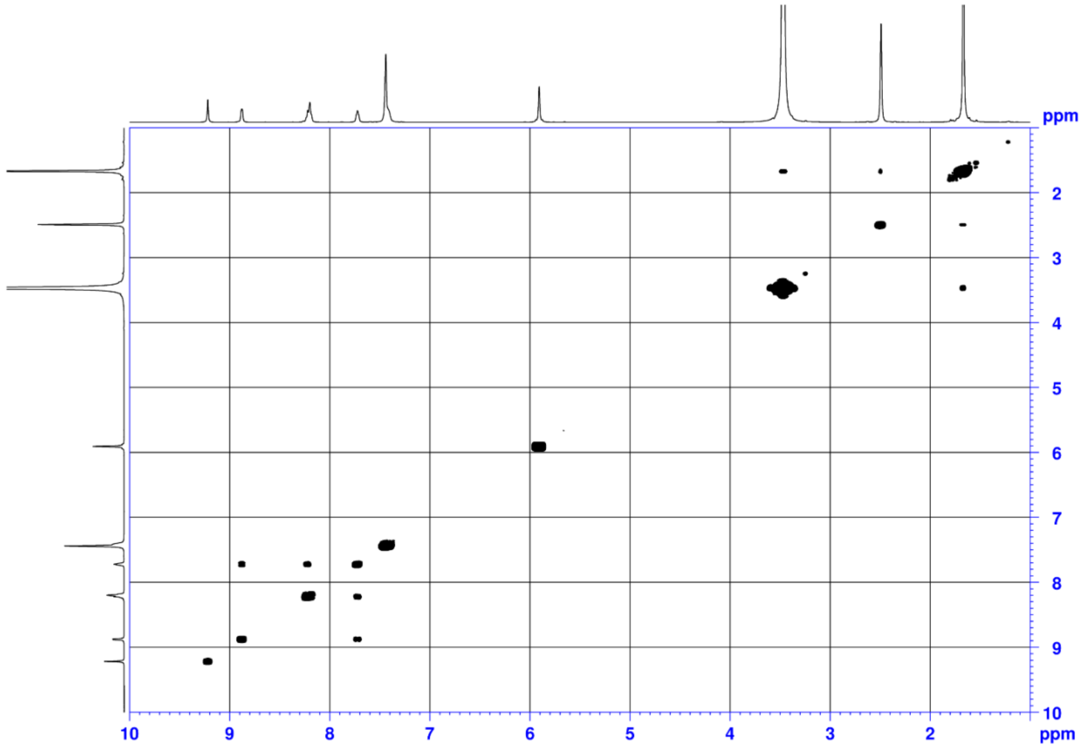

Figure S28: ^{1}H–^{1}H COSY NMR spectrum of complex **C4** in DMSO-d_6.

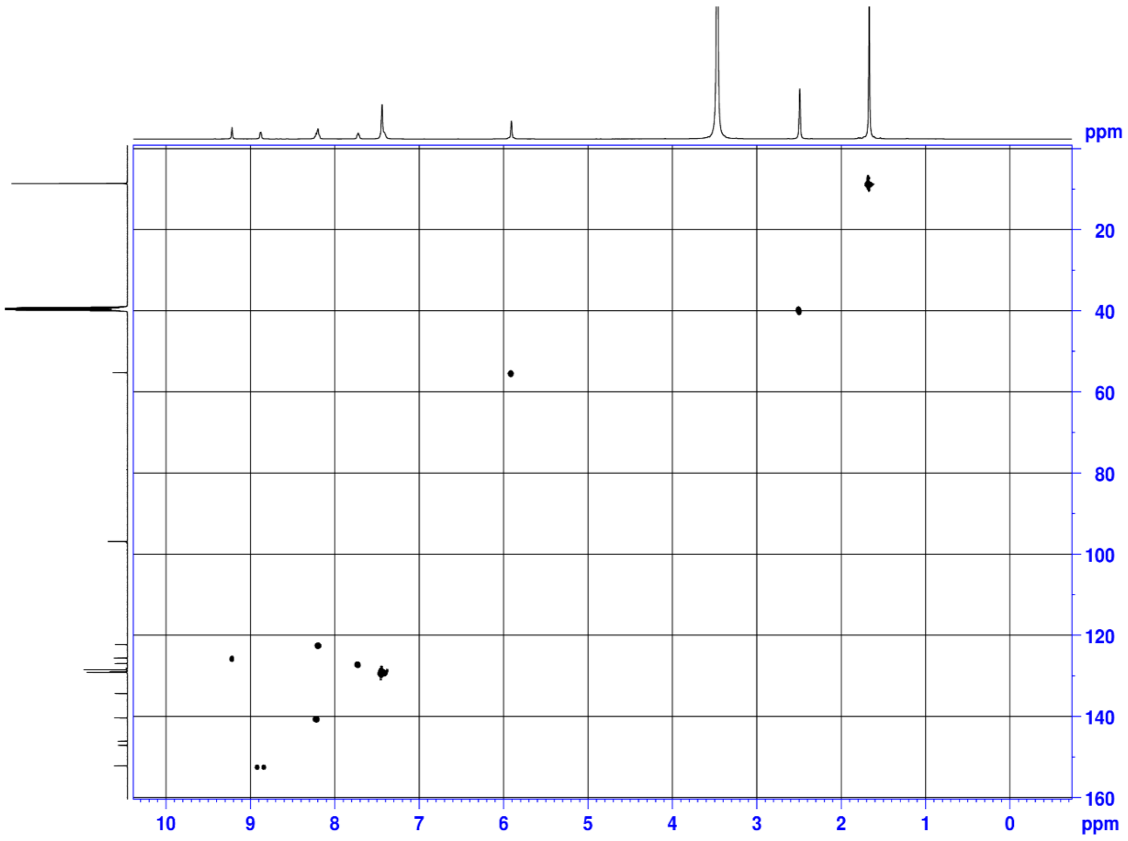

Figure S29: ^{1}H–^{13}C HSQC NMR spectrum of complex **C4** in DMSO-d_6.

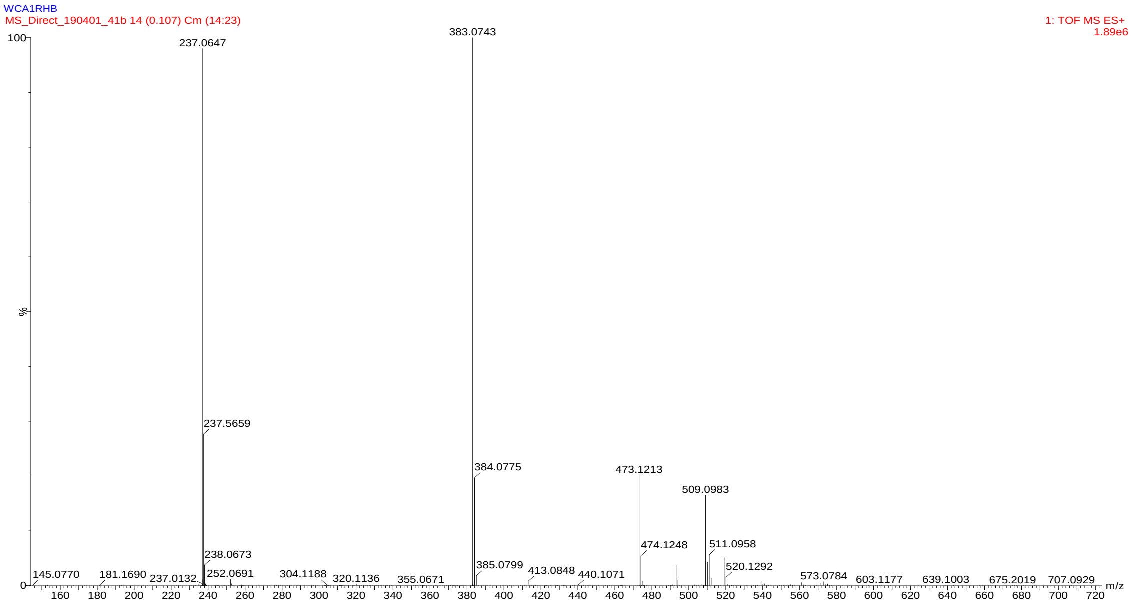

Figure S30: HRMS (ESI^{+}) of complex **C4.**

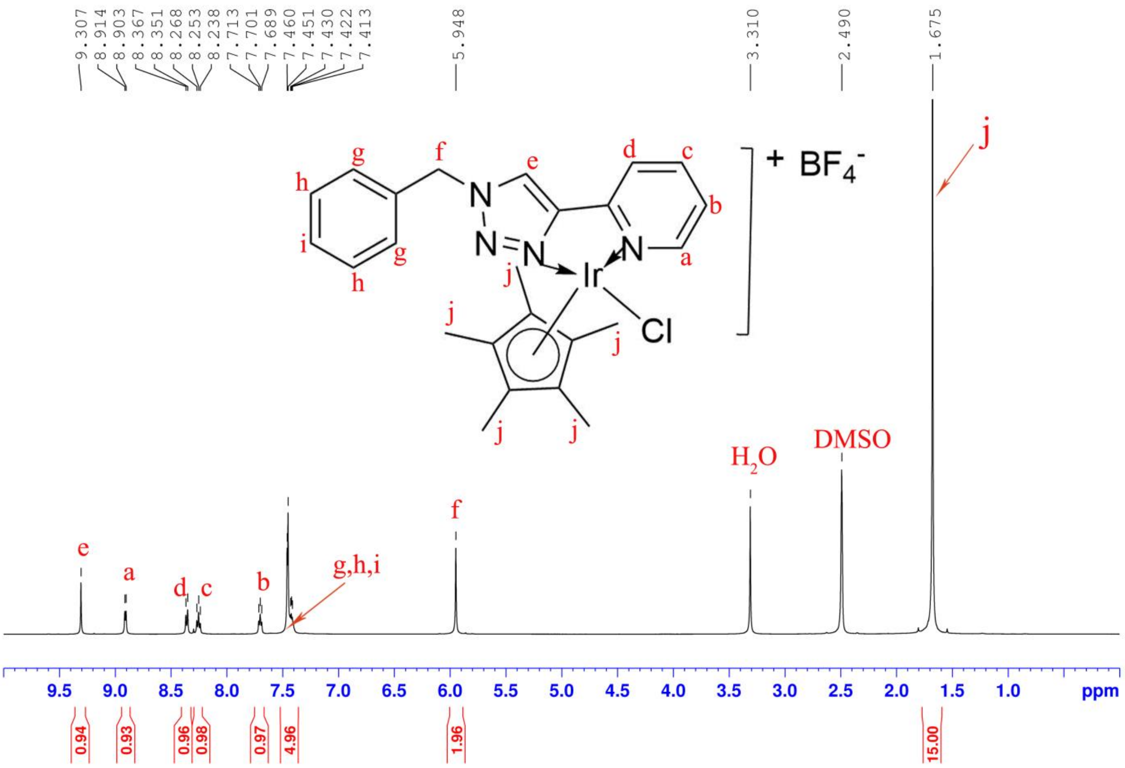

Figure S31: ^{1}H NMR spectrum of complex **C5** in DMSO-d_6.

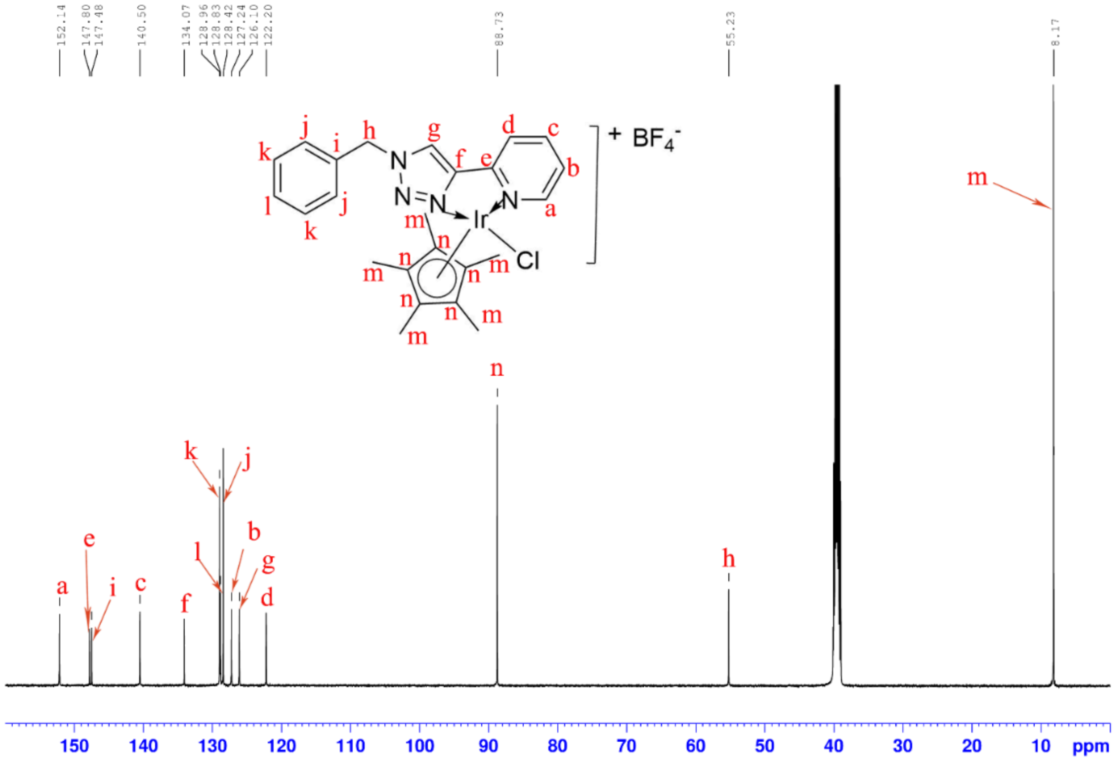

Figure S32: $^{13}C\{^{1}H\}$ NMR spectrum of complex **C5** in DMSO-d_6.

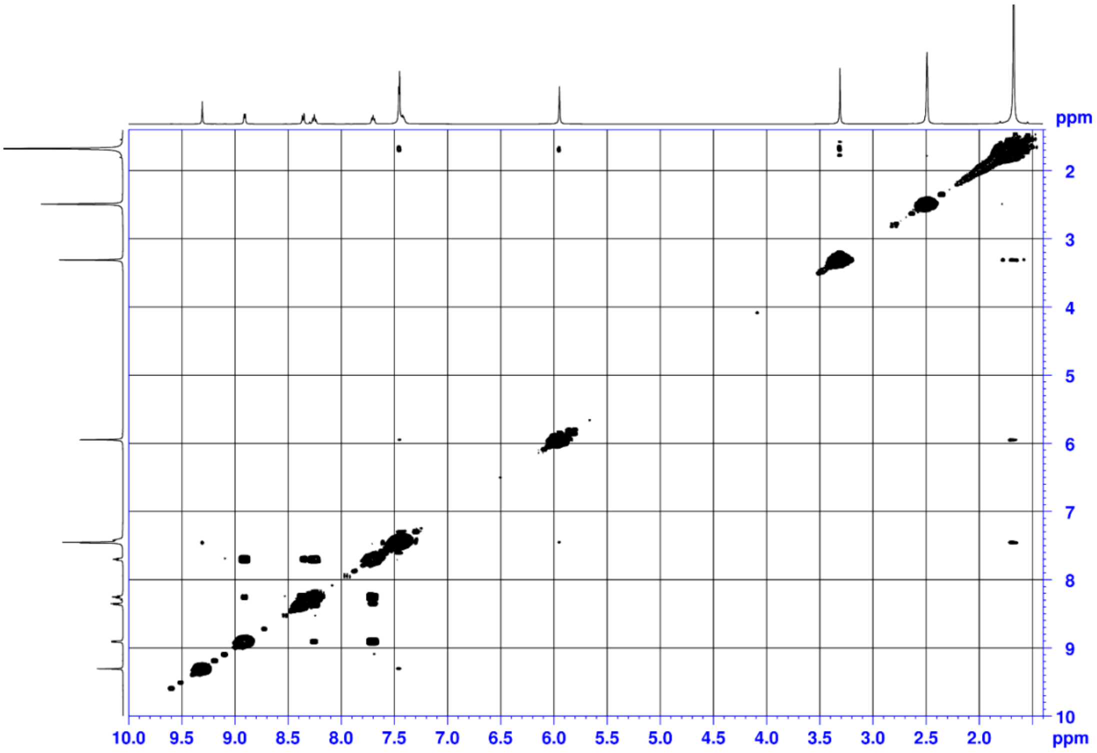

Figure S33: ^{1}H–^{1}H COSY NMR spectrum of complex **C5** in DMSO-d_6.

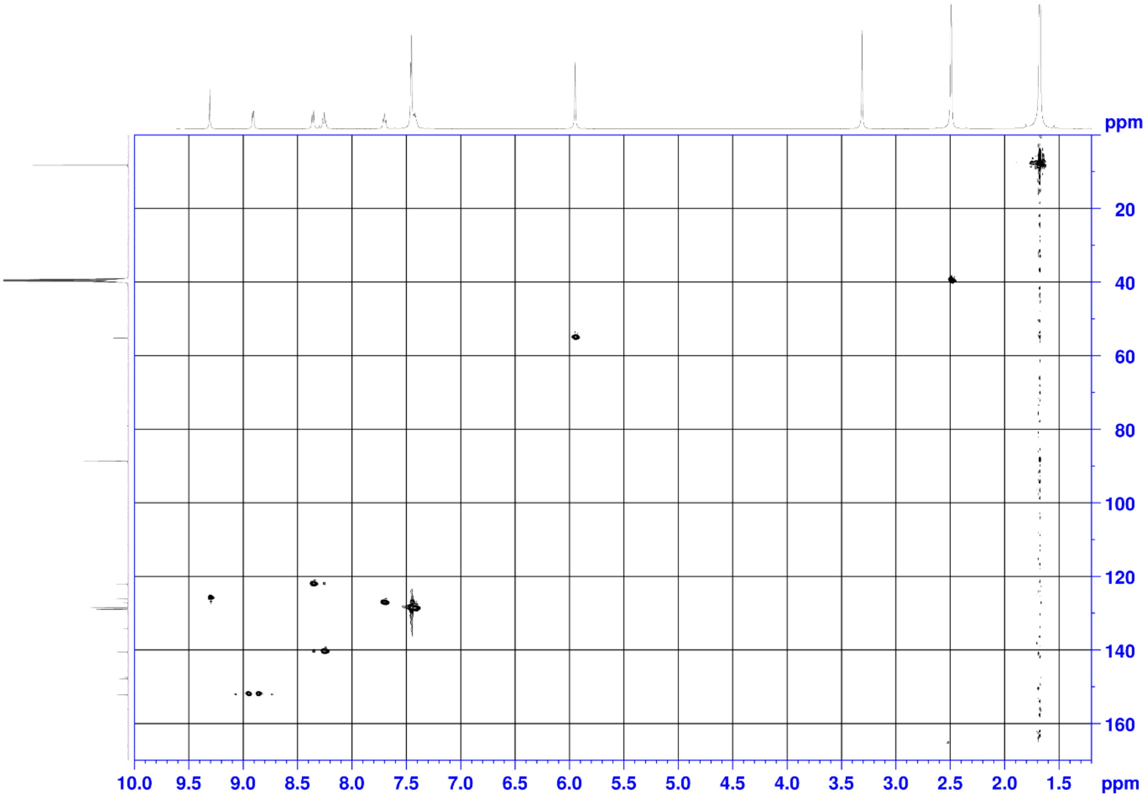

Figure S34: ^{1}H–^{13}C HSQC NMR spectrum of complex **C5** in DMSO-d_6.

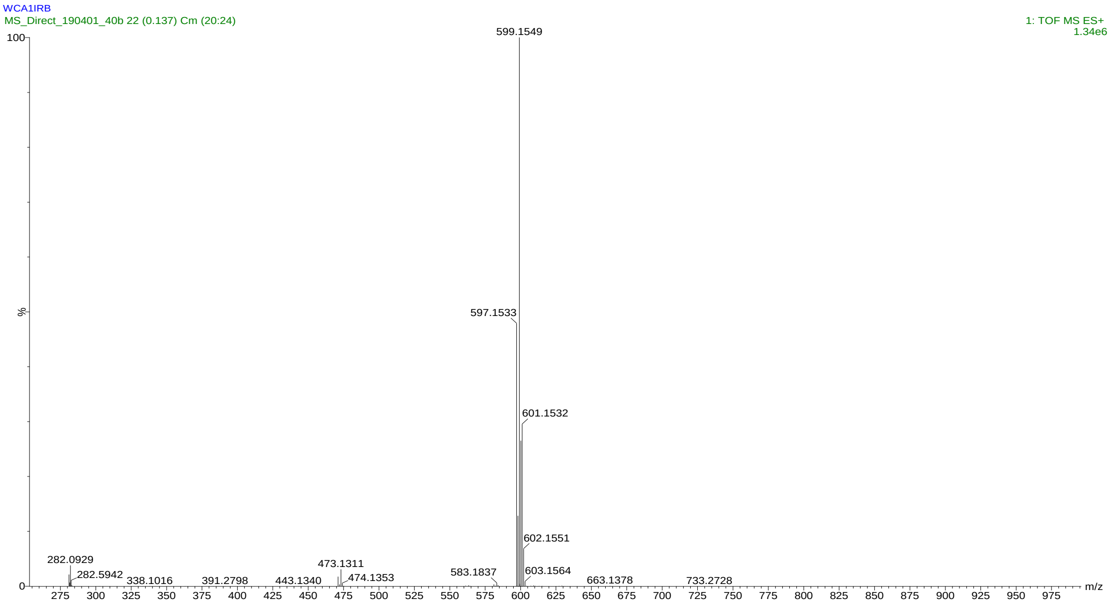

Figure S35: HRMS (ESI+) of complex **C5.**

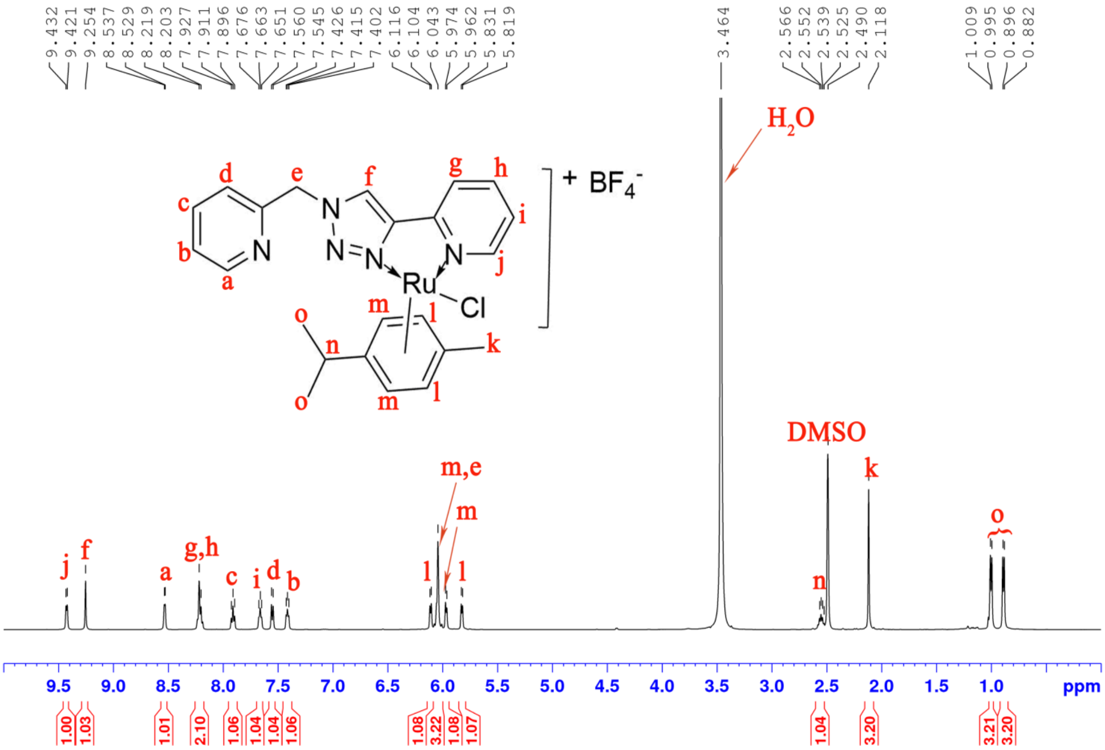

Figure S36: ^{1}H NMR spectrum of complex **C6** in DMSO-d_6.

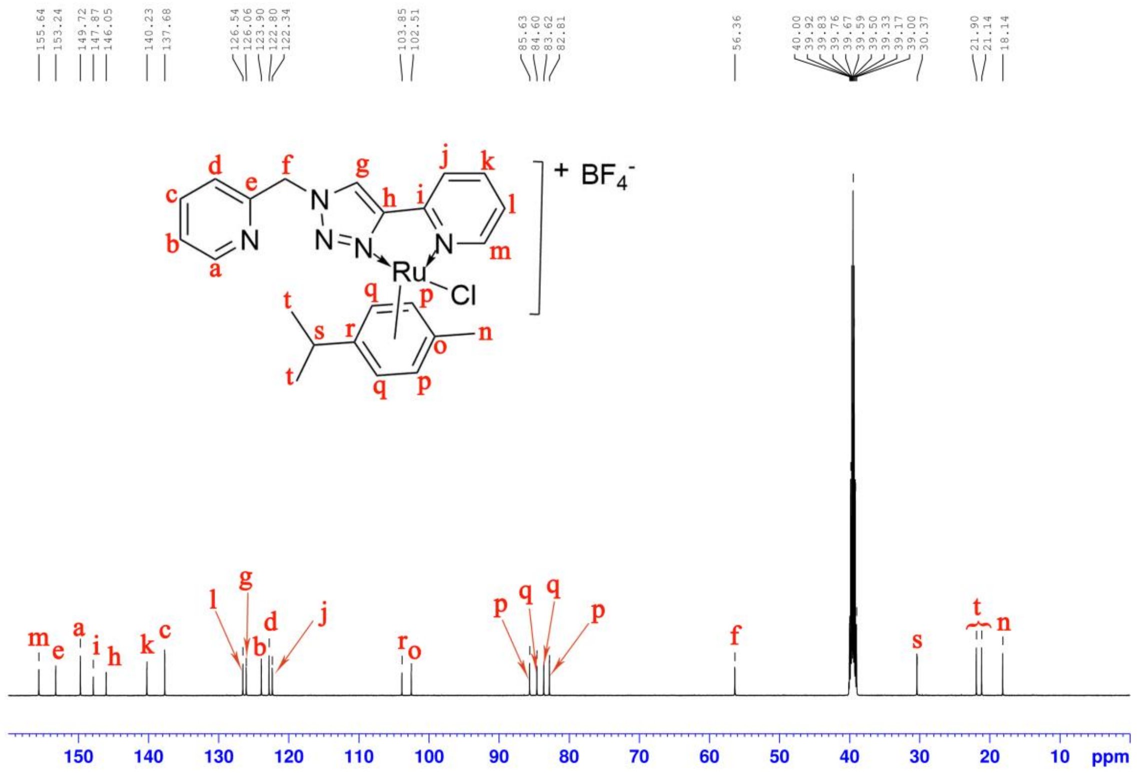

Figure S37: $^{13}C\{^{1}H\}$ NMR spectrum of **C6** in DMSO-d_6.

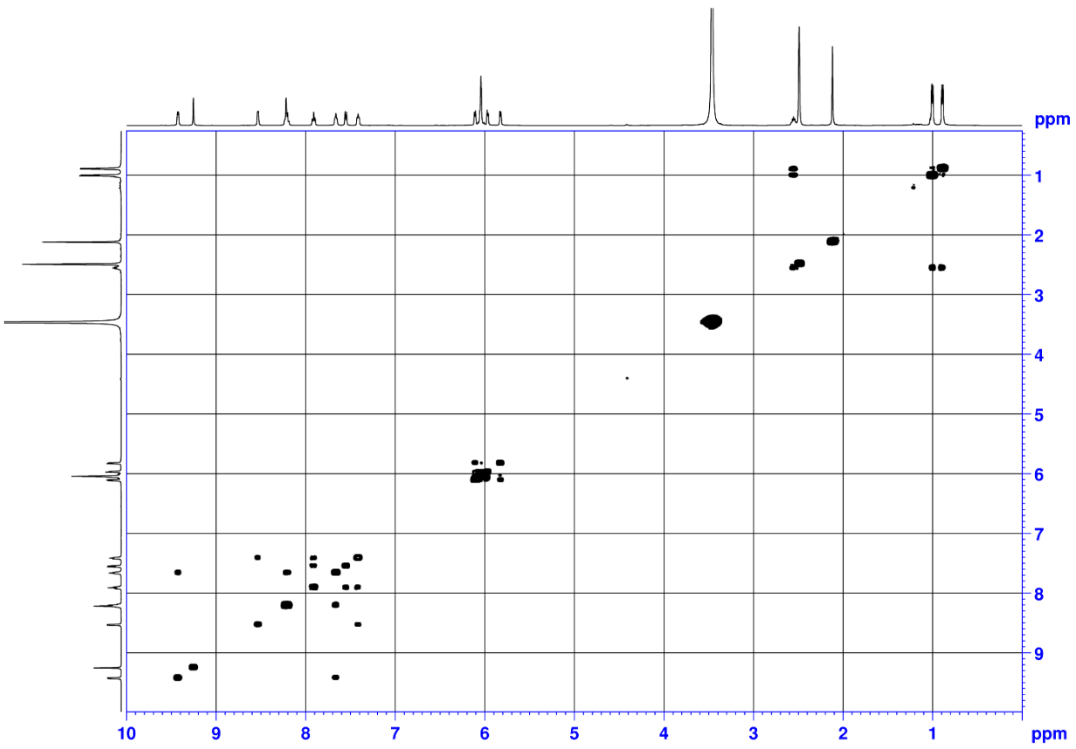

Figure S38: ^{1}H–^{1}H COSY NMR spectrum of complex **C6** in DMSO-d_6.

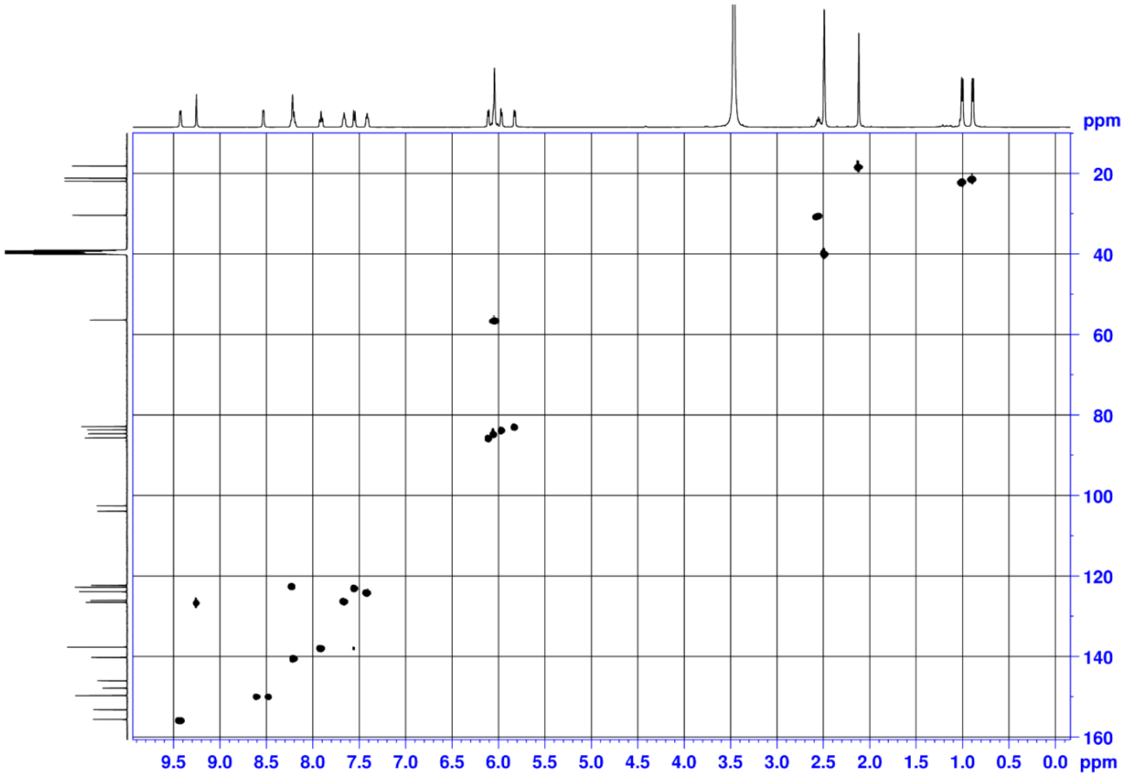

Figure S39: ^{1}H–^{13}C HSQC NMR spectrum of complex **C6** in DMSO-d_6.

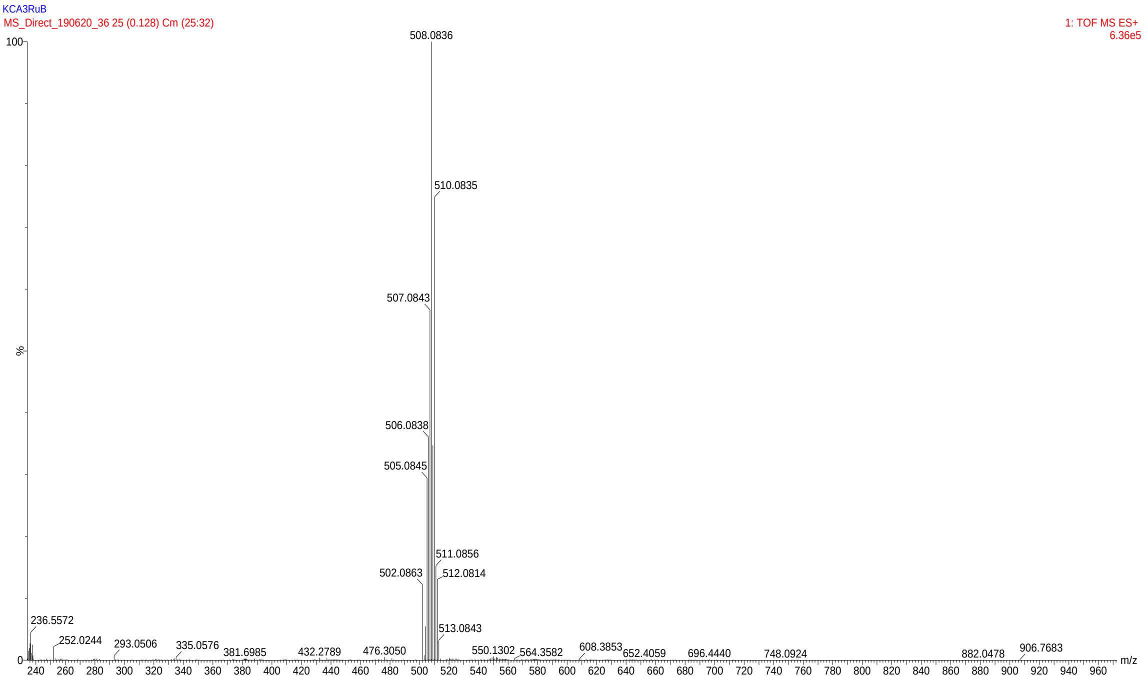

Figure S40: HRMS (ESI^{+}) of complex **C6.**

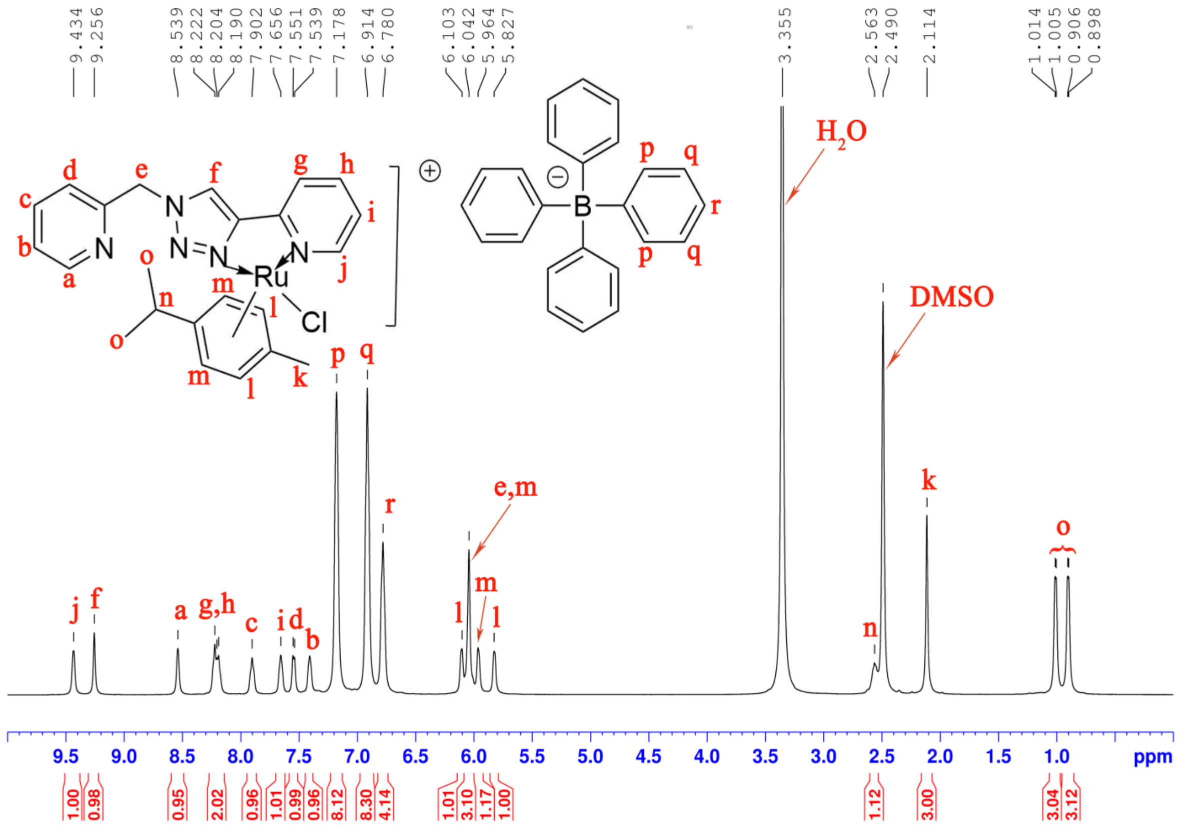

Figure S41: ^{1}H NMR spectrum of complex **C7** in DMSO-*d*6.

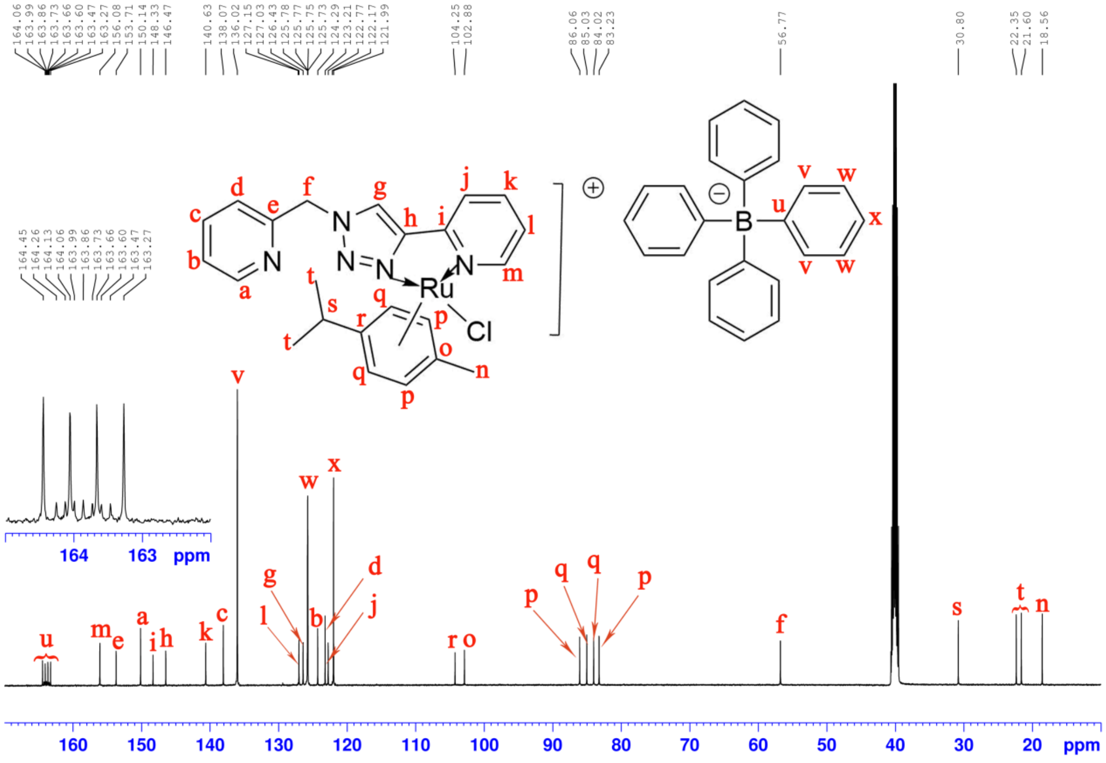

Figure S42: ^{13}C{^{1}H} NMR spectrum of complex **C7** in DMSO-*d*6.

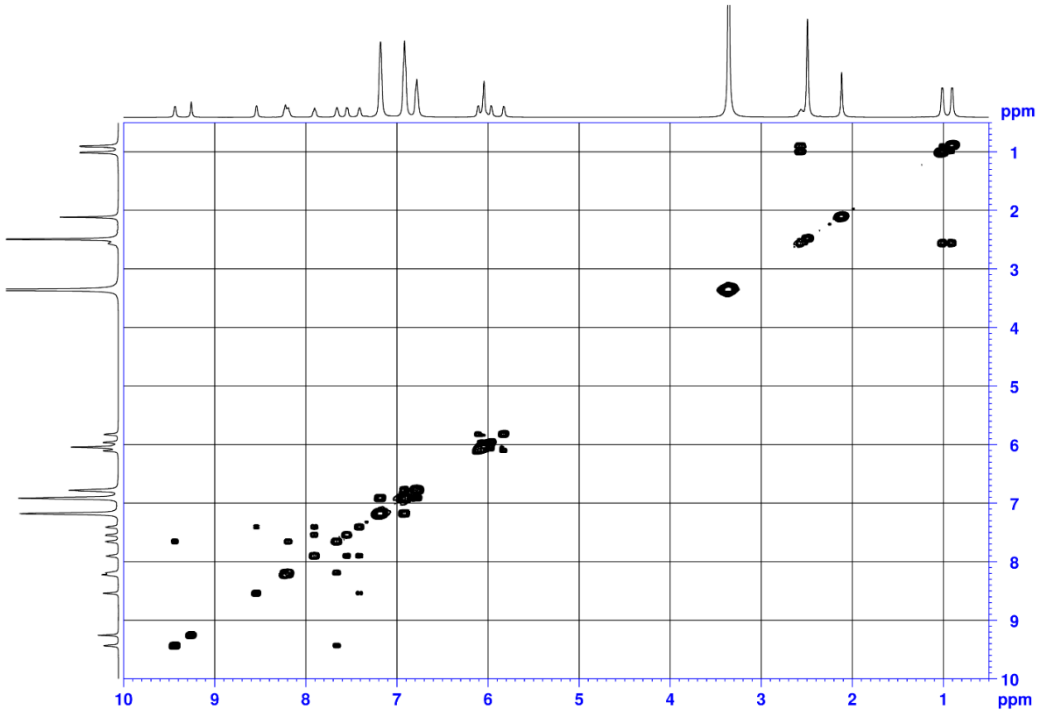

Figure S43: ^{1}H–^{1}H COSY NMR spectrum of complex **C7** in DMSO-d_6.

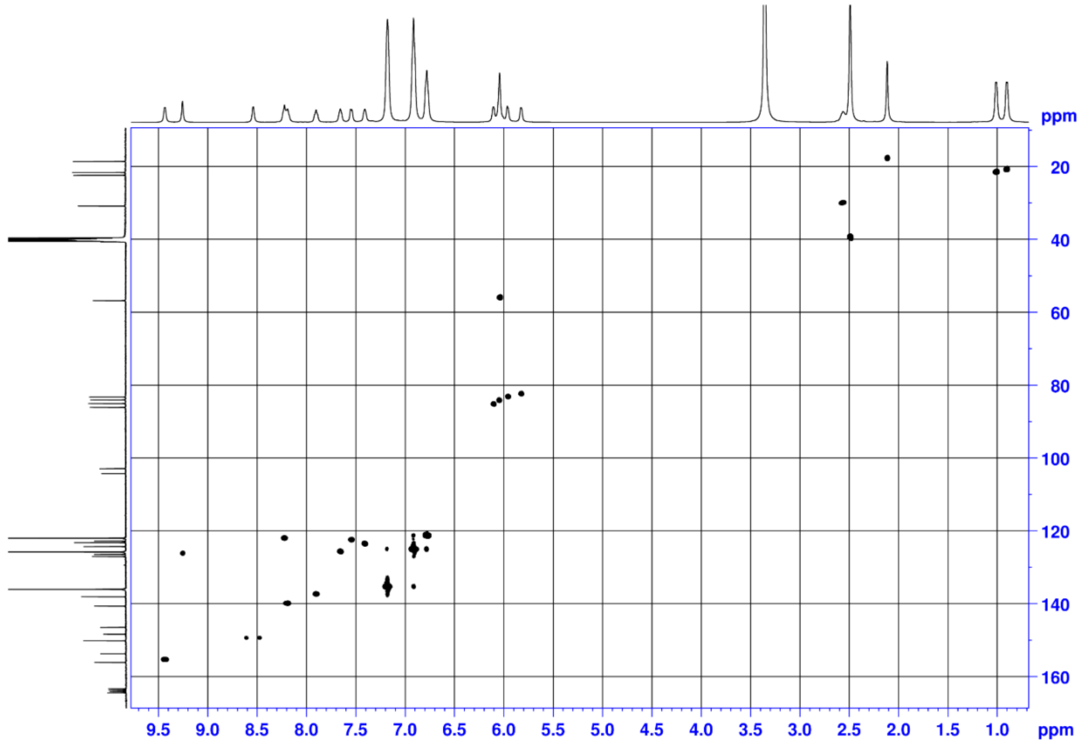

Figure S44: ^{1}H–^{13}C HSQC NMR spectrum of complex **C7** in DMSO-d_6.

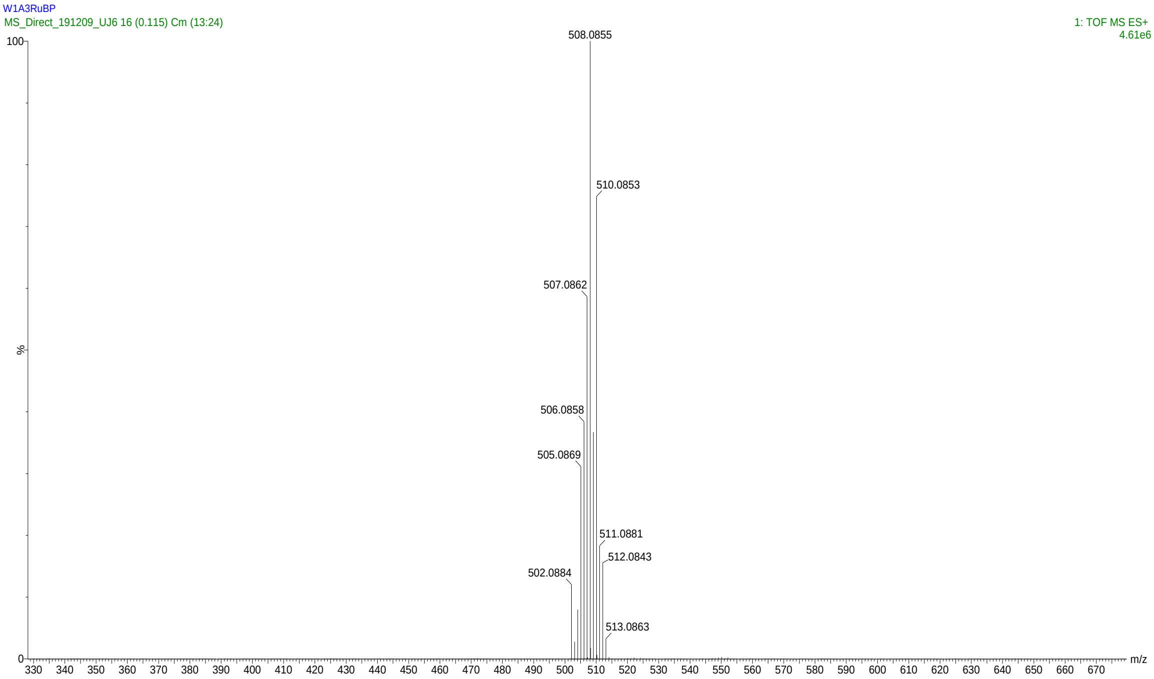

Figure S45: HRMS (ESI^+) of complex **C7.**

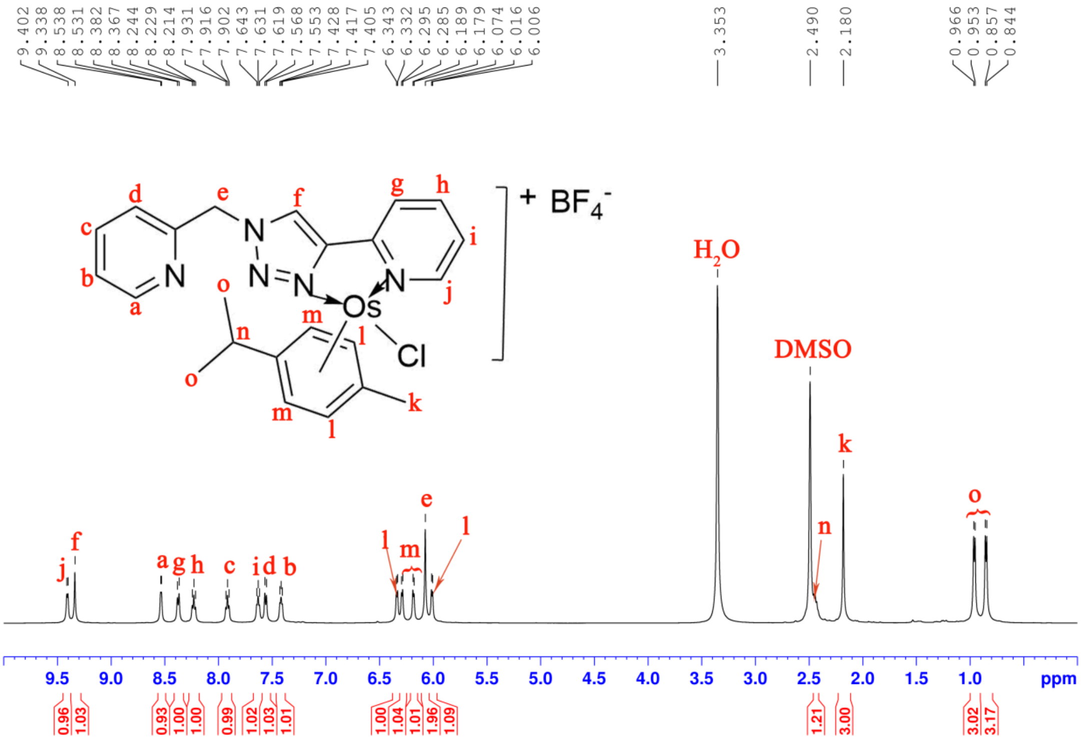

Figure S46: ^1H NMR spectrum of complex **C8** in DMSO-d_6.

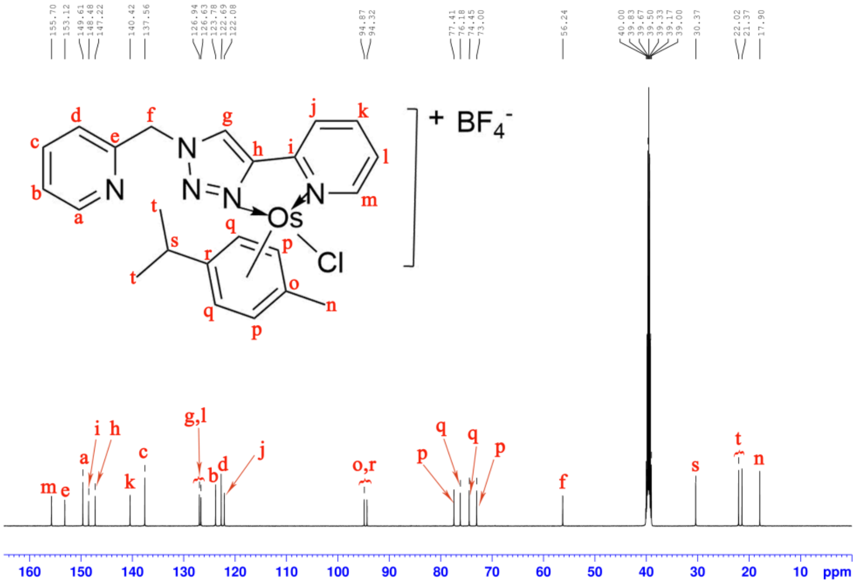

Figure S47: $^{13}C\{^{1}H\}$ NMR spectrum of complex **C8** in DMSO-d_6.

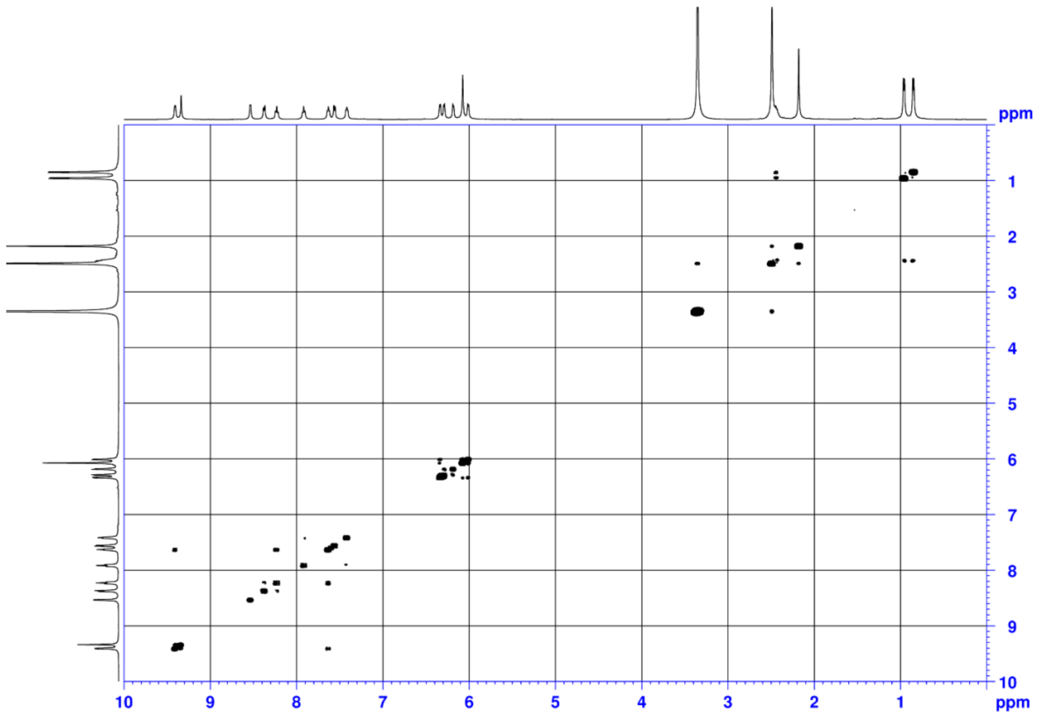

Figure S48: ^{1}H–^{1}H COSY NMR spectrum of complex **C8** in DMSO-d_6.

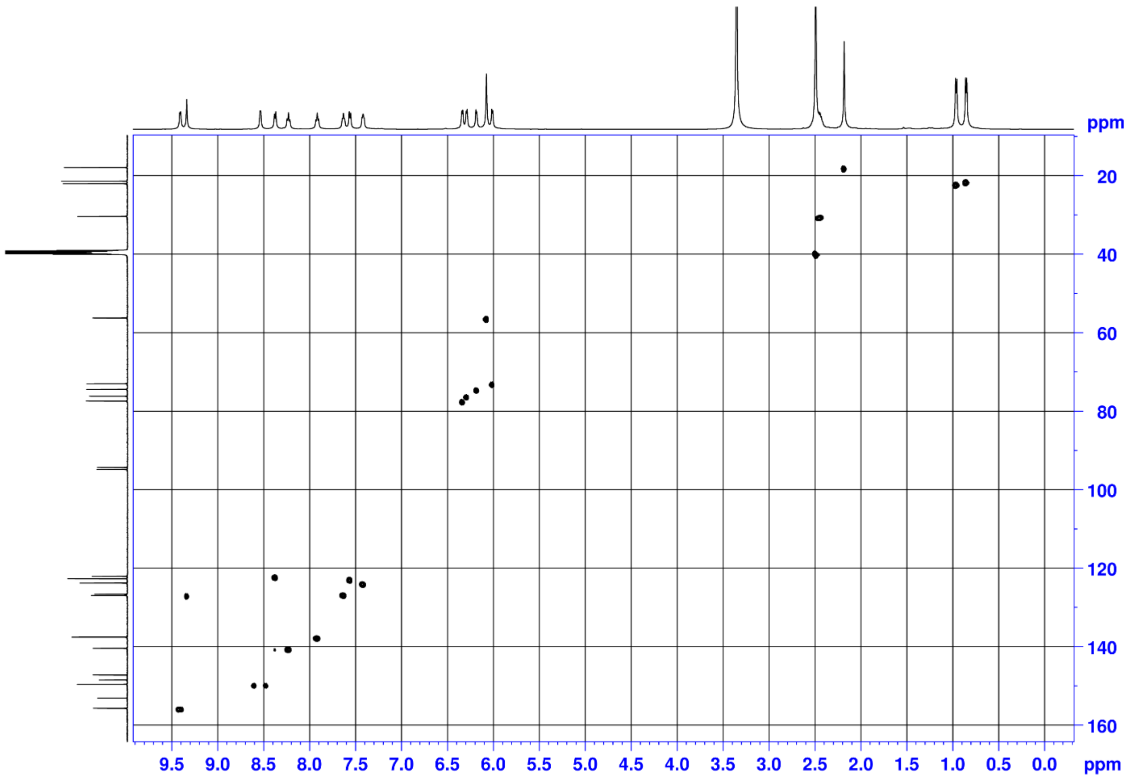

Figure S49: ^{1}H–^{13}C HSQC NMR spectrum of complex **C8** in DMSO-d_6.

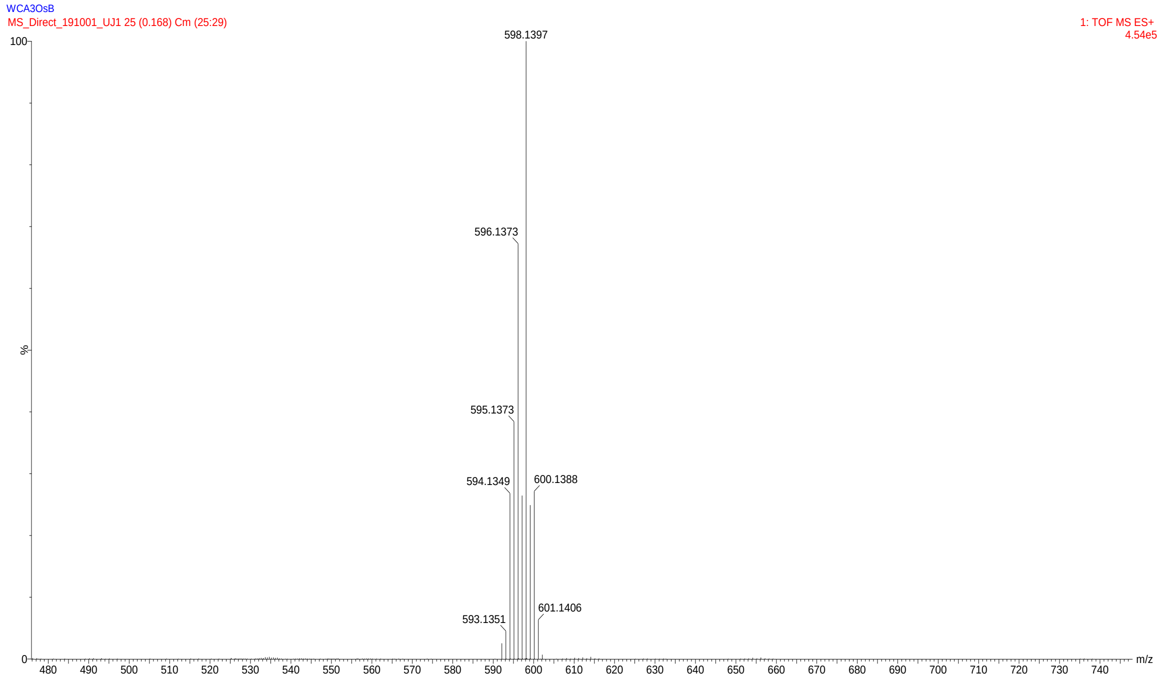

Figure S50: HRMS (ESI^{+}) of complex **C8.**

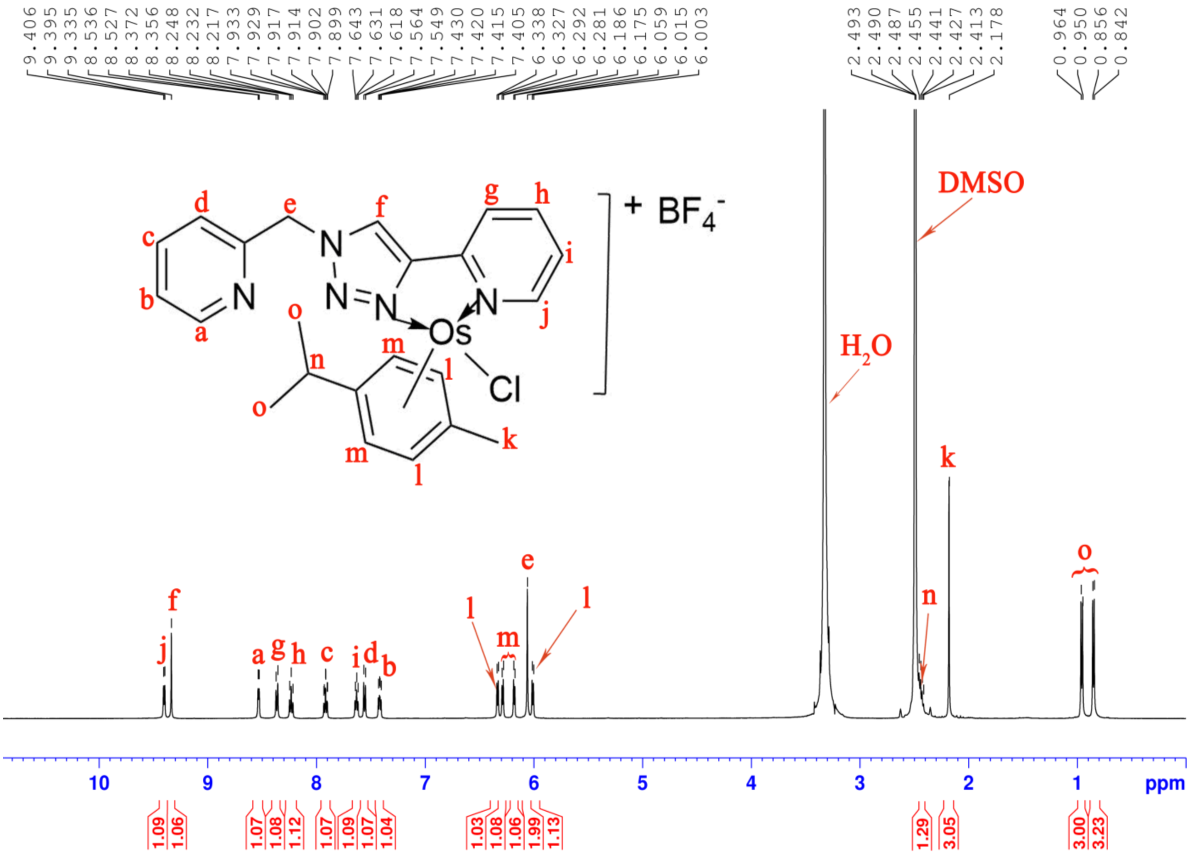

Figure S51: ^{1}H NMR spectrum of complex **C9** in DMSO-*d*6.

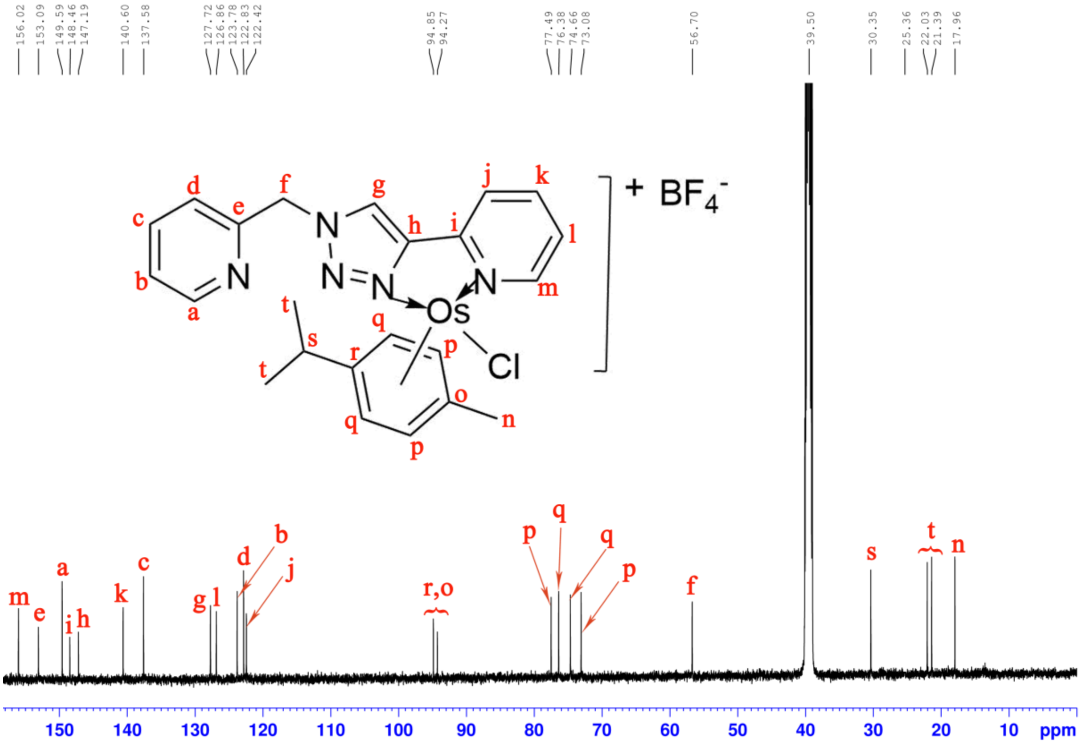

Figure S52: ^{13}C{^{1}H} NMR spectrum of complex **C9** in DMSO-*d*6

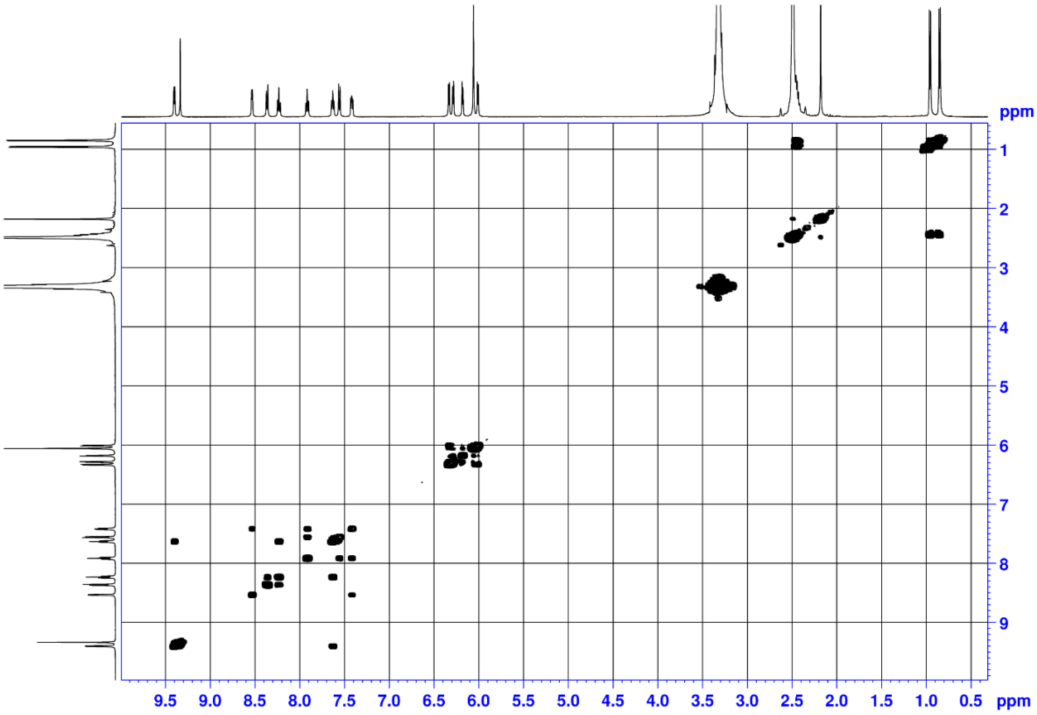

Figure S53: ^{1}H–^{1}H COSY NMR spectrum of complex **C9** in DMSO-d_6.

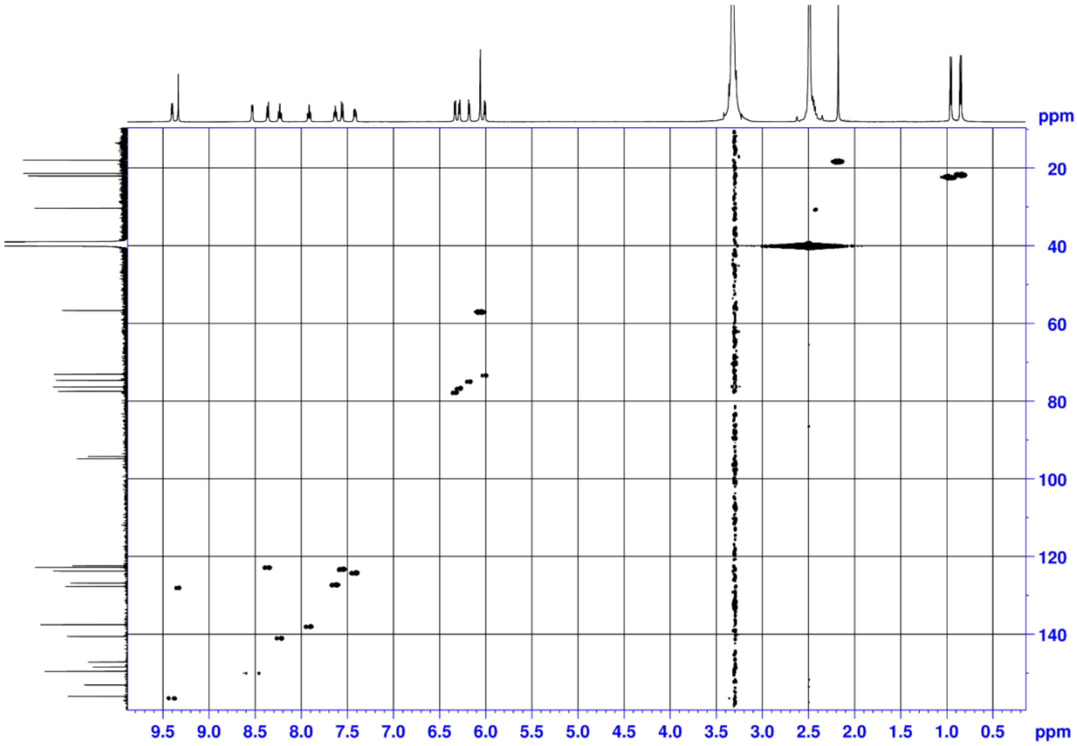

Figure S54: ^{1}H–^{13}C HSQC NMR spectrum of complex **C9** in DMSO-d_6.

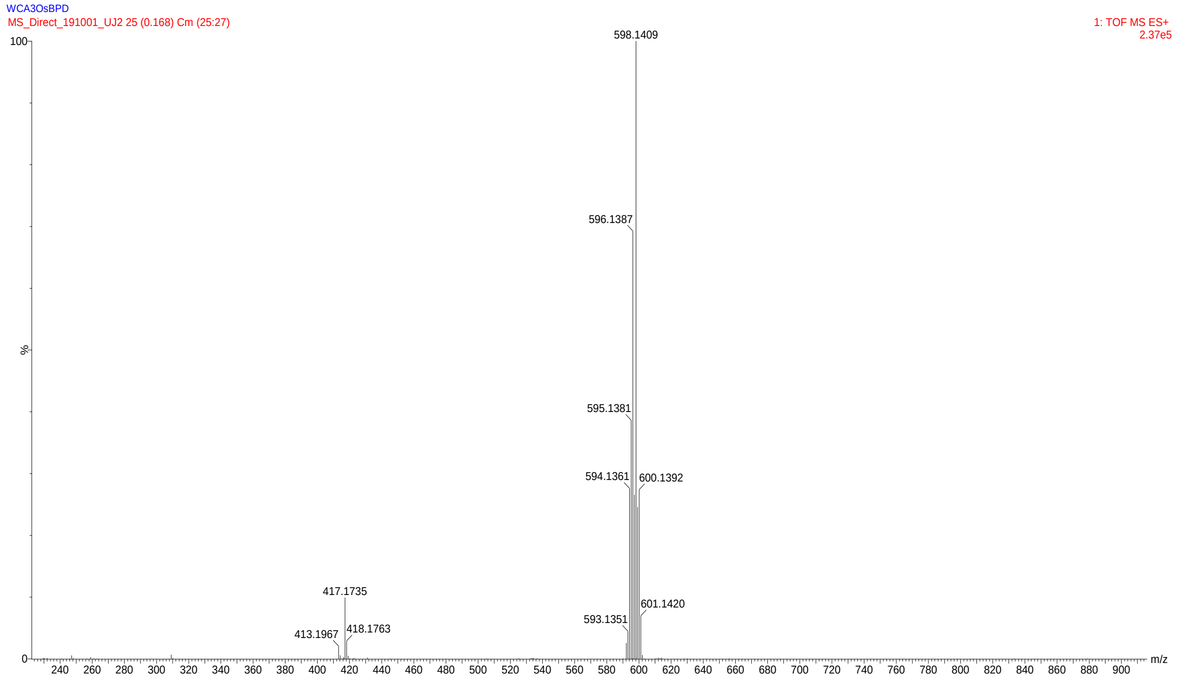

Figure S55: HRMS(ESI^+) of complex **C9.**

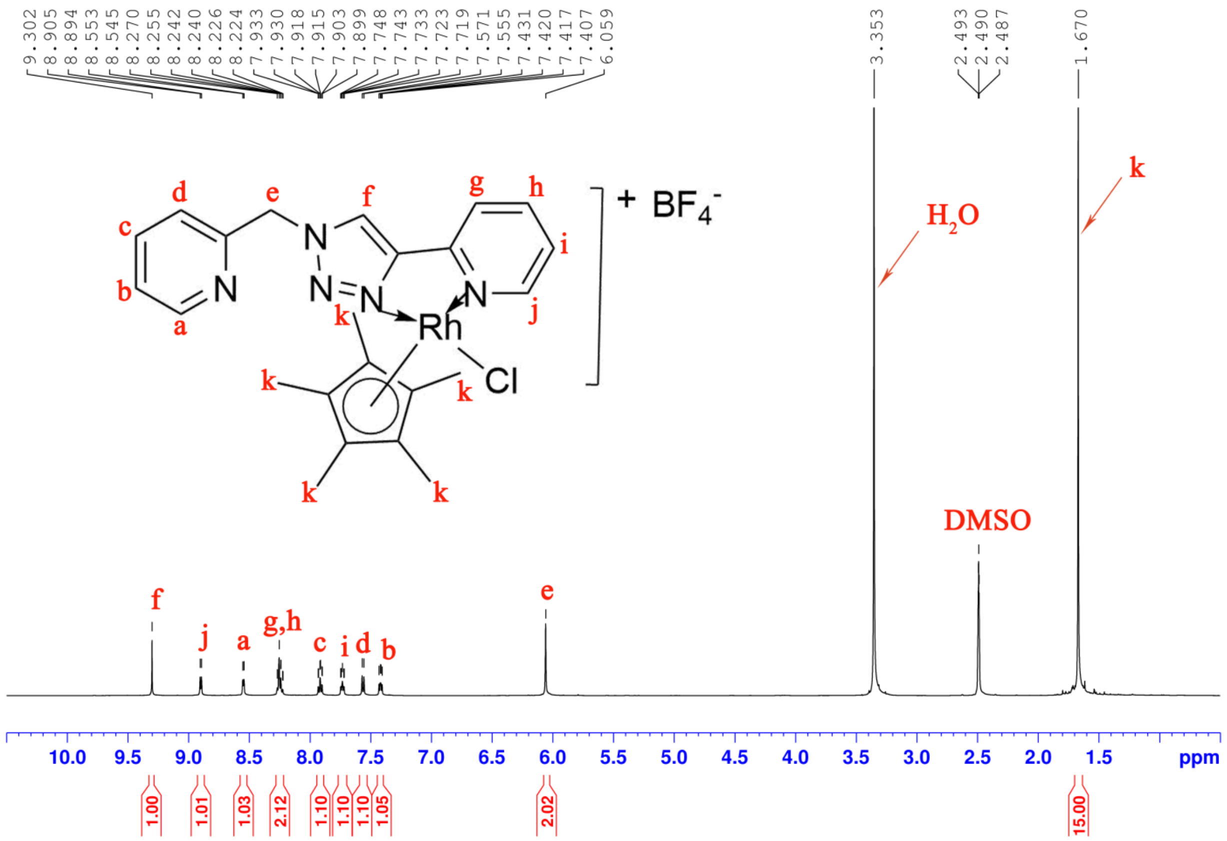

Figure S56: ^{1}H NMR spectrum of complex **C10** in DMSO-d_6.

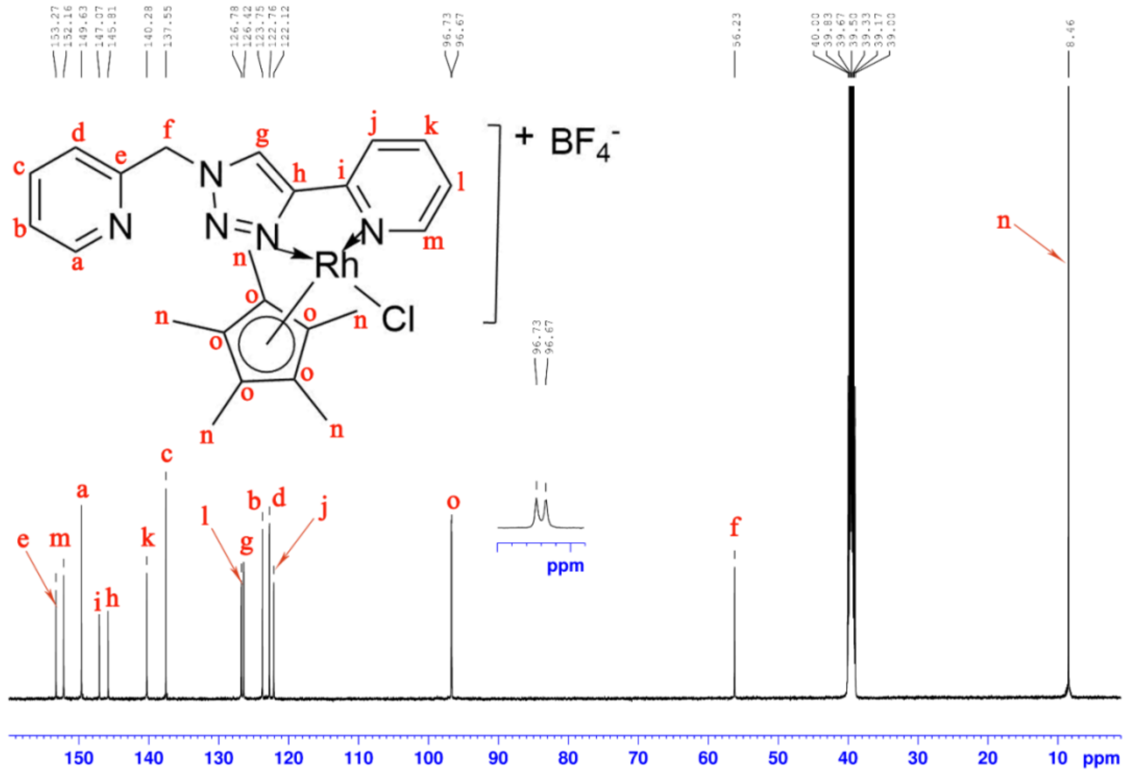

Figure S57: $^{13}C\{^{1}H\}$ NMR spectrum of complex **C10** in DMSO-d_6.

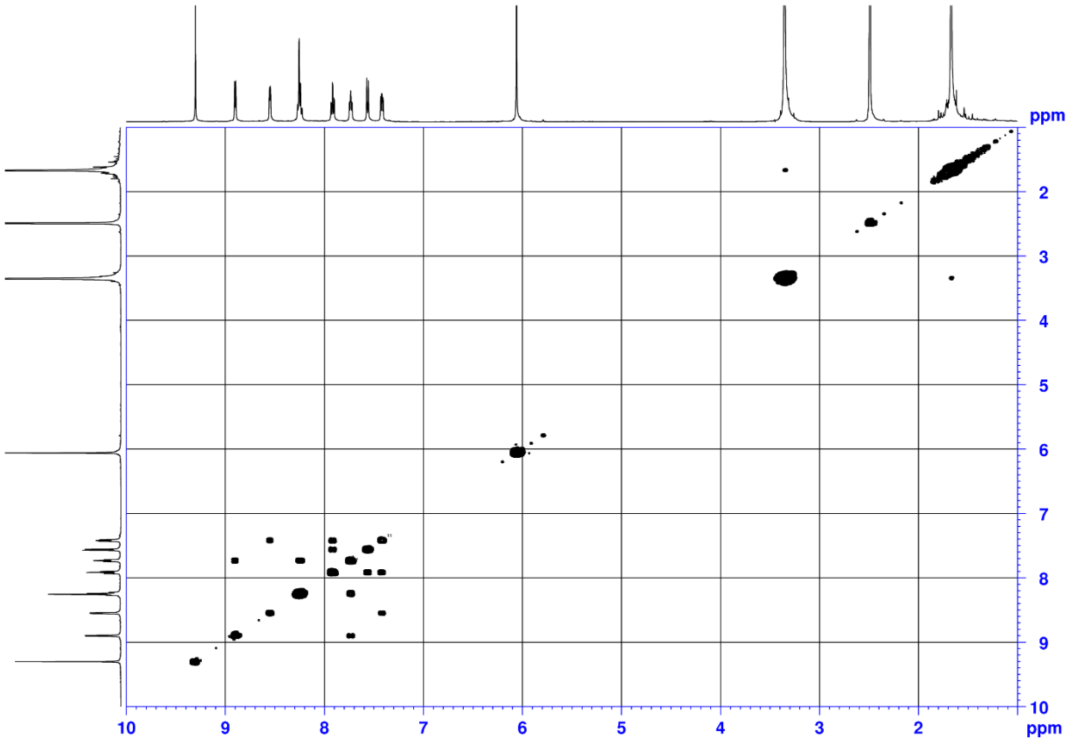

Figure S58: ^{1}H–^{1}H COSY NMR spectrum of complex **C10** in DMSO-d_6.

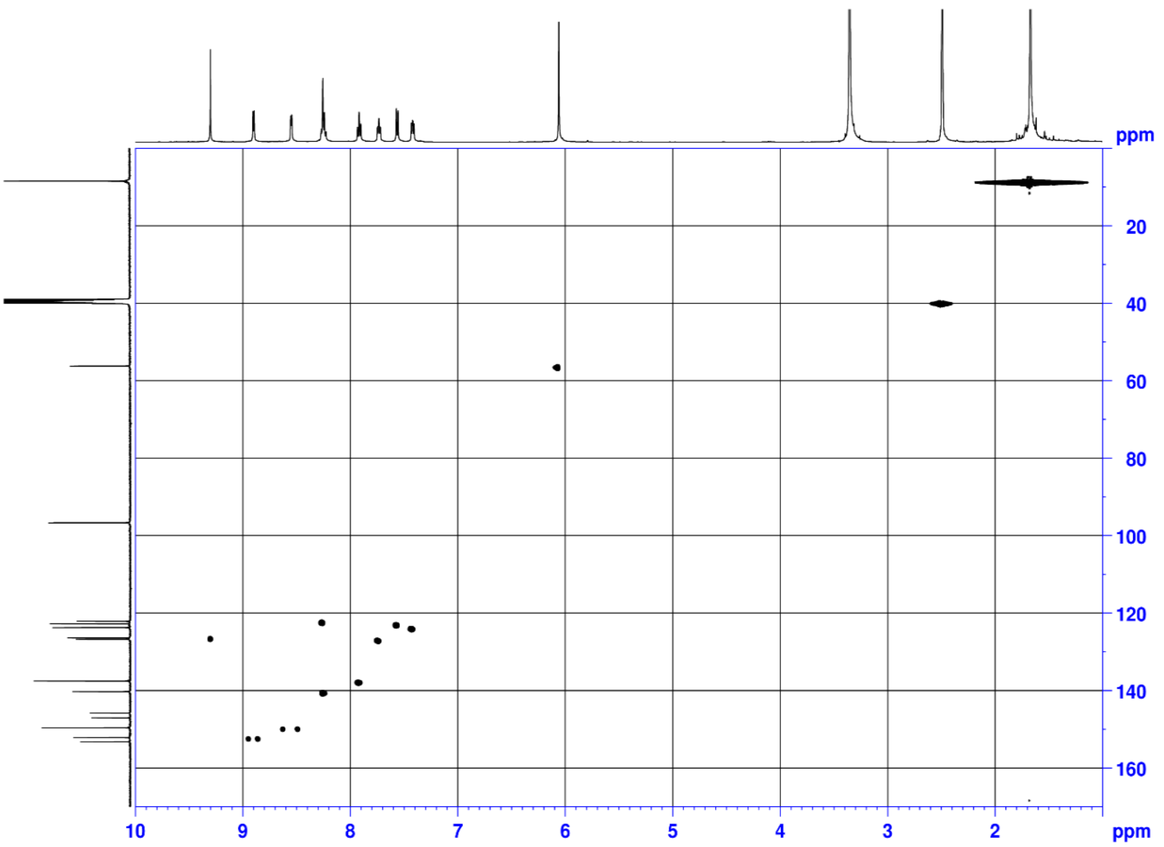

Figure S59: ^{1}H–^{13}C HSQC NMR spectrum of complex **C10** in DMSO-d_6.

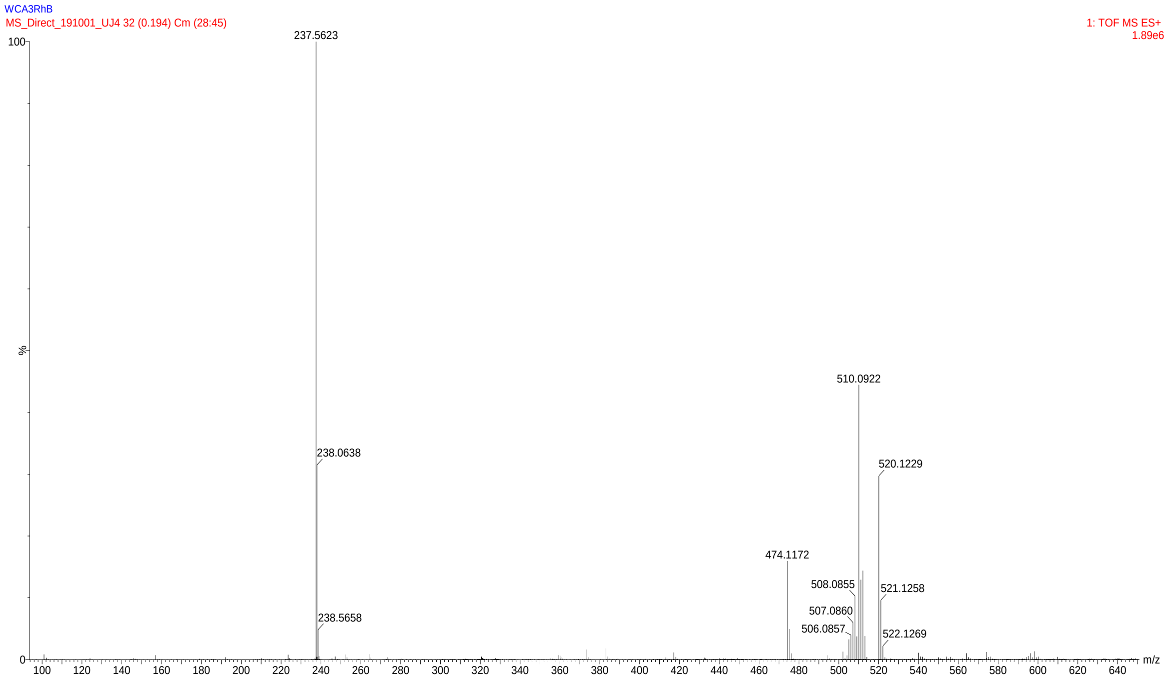

Figure S60: HRMS (ESI$^+$) of complex **C10.**

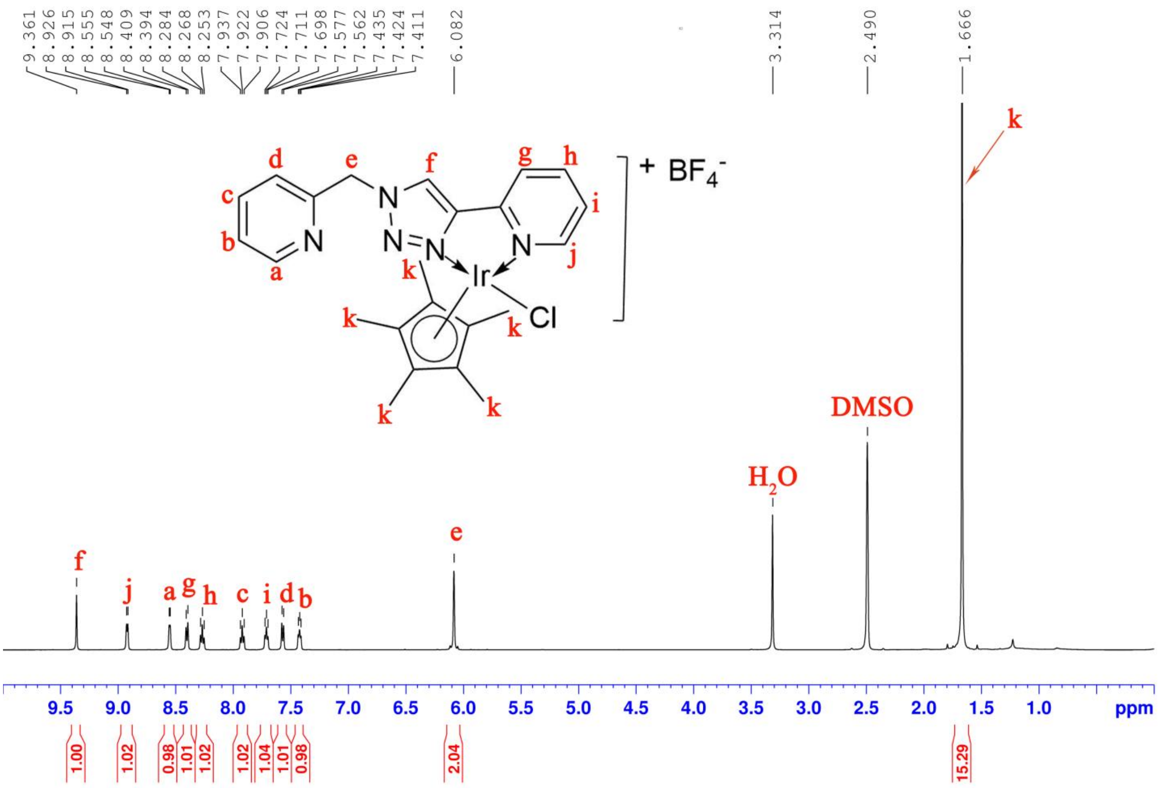

Figure S61: ^{1}H NMR spectrum of complex **C11** in DMSO-d_6.

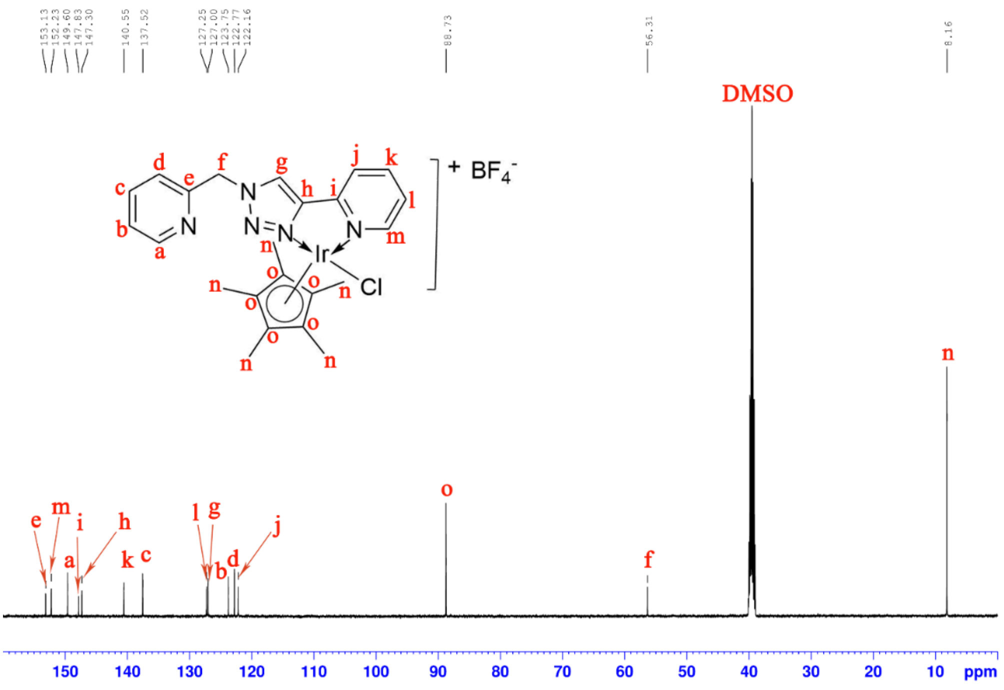

Figure S62: $^{13}C\{^{1}H\}$ NMR spectrum of complex **C11** in DMSO-d_6.

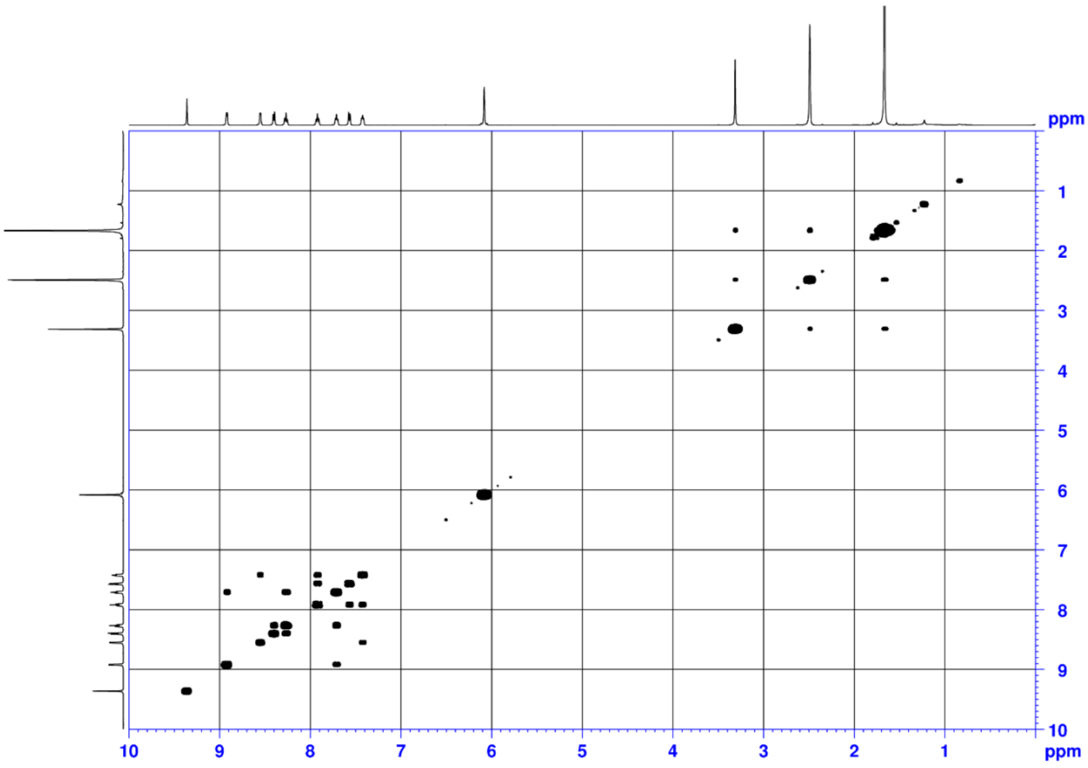

Figure S63: ^{1}H–^{1}H COSY NMR spectrum of complex **C11** in DMSO-d_6.

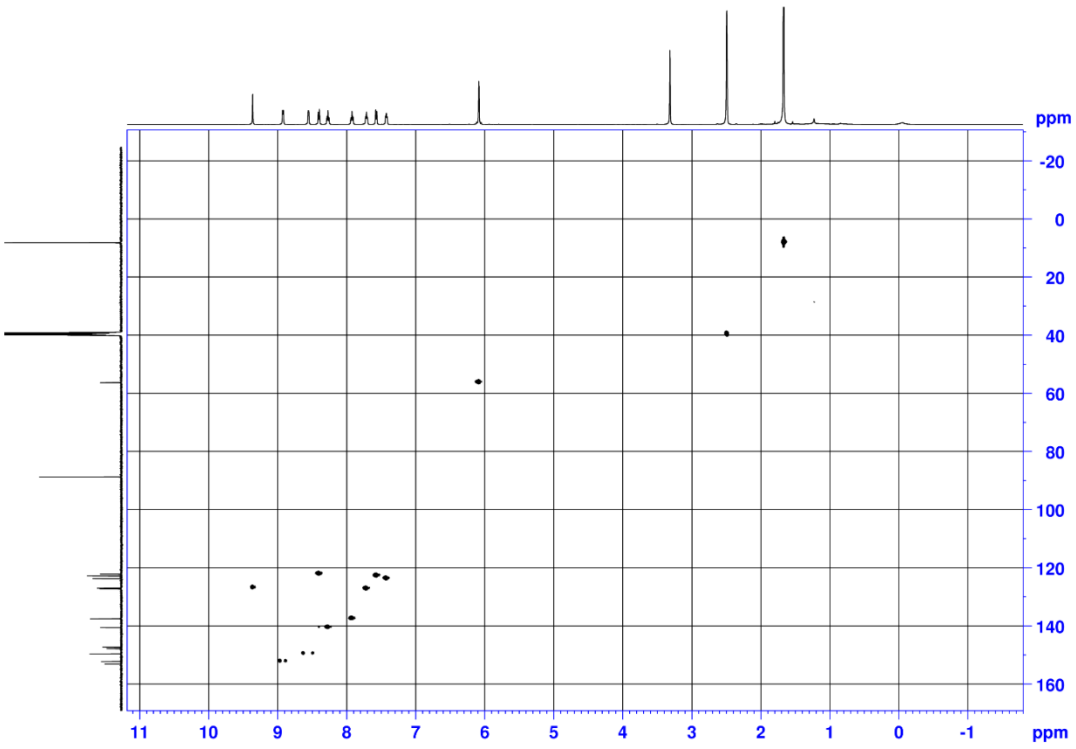

Figure S64: ^{1}H–^{13}C HSQC NMR spectrum of complex **C11** in DMSO-d_6.

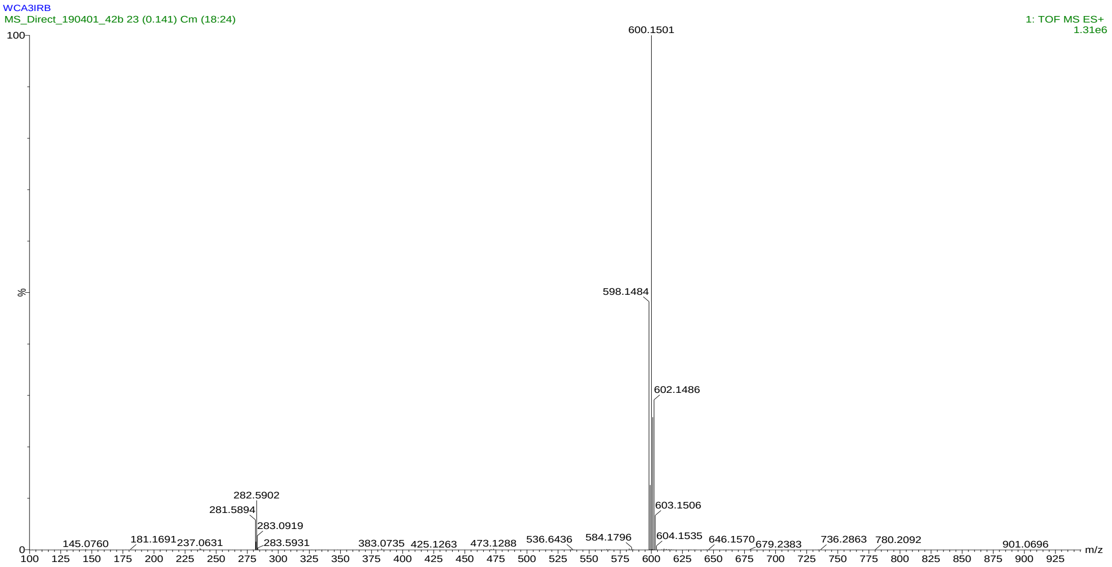

Figure S65: HRMS (ESI$^+$) of complex **C11.**

Table S1: Crystallographic parameters and data for complexes **C4** and its chloride counter ion analogue (**C4$^+$ Cl$^-$**)

**Parameter**	**Complex**	
	**C4$^+$Cl$^-$**	**C4**
**Empirical formula**	$C_{24}H_{27}Cl2N_4Rh$	$C_{24}H_{27}BClF_4N_4Rh$
**Formula weight**	545.31	596.67
**Temperature/K**	100	100
**Crystal system**	monoclinic	monoclinic
**Space group**	C2/c	$P2_1/n$
**a/Å**	34.792(4)	12.886(10)
**b/Å**	8.7695(9)	11.540(8)
**c/Å**	20.675(2)	17.048(12)
**α/°**	90	90
**β/°**	92.850(3)	97.303(12)
**γ/°**	90	90
**Volume/Å^3**	6300.3(11)	2514(3)
**Z**	66	21
**ρ_{calc} g/cm^3**	2.877	1.576
**μ/mm^{-1}**	4.923	0.835
**F(000)**	5016.0	1208.0
**Crystal size/mm^3**	0.4 × 0.3 × 0.2	0.4 × 0.3 × 0.2
**Radiation**	MoKα (λ = 0.71073)	MoKα (λ = 0.71073)
**2Θ range for data collection/°**	2.344 to 52.14	3.742 to 52.262
**Index ranges**	$-42 \le h \le 42$, $-10 \le k \le 10$, $-24 \le l \le 25$	$-15 \le h \le 15$, $-14 \le k \le 13$, $-21 \le l \le 21$
**Reflections collected**	50906	27625
**Independent reflections**	6230 [R_{int} = 0.1345, R_{sigma} = 0.0816]	5008 [R_{int} = 0.1507, R_{sigma} = 0.1157]
**Data/restraints/parameters**	6230/0/357	5008/0/321
**Goodness-of-fit on F^2**	1.043	1.036
**Final R indexes [I>=2σ (I)]**	R_1 = 0.0465, wR_2 = 0.1022	R_1 = 0.0606, wR_2 = 0.1516
**Final R indexes [all data]**	R_1 = 0.0681, wR_2 = 0.1122	R_1 = 0.0962, wR_2 = 0.1717
**Largest diff. peak/hole / e Å^{-3}**	0.94/-0.81	1.11/-0.71

Catalysis data: Hydrogenation of pyruvate

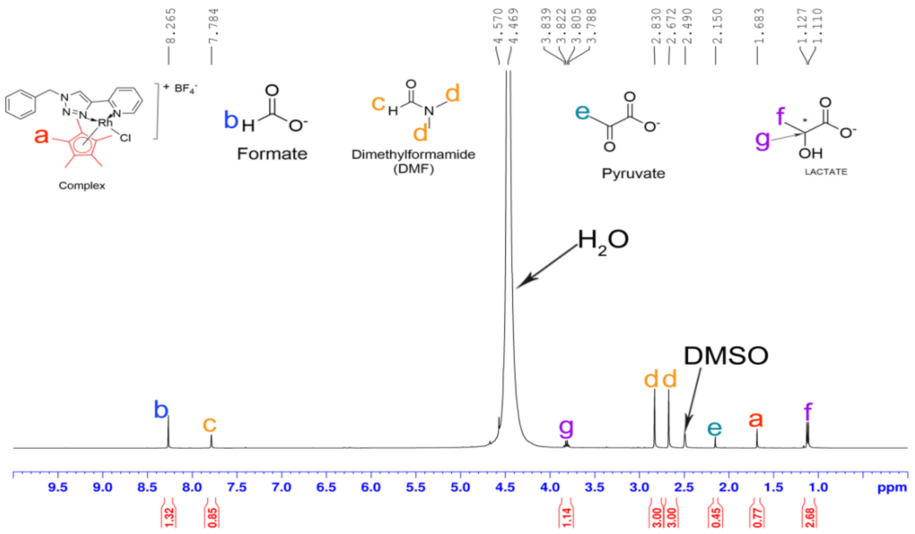

Figure S66: ^{1}H NMR spectrum for the hydrogenation of pyruvate to lactate obtained from crude catalysis mixture using DMSO-d_6 (1:1 v/v), and using DMF as an internal standard.

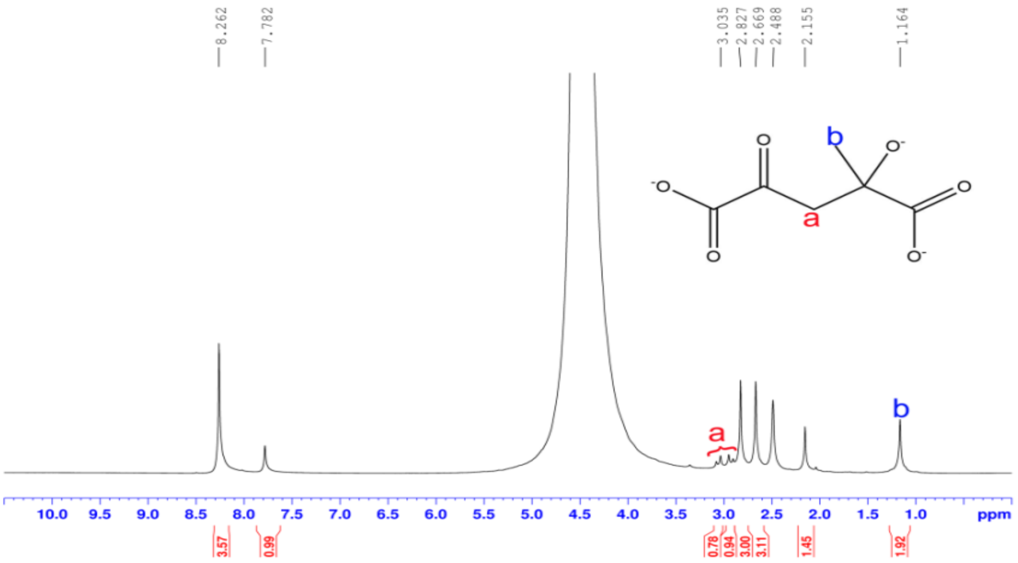

Figure S67: ^{1}H NMR spectrum showing base catalyzed (no catalyst used) dimerization of pyruvate to 2-methyl-2-oxido-4-oxopentanedioate obtained from crude catalysis mixture and DMSO-d_6 (1:1 v/v) and using DMF as an internal standard.

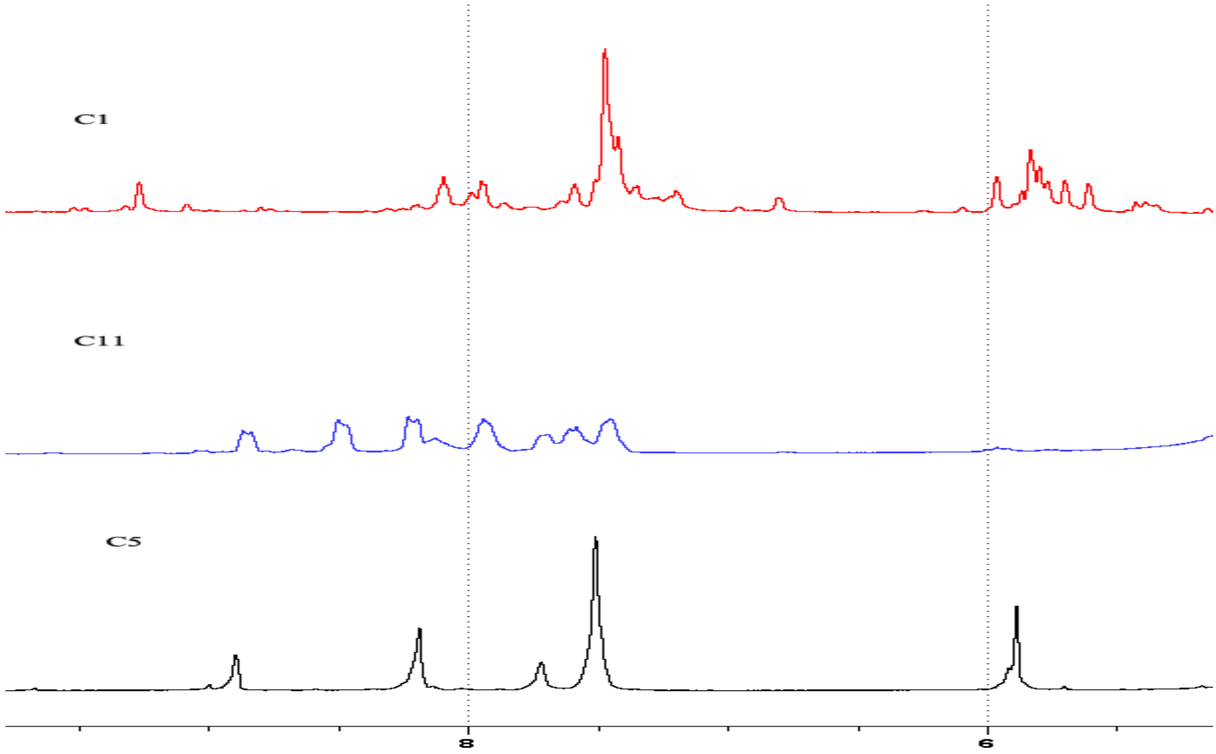

Figure S68: ^{1}H NMR spectrum of complexes **C1**, **C5** and **C11** in the presence of excess triethylamine over 21days. NMR spectrum was obtained in phosphate buffered D_2O and chemical shifts (δ) are reported in parts per million (ppm)

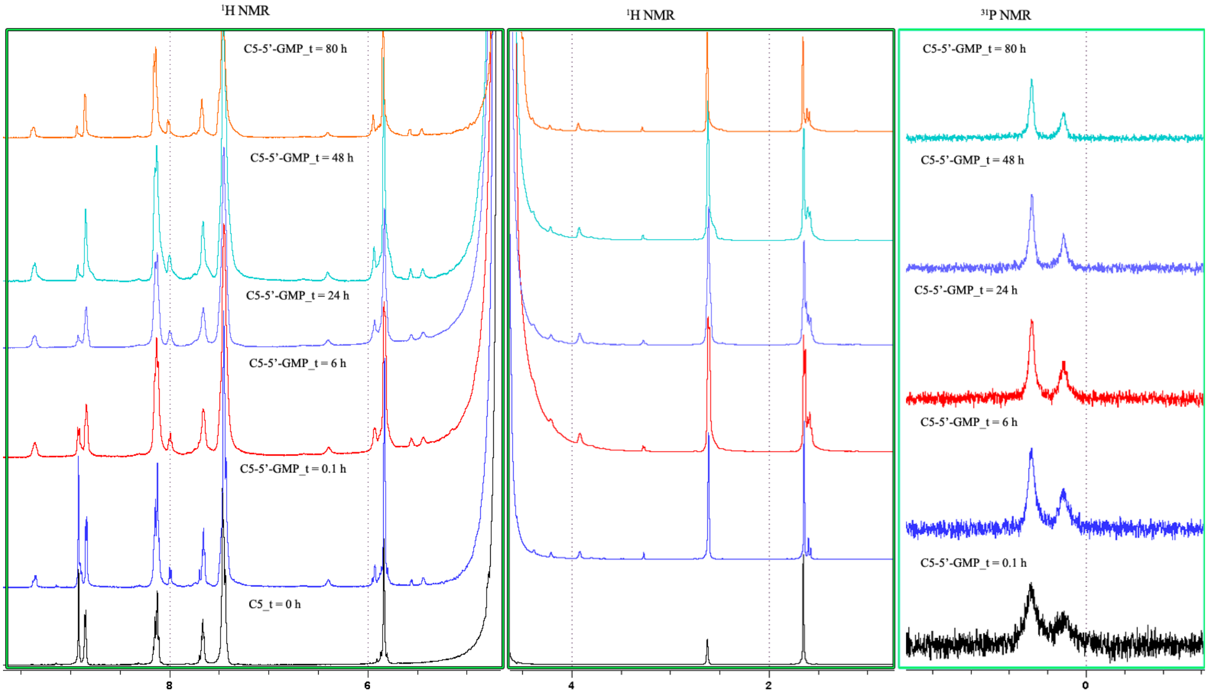

Figure S69: ^{1}H NMR spectra (column a and b) and ^{31}P{^{1}H} NMR spectra (column c) for the time dependent interaction of **C5** and 5'-GMP over 80 h. NMR spectrum was obtained in phosphate buffered D_2O and chemical shifts (δ) are reported in parts per million (ppm) The phosphorus peaks for **C5** shifted from 2.91 ppm and 1.10 ppm at t = 0 h to 2.81 ppm and 1.14 ppm at t = 80 h.

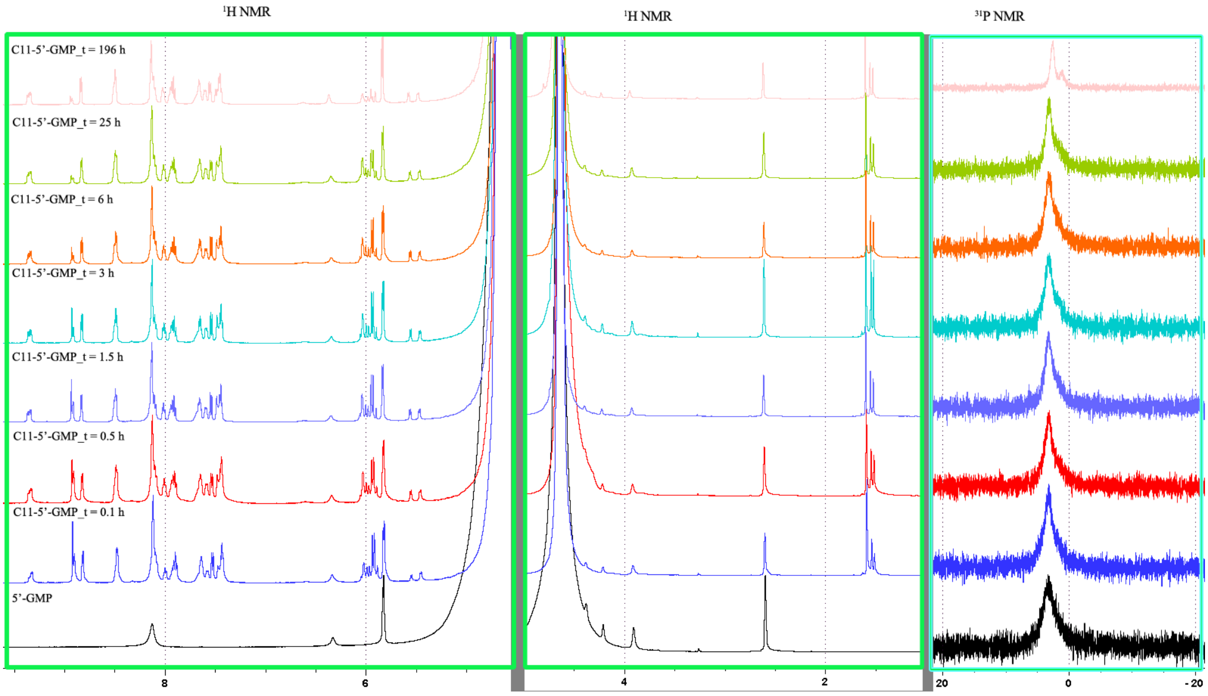

Figure S70: ^{1}H NMR spectra (column a and b) and $^{31}P\{^{1}H\}$ NMR spectra (column c) for the time dependent interaction of **C11** and 5'-GMP over 196 h. NMR spectrum was obtained in phosphate buffered D_2O and chemical shifts (δ) are reported in parts per million (ppm). Here, both phosphorus peaks did not resolve until time t = 80 h. At time t = 0 h, a single peak was observed at 3.0 ppm but at time t = 80 h 2 peaks could be observed at 2.7 ppm and 1.2 ppm.

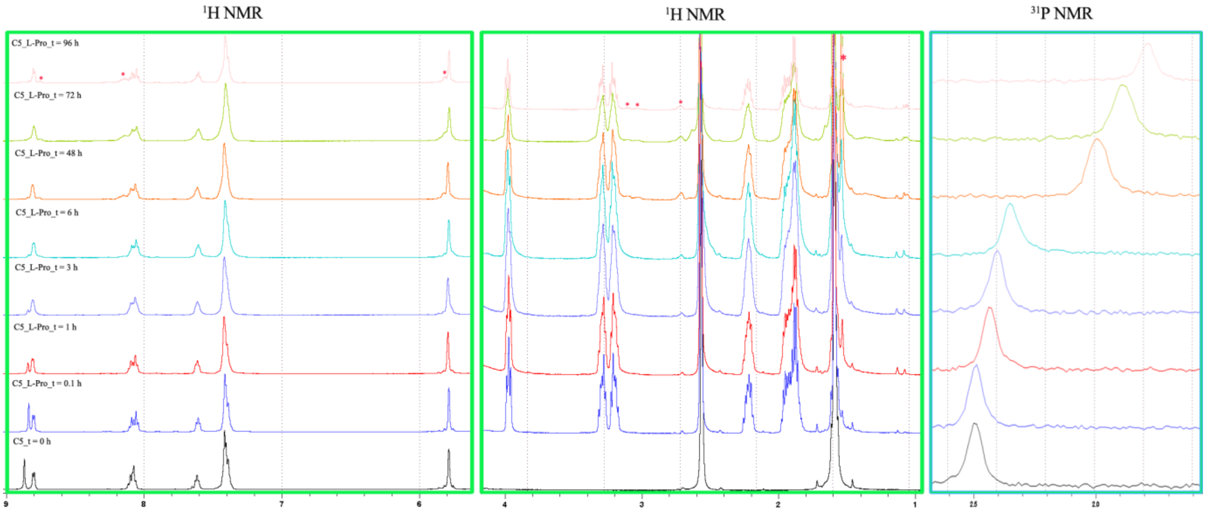

Figure S71: ^{1}H NMR spectra (column a and b) and $^{31}P\{^{1}H\}$ NMR spectra (column c) for the time dependent interaction of complex **C5** with L-proline. NMR spectrum was obtained in phosphate buffered D_2O and chemical shifts (δ) are reported in parts per million (ppm).

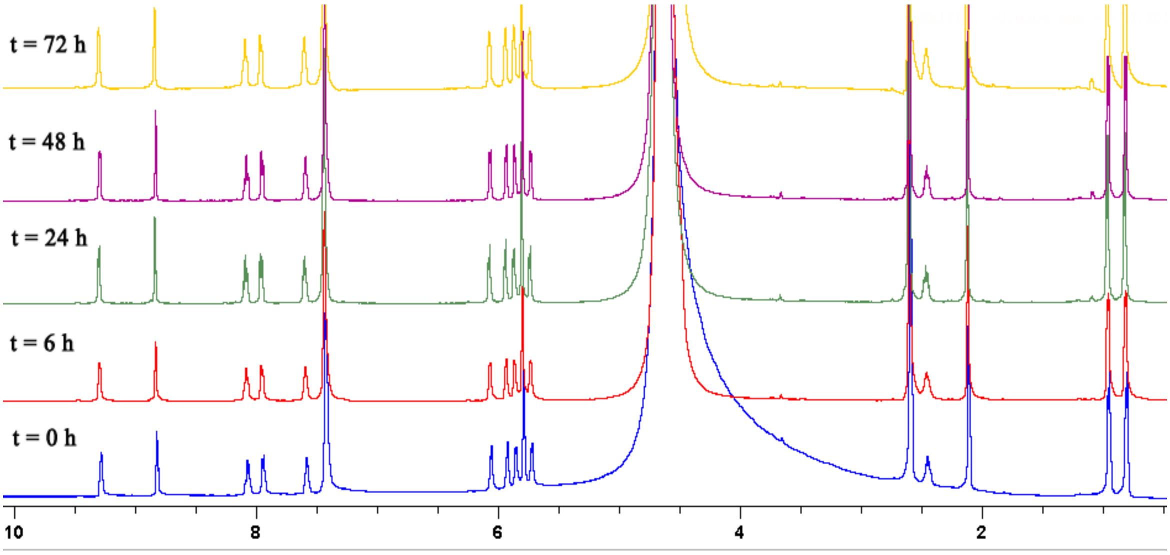

Figure S72: ^{1}H NMR spectra of complex **C1** in 10 % DMSO-d_6-phosphate buffered D_2O (pH 7.4) monitored over 72 h. Chemical shifts (δ) are reported in parts per million (ppm)

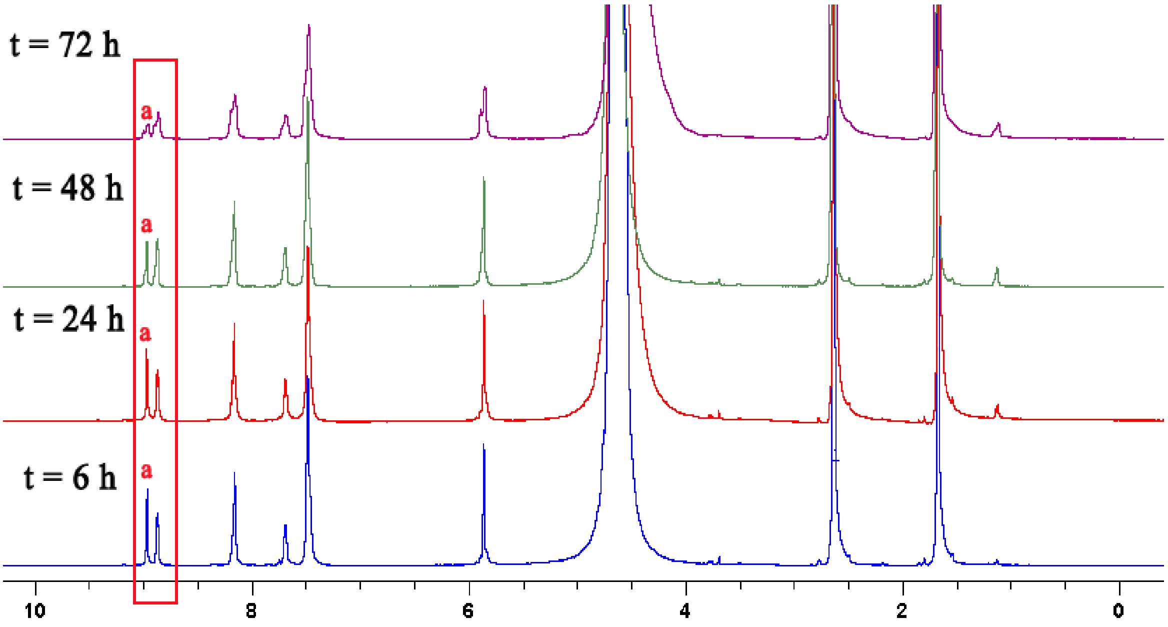

Figure S73: ^{1}H NMR spectra of complex **C5** in 10% DMSO-d_6-phosphate buffered D_2O (pH 7.4) monitored over 72 h. Chemical shifts (δ) are reported in parts per million (ppm). Spectrum at t = 0 h could not be obtained as sample failed to shim.

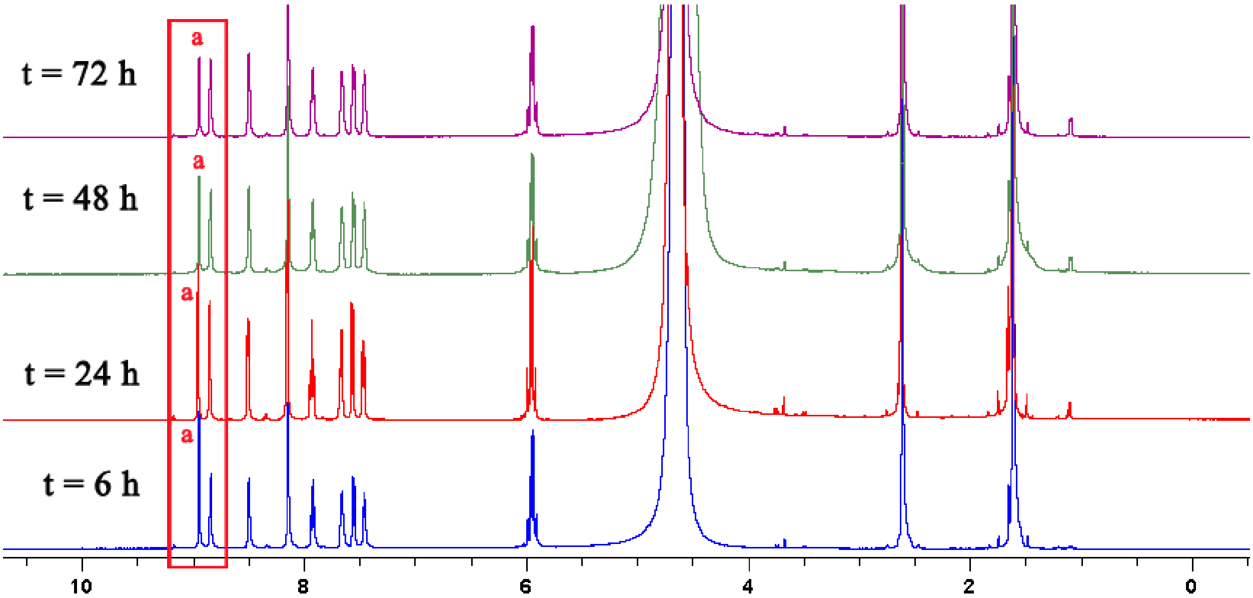

Figure S74: ^{1}H NMR spectra of complex **C11** in 10% DMSO-d_6-phosphate buffered D_2O (pH 7.4) monitored over 72 h. Chemical shifts (δ) are reported in parts per million (ppm). Spectrum at t = 0 h could not be obtained as sample failed to shim.

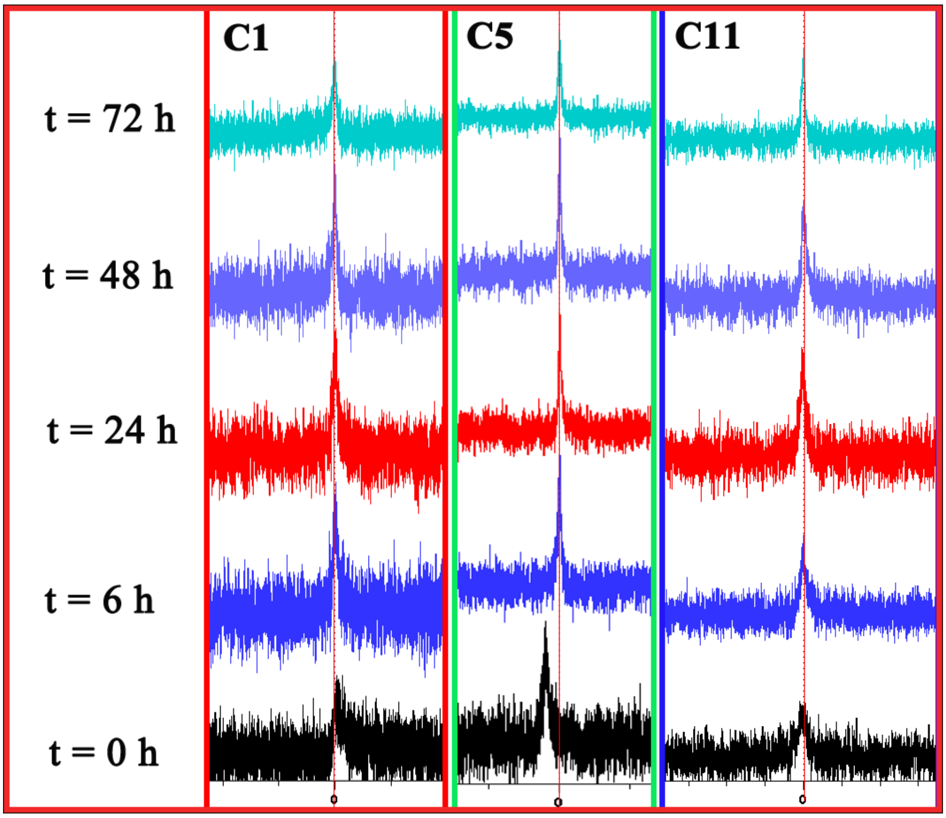

Figure S75: ^{31}P NMR spectra of complexes **C1**, **C5** and **C11** in 10% DMSO-d_6-phosphate buffered D_2O (pH 7.4) monitored over 72 h. Chemical shifts (δ) are reported in parts per million (ppm). For complexes **C5** and **C11** sample did not shim at time t = 0 h and this might have contributed to the sparse shift observed with complex **C5** at t = 0 h.

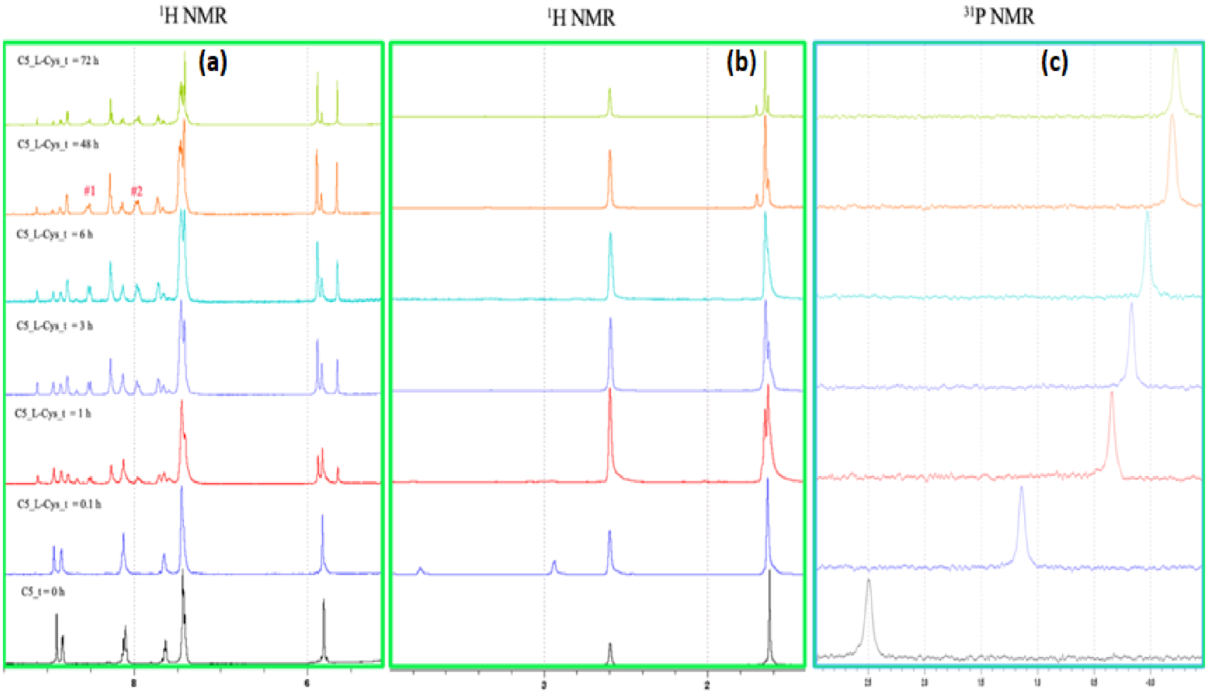

Figure S76: ^{1}H NMR spectra (column a and b) and ^{31}P{^{1}H} NMR spectra (column c) for the time dependent interaction of **C5** with L-Cysteine. NMR spectrum was obtained in 10% DMSO-d_6-phosphate buffered D_2O (pH 7.4) and chemical shifts (δ) are reported in parts per million (ppm).

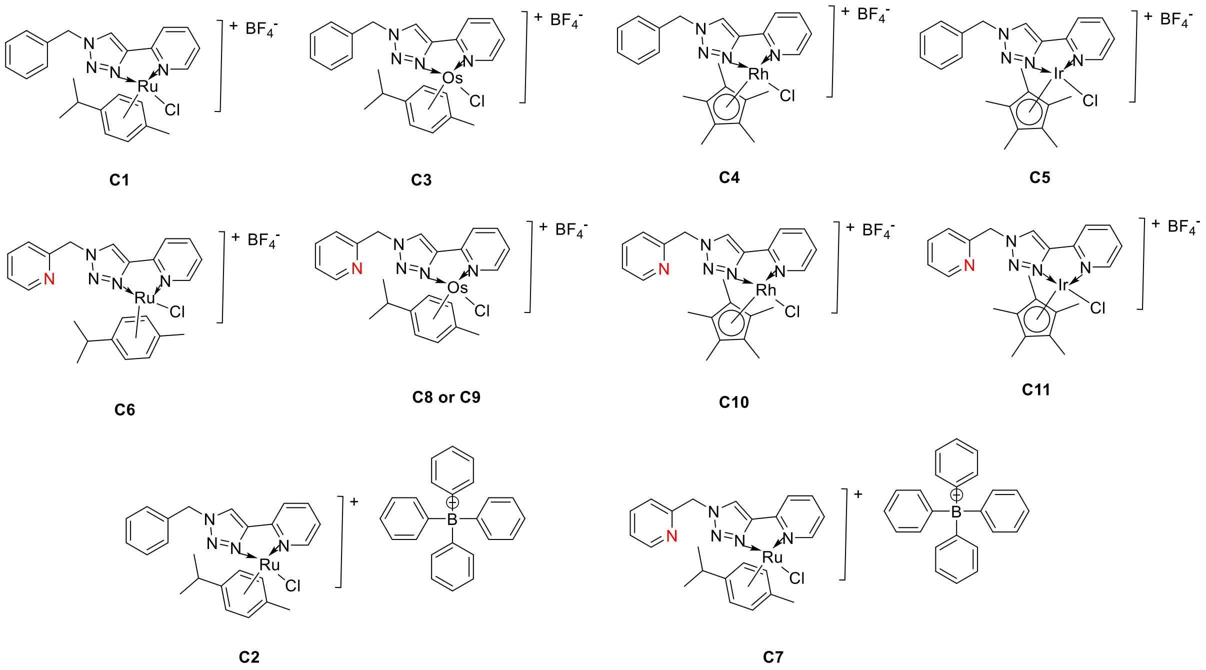

Figure S77: Full structure of synthesized complexes **C1-C11**

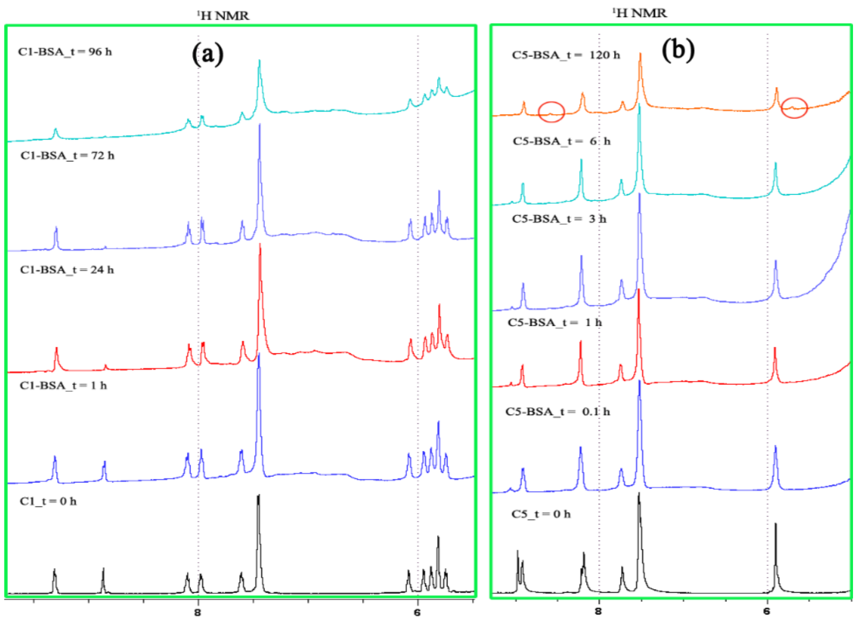

Figure S78: ^{1}H NMR spectra for the time-dependent interaction of BSA with (a) **C1** and (b) **C5** in 10% DMSO-d_6-phosphate buffered D_2O (pH 7.4) Chemical shifts (δ) are reported in parts per million (ppm).

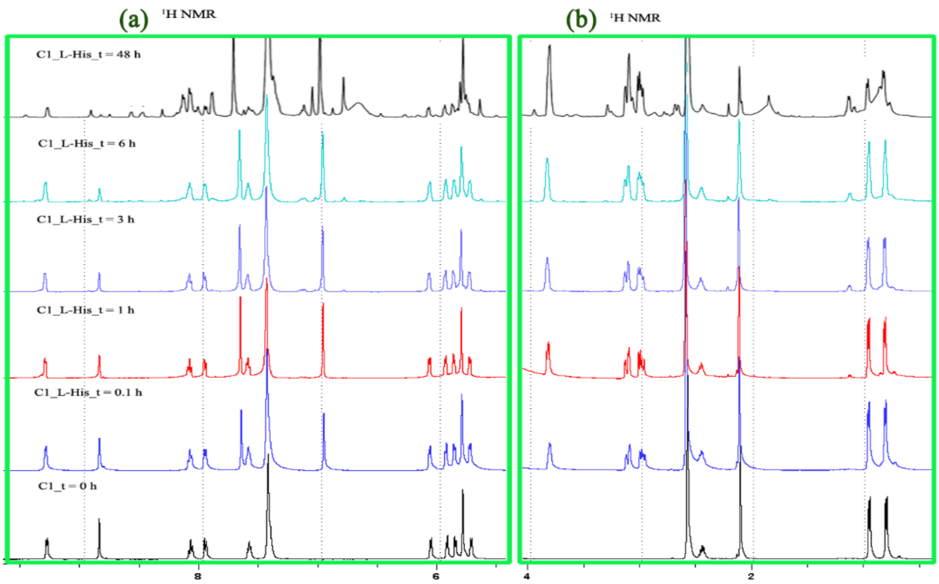

Figure S79: ^{1}H NMR spectra (column a and b) for the time-dependent interaction of complex **C1** with L-Histidine in 10% DMSO-d_6-phosphate buffered D_2O (pH 7.4). Chemical shifts (δ) are reported in parts per million (ppm).

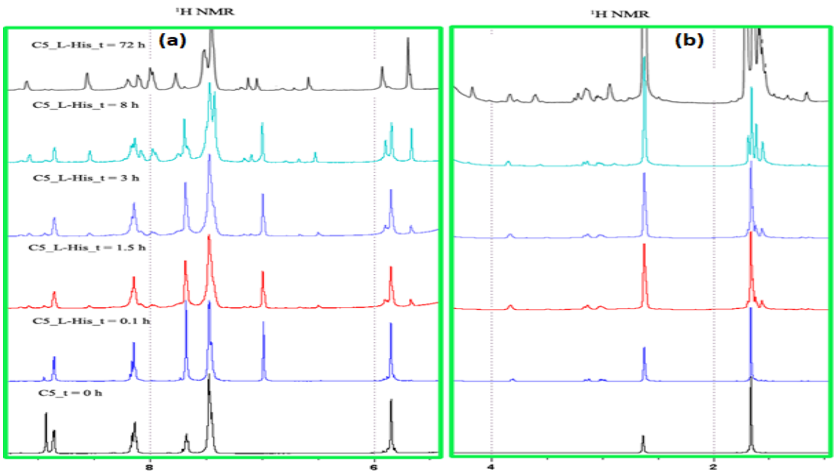

Figure S80: ^{1}H NMR spectra (column a and b) for the time-dependent interaction of complex **C5** with L-Histidine in 10% DMSO-d_6-phosphate buffered D_2O (pH 7.4). Chemical shifts (δ) are reported in parts per million (ppm).

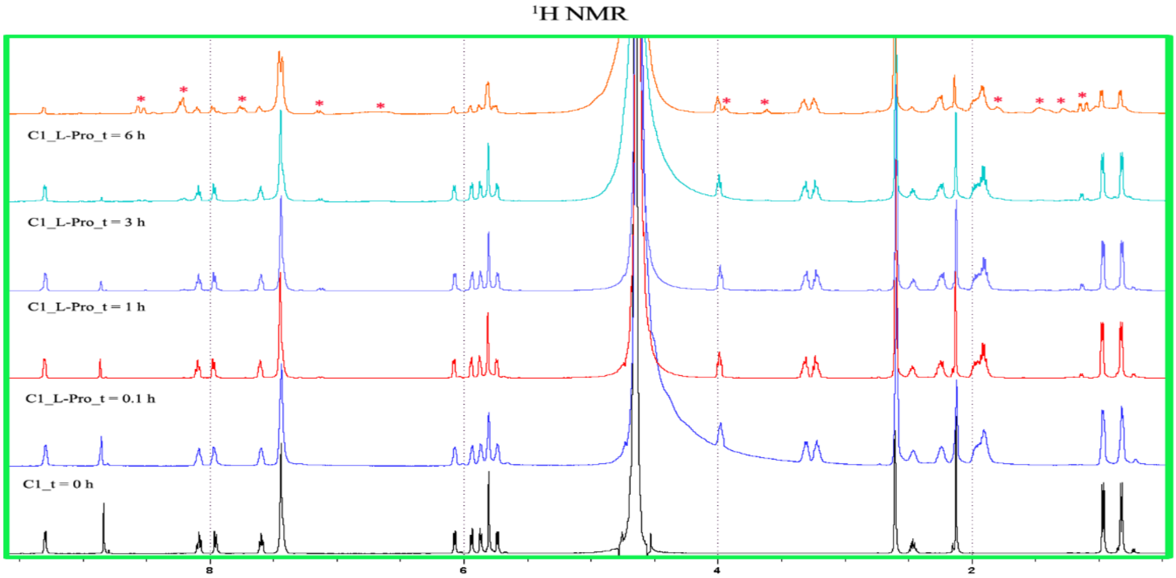

Figure S81: ^{1}H NMR spectra for the time-dependent interaction of **C1** with L-proline in 10% DMSO-d_6-phosphate buffered D_2O (pH 7.4)Chemical shifts (δ) are reported in parts per million (ppm).

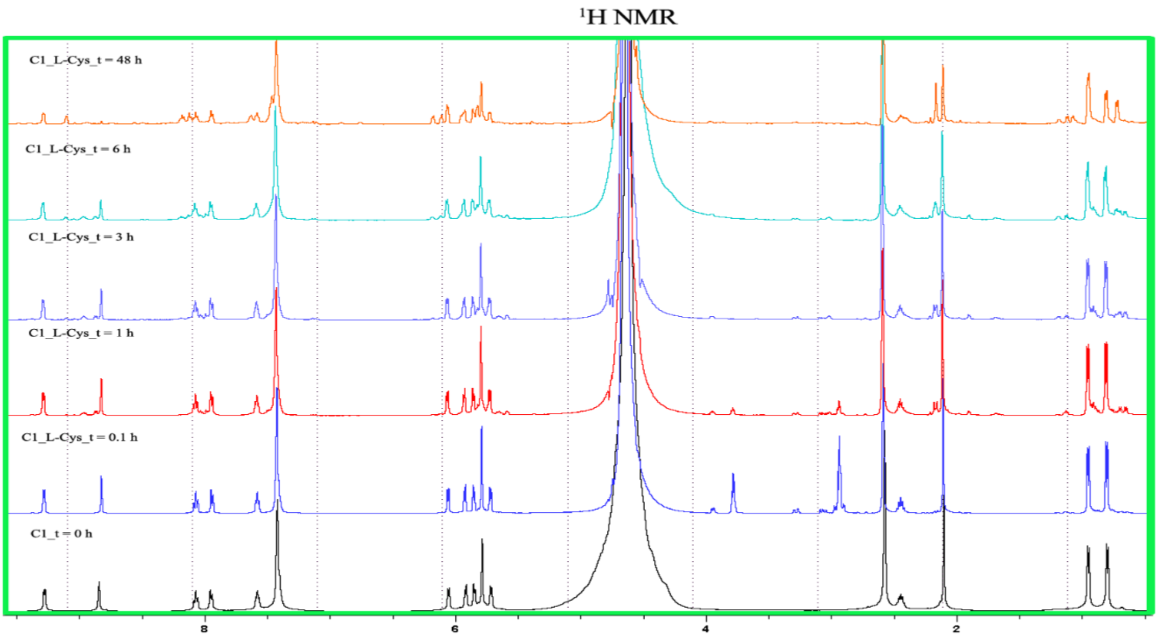

Figure S82: ^{1}H NMR spectra for the time dependent interaction of **C1** with L-cysteine in 10% DMSO-d_6-phosphate buffered D_2O (pH 7.4). Chemical shifts (δ) are reported in parts per million (ppm).

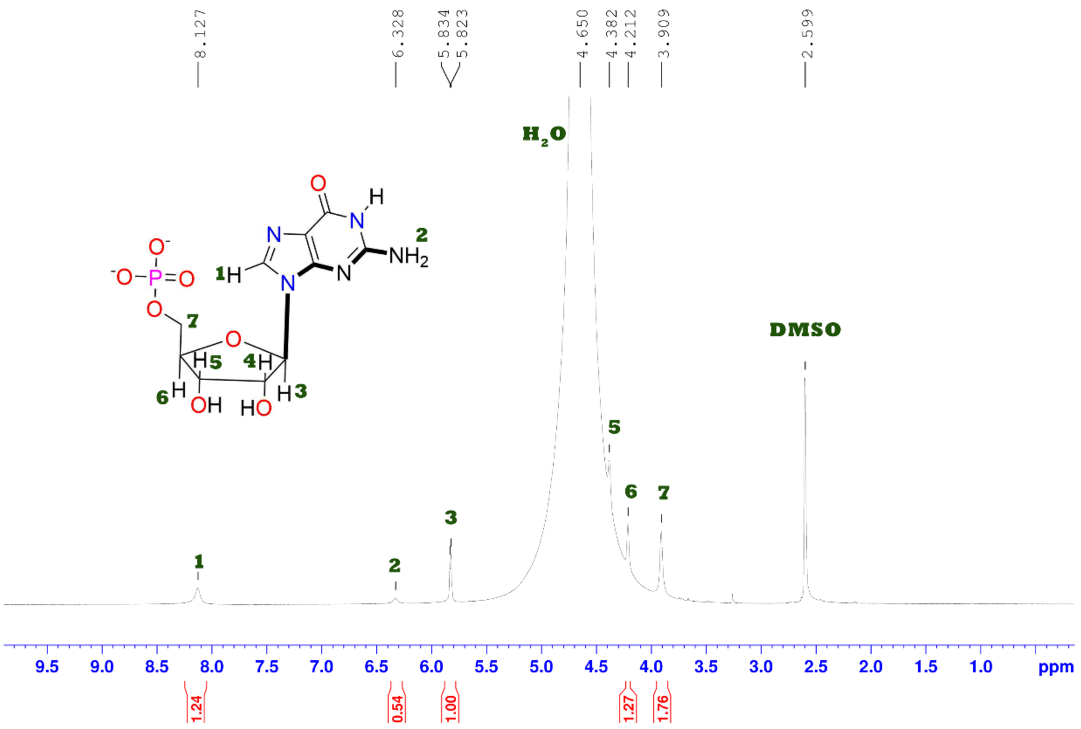

Figure S83: Annotated ^{1}H NMR of guanosine-5’-monophosphate (5’-GMP) in 10% DMSO-d_6-phosphate buffered D_2O (pH 7.4).
